# Supplementary material for: Prominent members of the human gut microbiota express endo-acting O-glycanases to initiate mucin breakdown
Source: Nat Commun. 2020 Aug 11;11:4017. doi: 10.1038/s41467-020-17847-5 (PMC7419316; doi:10.1038/s41467-020-17847-5)
Supplement: Supplementary file 1 — Supplementary Information [file 41467_2020_17847_MOESM1_ESM.pdf]

Prominent members of the human gut microbiota express endo-acting O-glycanases to initiate mucin breakdown

Supplementary Information

Crouch *et al.*

Supplementary Discussion

Mucin structure in the gut

The mucus of the colon is composed of two layers. The dense inner mucus layer is an abiotic environment formed by Muc2 still attached to the luminal epithelial cells to protect them from any contact with the HGM[1]. The upper layer is formed from Muc2 that has been released from the inner layer and is niche for some species of the HGM that can access mucins as a nutrient source[1]. The upper layer is renewed from the inner layer every 1-2 hours[2, 3] and an estimated 3-4 g/day of mucin is fermented by the HGM[4]. There is a close association between the HGM and mucin production by the host. This has been demonstrated in germ-free mice where the mucin was observed to remain attached to the goblet cell rather than being released[5]. These findings indicate that the HGM promotes the production of healthy mucus barrier and highlights the mutualistic relationship between host and HGM[6].

Previous characterisation of the O-glycans of Muc2 from human sigmoid colon samples showed over 100 different structures, but general trends included mono- to tri-sialyation, predominantly core 3 structures, sulfation predominantly on galactose, fucose predominantly on GlcNAc (both Lewis a and x structures, decorating the chains rather than capping), low occurrence of blood group sugars and elongation of up to three LacNAcs[7]. Blood group epitopes have been found to be more common in other mucins, such as salivary, respiratory and cervical[8, 9, 10]. Stomach mucin is predominantly Muc5AC and Muc6 and both are characterised as having a capping α1,4-GlcNAc[11], but only Muc5AC has Lewis b structures[12]. A large diversity in chain length and composition of gastric O-glycans has also been observed, with many structures seemingly specific to an individual[13].

Mucins as substrate for microbial cultures

Commercially available PGMs (Sigma) dissolved in pure water were too opaque to monitor growth by optical density. However, centrifugation to remove precipitate produced a substrate that was clear enough to monitor growth at OD_{600}. Approximately 70 and 80 % of the original dry mass remained in the soluble portion of PGM type II and III, respectively. Incubations of an O-glycan active enzyme against the precipitate and soluble fractions showed that the majority of accessible substrate was in the soluble fractions (Supplementary Fig. 22). The soluble fraction and precipitate were also incubated with polysaccharide lyases previously characterised or predicted to have activity against the glycosaminoglycans (GAGs) Hep, CS and HA[14, 15, 16]. The results indicate that there is some GAG contamination of the PGM soluble fraction (Supplementary Fig. 22)

Transcriptomic and proteomic data used to identify the GH16 O-glycanases

Two *in vitro* transcriptomic datasets were used for *B. thetaiotamicron* from two different points of growth (early and late phase) on mucin O-glycans from PGM type III relative expression levels on glucose[16] (Supplementary Fig. 1). For *B. fragilis* and *B. caccae*, transcriptomes from cells grown on mucin O-glycans from PGM III were compared to glucose grown cells[17, 18] (Supplementary Fig. 2). A 10-fold cut-off was used to select the mucin upregulated transcripts, similar to that applied by the authors of these studies. We expanded this pool of selected genes to include the whole of the predicted PULs these genes are part of. A possible limitation with using a relatively high 10-fold cut-off for upregulation data is the potential to miss genes that have high basal expression that may also be involved in mucin breakdown.

Four different transcriptomic datasets were used to look at upregulated genes from *A. muciniphila* (Supplementary Fig. 3). Strains ATCC BAA835 or DSM22959 were used but these are the same strains from different culture collections. Two datasets originated from the same report, one recorded *A. muciniphila* genes upregulated during growth on purified mucin O-glycans from PGM III versus growth on GlcNAc and the other between a fibre-rich diet and a fibre-free diet in mice (where the bacteria in the mouse gut were shown to excessively degrade the mucus layer)[18]. Two further *A. muciniphila* transcriptomic studies used purified PGM III, but with the O-glycans still attached to the protein (one compared upregulated genes to cells grown on glucose and one vs cells grown on GlcNAc)[19, 20]. For all these datasets we used a starting point of 5-fold upregulation to identify transcripts potentially involved in mucin breakdown. The fold increase in upregulation was lower to that observed for *Bacteroides* spp., although it is not clear why this is. The analysis of these *A. muciniphila* transcriptomics data sets generated the pool of genes and we included data for all these genes/proteins across all the data sets analysed where it was available.

Two proteomic datasets were also used for *A. muciniphila*, both used cells grown on untreated PGM III versus glucose grown cells. One reported total protein, while the other only the outer membrane fraction of the same cells[21]. I*n vivo* data for *A. muciniphila* was included as this was much less complex than the equivalent data for *Bacteroides* spp., mainly due to the ability of *Bacteroides* to target multiple dietary glycans, thus complicating the identification of mucin-acting CAZymes. *A. muciniphila* has a narrow substrate range and much fewer CAZymes than the *Bacteroides* spp analysed.

Sequence identity and molecular architecture of the GH16 enzymes

The sequence identity between the nine mucin-upregulated GH16 family members was mostly relatively low with values of between 24-34 % (Supplementary Table 2). The exceptions to this were two pairs of *B. fragilis* and *B. caccae* enzymes in homologous PULs: BF4058 and BACCAC_02679 and BF4060 and BACCAC_02680 with 87 and 79 % identity, respectively

(Fig. 2). The enzymes were predicted to be composed of a single catalytic module only, with the exception of BT2824, which also possesses an N-terminal module of uncharacterised function (DUF4971; Fig. 2). Signal peptide (SP) predictions using SignalP 5.0 revealed that four of the six *Bacteroides* GH16 family members were predicted to have a Type II signal peptide, suggesting surface localisation, while the closely related BF4060 and BACCAC_02680 were predicted to be SPI and are thus likely periplasmic (Supplementary Table 1). All three *A. muciniphila* GH16 enzymes are most likely SPI, indicative of periplasmic localisation, although Amuc_0724 had a split SPI/II prediction likely due to the two cysteine residues near the end of the signal sequence. Furthermore, Amuc_2108 has been shown experimentally to be localised to the outer membrane[21]. Inspection of the Amuc_2108 sequence reveals a C-terminal hydrophobic region followed by a highly positively charged sequence after this, indicative of a potential TM anchor and surface localisation.

Growth of human gut microbes on mucin

Prominent human gut *Bacteroides* species and *A. muciniphila* were tested for their ability to grow on PGM as the sole carbon source (Supplementary Fig. 23). As well as *B. caccae*, *B, fragilis* and *B. thetaiotaomicron*, five additional *Bacteroides* species were tested to provide an indication of how common mucin degradation is within this genus and to compare against the known mucin degraders that were the focus of this study. Of the 8 *Bacteroides* species tested, all could grow to some extent on PGM types II and III prepared as described above. *A. muciniphila* consistently grew to a higher OD than the *Bacteroides* species indicating that it can access a greater proportion of the glycoprotein. Growth of the *Bacteroides* species on PGM type III was commonly biphasic, suggesting either multiple substrates within the mucin prep, or that the breakdown of the mucin molecule occurs in distinct steps. Related to the former of these possibilities, mucin preparations are likely contaminated with other host polysaccharides from glycocalyx sources that are difficult to completely remove, including glycosaminoglycans (GAGs) such as chondroitin sulfate (CS), hyaluronic acid (HA), heparin (Hep) and heparan sulfate (HS). Incubation of PGM with the polysaccharide lyases from the *B. thetaiotaomicron* PULs known to degrade these GAGs[14, 15] show these acidic polysaccharides are present (Supplementary Fig. 23). However, growth on PGM of a mutant *B. thetaiotaomicron* strain, where the PULs required for the degradation of Heparin/heparan sulfate and CS/HA have been knocked out (ΔHS and ΔCS strains, respectively) shows little difference in growth profile relative to the wild-type (Supplementary Fig. 23). There is slightly less initial growth, but the biphasic trend remains, suggesting the breakdown of mucin is responsible for this growth phenotype, rather than GAG contamination. Furthermore, some species show very little growth on GAGs, but do grow on PGM (e.g. *B. fragilis* and *B. vulgatus*).

Prioritising utilisation of GAGs over mucins has been observed previously in *B. thetaiotaomicron*[16].

Phylogenetic analysis of GH16 family members

Phylogenetic analysis was carried out to explore the relationship between the nine mucin-upregulated GH16 family members characterised here and previously characterised non-mucin GH16 enzymes (Supplementary Fig. 5). The data show that the newly identified mucin-upregulated GH16 family members stem out of β-glucanases and are not associated with any of the GH16 family members with activities on β-galactans, xyloglucan or chitin β1,6 glucosyltransferases. Notably, there was significant clustering of the GH16 family member's active on O-glycans.

Further phylogenetic analysis was carried out using all known GH16 subfamily 3 members (Supplementary Fig. 6). The *Bacteroides plebius* enzymes with activities against porphyran and agarose from subfamilies 12 and 16, respectively, were also included for comparison. The branch with most of the O-glycanase GH16 enzymes is solely composed of sequences from microbes residing in animal GI tracts or oral cavity. Potential pathogens cluster here, such as *Elizabethkingia* spp., *Capnocytophaga* spp. and *Tannerella forsythia*. Furthermore, the keratan sulfate β-galactanase from *Sphingobacterium multivorum* is also in this cluster[22, 23]. The O-glycanases characterised in this report are the third example of β-galactanase activities in this family. Interestingly, Amuc_0724 and Amuc_0875 cluster in a different branch with the GH16 enzymes from *Bacteroides* spp. active on the β1,3-galactan backbone of plant arabinogalactan[24]. This raises the question of whether the Amuc_0875 is a true O-glycanase, especially as it has relatively poor activity against mucin glycans compared to the other GH16 enzymes characterised in this study. However, the gene encoding Amuc_0875 is found to be upregulated in the multiple transcriptomic datasets available of *A. muciniphila* during growth on mucins and the organism is unable to grow on arabinogalactans[18], raising the possibility that the target substrate for Amuc_0875 is a mucin-derived glycan that has yet to be identified. Interestingly, the composition of the different *Bacteroides* PULs containing the mucin-upregulated GH16 genes are highly variable (Fig. 2). Furthermore, there are other GH16 enzymes in *B. thetaiotaomicron*, *B. fragilis* and *B. caccae* that have relatively high sequence homology to the O-glycanases characterised here that are not upregulated during growth on mucins in any of the transcriptomics data analysed. For example, BF4139, BACCAC_00302 and BT2550 all have ~60 % identity to BACCAC_03717. The genes encoding these enzymes may be differently regulated to the GH16 family members characterised here, or the enzymes target different parts of the mucin glycoprotein, or target a different glycan altogether.

Exo-glycosidase characterisation of GH16 oligosaccharide products released from SI mucin

To investigate the structures of the oligosaccharide released by the GH16 enzymes in more detail, the products of a BF4058 digest of SI mucin were treated with a series of exo-acting glycosidases of known specificity (Supplementary Fig. 8). BF4058 was chosen due to its predicted surface localisation and thus is likely involved in the initial extracellular breakdown of mucins. The data contributes to the understanding of the detailed specificity of the GH16 O-glycanases and also how endo- and exo-acting CAZymes can be used in combination to explore the structures of the mucin O-glycans.

The combination of a broad acting α1,2 fucosidase from *Bifidobacterium bifidum*[25] (see Supplementary Fig. 24 for specificity of fucosidase) and BF4058 (both with and without sialidase) resulted in an increase in the types of glycan structure that could be detected, in the form of higher degree of polymerisation (dp) oligosaccharides (Supplementary Fig. 8). This indicates that removal of some fucose from mucin allows the O-glycanase to access more substrate. Fucose still remaining on glycans after the α1,2-fucosidase treatment (e.g. *glycans 6, 9 and 11)* is likely to either be α1,3 or α1,4 linked, which is also present in mucins. Interestingly, one of the putative α1,2 fucosidases from the *B. fragilis* upregulation data (BF0855, a GH95 family member; Supplementary Fig. 2a) has a Type II signal sequence, indicating it is located on the cell surface and thus could be involved in maximising access to mucin structures by BF4058^{GH16} prior to import.

Inclusion of additional exo-glycosidases to the BF4058, sialidase and fucosidase digests reveal further insight into the oligosaccharide structures released by the GH16 (Supplementary Fig. 8). The addition of a β1,4-galactosidase (BT0461^{GH2})[26] results on the disappearance of one of the *glycan 5* peaks, indicating this saccharide is capped with a β1,4-galactose. Both *glycan 5* peaks disappear with the addition of a β1,3/4-galactosidase (BF4061^{GH35}, see Supplementary Fig. 25 for BF4061 specificity), indicating the other *glycan 5* peak is capped with a β1,3-galactose (Supplementary Fig. 8, red asterisks). Addition of a broad-acting GlcNAc'ase (BT0459^{GH20})[26] results in the disappearance of *glycans 3, 4, 17, 21 and 23* (purple asterisks) indicating these structures are capped with β-GlcNAc.

Exo-acting CAZymes specific to either the α-GalNAc or α-galactose found on blood group A or B structures, respectively (see Supplementary Fig. 24 for activity of these exo-CAZymes), were also added to digests in combination with BF4058. No difference in glycans was observed when an α-galactosidase was added, but inclusion of an α-GalNAc'ase revealed several of the larger oligosaccharides could be further degraded, indicating these glycans have the capping α-GalNAc of Blood group A (Supplementary Fig. 8, green asterisks, *glycans 9, 11, 12, 14 and 23*).

Activity of the GH16 O-glycanases against defined oligosaccharides

A variety of defined O-glycan and human milk-derived oligosaccharides were used to assess the specificity of the nine GH16 enzymes further (Supplementary Figs. 9-12 and Supplementary Table 4). These data indicate that the enzymes are endo β1,4-galactosidases with a requirement for a β1,3-linked sugar at the -2 position. Notably, none of the GH16 enzymes require sulfation or fucosylation decorations for activity. Most of the GH16 enzymes hydrolysed the TriLacNAc rapidly under the conditions tested (Supplementary Figs. 9, 10 and 11a), however, BF4058 and BACCAC_02679 displayed significantly lower activity against the hexasaccharide and degradation of TriLacNAc by Amuc_0875 was only detectable after overnight incubation (Supplementary Fig. 11a).

All enzymes except Amuc_0875 were active against Lacto-N-neotetraose (LNnT) and Lacto-N-tetraose (LNT), suggesting plasticity in the positive subsites of these GH16 family members as glucose rather than GlcNAc will occupy the +1 subsite with these tetrasaccharides (Supplementary Fig. 11). However, substrate depletion assays indicate differences between the GH16 enzymes in terms of the importance of these subsites for activity (Supplementary Fig. 10). The activity against LNnT and LNT is comparable to TriLacNAc in the case of BACCAC_03717, for example, but is much lower for BF4060, BT2824, BACCAC_02680, Amuc_0724 and Amuc_2108. Furthermore, three of the enzymes showed preferences between the two milk oligosaccharides. BACCAC_02680 degrades LNT preferentially, whereas BT2824 and Amuc_0724 prefer LNnT. This may indicate a difference in preference for a β1,3 and a β1,4 linkages between the -2 and -3 subsites. Interestingly, when these GH16 family members were tested against a form of LNT where the GlcNAc is replaced with a GalNAc no activity was detected apart from trace activity for Amuc_0875 (Supplementary Fig. 11e). The difference between these sugars is the position of the hydroxyl at C4 being equatorial in GlcNAc and axial in GalNAc. An axial bond at this position means the β1,4-linked Gal would be at an angle relative to the rest of the oligosaccharide and the lack of activity observed for the GalNAc containing oligosaccharide suggests that this structure cannot be accommodated by most of the GH16 enzymes. The lack of an Amuc_0875 structure however precludes any structural insights into why this enzyme of the nine tested is the only one that displays any activity against the GalNAc-containing oligosaccharide. A requirement for a sugar in the -2 subsite has been documented previously for other GH16 enzymes[27, 28]. There is also evidence to suggest that a sugar in the -2 subsite is required for activity for most of the GH16 enzymes tested here. For example, the Galβ1,4GlcNAcβ1,4Gal product that remains after TriLacNAc, paraLacto-Nneohexaose, and LNnT degradation is not further degraded, although BT2824 and BACCAC_03717 do appear to have trace activity against this trisaccharide, suggesting the requirement for a sugar in the -2 subsite is not absolute in these enzymes (Supplementary Fig. 11c).

Activity against blood group hexasaccharides type II was then used to probe the plasticity the enzymes have in the negative subsites (Supplementary Figs. 10 & 11f-h). These glycans have a LNnT core with an α1,2-fucose and an α1,3-GalNAc or galactose (blood group A and B, respectively) on the galactose at the non-reducing end. Again different GH16 enzymes showed different preferences, but the activity of most of the enzymes were affected by these decorations relative to LNnT, with Amuc_0724 being the exception. Only BT2824 seemed to show a preference for the hydrolysis of blood group B over A. Blood group H was also tested to look at the effect of the α1,3-GalNAc or galactose on activity. BF4060, BACCAC_02680, BACCAC_03717 and Amuc_0724 showed an increase in activity against blood group H relative to A and B, suggesting the α1,3 decorations of the A and B oligosaccharides are not well accommodated by these enzymes. In contrast, BT2824, BF4058 and Amuc_2108 activities were all displayed similar activities against blood group A, B and H structures, indicating that the common α1,2-fucose decoration is predominantly causing the reduction in activity relative to LNnT. During prolonged incubation most of the GH16 enzymes had some activity against the milk-derived oligosaccharides with blood group structures and, furthermore, the products from porcine small intestinal mucin included those where the α1,3-GalNAc of blood group A would be in the -4 position plus a variety of fucose and sulfate decoration (Supplementary Fig. 8, *glycans 9, 11, 12*, *14 and 23*).
All nine GH16 family members were tested against a series of disaccharides, but no activity could be observed (Supplementary Fig. 12). This is to be expected with endo-acting enzymes, as more than two subsites have to be occupied to provide the binding energy for catalysis. Activity was possible for most enzymes against Lacto-N-triose, reiterating the requirement for a sugar in the -2 position (Supplementary Fig. 12g). A number substrates were tested that had an α-linked sugar at this position, but no activity was seen (Supplementary Fig. 12). This is most likely due to the α-linkage causing a more kinked chain that cannot be accommodated by the enzyme, similar to when a GalNAc is in the -2 as described above (Supplementary Fig. 11e).

Previous reports of host glycan degradation by members of the GH16 family

Although most of the GH16 family members characterised to date have activity against terrestrial or marine plant polysaccharides, there have been two previous reports of GH16 enzymes with activity against host glycans. One of these was the activity of a GH16 family member from *Sphingobacterium multivorum* against keratan sulfate[22, 23]. This is usually an environmental bacterial species, but can cause human infections, usually in immunocompromised patients[29]. Phylogenetic analysis of this GH16 shows the *S. multivorum* sequence clusters with the *Bacteroides* spp. sequences investigated in this study (Supplemental Figs. 5 and 6, pale orange). This enzyme was found to hydrolyse the

Galβ1,4GlcNAc bond to release predominantly 6-O-sulfo-GlcNAcβ1,3Gal and GlcNAcβ1,3Gal products from a variety of keratan sulfate sources. Interestingly, the *S. multivorum* enzyme was found to also cleave milk oligosaccharides capped with sialic acid, which is different from the activity we observe in the O-glycan GH16 enzymes characterised here.

The second example of a GH16 with activity against O-glycans is from *Clostridium perfringens* and capable of removing the GlcNAcα1,4Gal disaccharide from the non-reducing terminal of the O-glycan[30, 31], which is an epitope associated only with stomach mucin[32, 33]. The crystal structure revealed a pocket-like active site tailored to recognise the GlcNAcα1,4Gal disaccharide substrate[34] (Supplementary Fig. 18b).

Keratanase activity

There are currently three reported classes of enzymes that have endo-activity against keratan - the β-galactosidases described above and keratanases type I and II. Type I enzymes are also β-galactosidases, but require sulfation for activity and cannot hydrolyse unsulfated glycans[23]. Type II enzymes are endo-β-N-acetylglucosaminidases, so hydrolyse a different linkage to the GH16 enzymes, and release disaccharides and tetrasacchairdes with varying amounts of sulfation and also tolerate fucose[35, 36]. There have been other reports of endo-β-galactosidases active on keratan sulfate and milk oligosaccharides from *Citrobacter freundii*, *Coccobacillus spp*. and *B. fragilis*, but insufficient information in the literature prevents us from confirming if these are from the GH16 family[23, 37, 38, 39].

The GH16 enzymes reported here generated products from egg and bovine corneal keratan sulfate visible by TLC and were analysed in the same way as O-glycan products with procainamide labelling and LC-FLD-ESI-MS (Supplementary Fig. 14). The results indicate some sulfate groups can be tolerated by the GH16 family members, but heavily sulfated O-glycan fragments could not be degraded further (for example, *glycans 13-15*). This activity is distinct from Keratanase type I and II activities.

Screening of mucin-associated GH16 enzymes against other polysaccharides

Recombinant forms of the mucin associated GH16 enzymes were tested for activity against a range of β-glucan and β-galactan polysaccharides that have previously been shown to be substrates for GH16 family members. The data revealed little activity for most of the enzymes, except Amuc_0724, which displayed endo-like activity against marine laminarin and weak activity against barley β-glucan and lichenan (Supplementary Fig. 15). The possible structural rationale for this activity is discussed in the structural sections of the Main text and Supplementary Information below. BF4060, BACCAC_02680 and BACCAC_03717 also displayed some very weak activity against laminarin, but not any of the other plant polysaccharides tested. We also tested other host polysaccharides that are likely present in

the gut mucus layer, including the GAGs CS, Hep and HA. No products were observed for CS and HA by TLC, but for Hep there was a single low MW product observed with BT2824, BF4060, BACCAC_03717, Amuc_0724 and Amuc_2108, and two different products for Amuc_0875 (Supplementary Fig. 15n-p). This identity of this product is unknown and could be an artefact, but the linker between heparan sulfate polysaccharides and protein is GlcAβ1,3Galβ1,3Galβ1,4Xyl, which has a potential GH16 cut site. We explored the possibility that the O-glycan active enzymes could cleave this linker, but no activity was observed against the human proteoglycan syndecan (Supplementary Fig. 15q).

Further discussion on the crystal structures of the O-glycan active GH16 enzymes

The characteristics of the sugar product in the BACCAC_02680^{E143Q} structures.

The non-reducing end Gal and the -2 GlcNAc BACCAC_02680^{E143Q} could be modelled in the most stable $^{4}C_{1}$ chair conformation, whereas the reducing end Gal, occupying the -1 subsite, presented a $^{1}S_{3}$ skew boat conformation. The conformation was checked by Privateer[40] (Supplementary Fig. 17, and Supplementary Table 7). The occurrence of a less stable conformation at this position has been described previously as a structural adaptation during hydrolysis in a GH16 1,3-1,4-β-glucanase[41]. The resolution of the ligand in the BF4060 structure was insufficient to define the conformation of the monosaccharides, so the sugar was modelled based on the trisaccharide from the BACCAC_02680^{E143Q} structure.

Accommodation of a glucose or galactose at the -1 subsite in GH16 family members.

At the -1 subsite, the selection of glucose or galactose is very significant in a GH16 structure as the O4 points in towards the binding cleft for both sugar configurations. Analysis of different GH16 structures available, however, reveals no strict rule for how space is created for the equatorial O4 in glucose or tightened up for the axial O4 of galactose. However, the residues on finger 1 always play a key role in controlling this space. In the *Bacteroides* GH16 structures described in this study, a tryptophan from finger 1 blocks off the possibility of an equatorial positioning in this area to produce a galactose-tailored pocket (Fig. 6b and Supplementary Fig. 20a). In contrast, a surface representation of the Amuc_0724 structure shows a relatively open space around the C4 and C6 formed by finger 1 being further away, with an arginine replacing the tryptophan and relatively small residues lining the β-strands in this area (Supplementary Fig. 20a). This is a possible structural rationale for the activity of Amuc_0724 towards glucose polymers.

A closer look at this region in Amuc_0724 structure, shows that this open space is actually a short tunnel and possibly accommodates decoration of the galactose, potentially of the C6 with sulfate as can occur in some mucins (Fig. 6c). Pockets and tunnels above the -1 subsite

have previously been observed in a number of other GH16 structures, for example, the ZgLamC^{E142S} structure from *Zobellia galactanivorans* (Supplementary Fig. 20b). This enzyme is active on laminarin (predominantly β1,3-glucan with occasional β1,6 decorations), but also mixed-linked β1,3/4 glucan. ZgLamC^{E142S} was crystallised with a glycerol inside the -1 subsite pocket and it was suggested that it may be able to accommodate a branching β1,6 glucose decoration at this site[42]. To test this idea out for the O-glycan active GH16 family members, especially Amuc_0724, we took lacto-N-hexaose and enzymatically removed the capping galactose to leave a lactose with two GlcNAc branches linked to the galactose through β1,3 and β1,6 bonds (see Supplementary Fig 25b for glycan structure). Most of the enzymes were active on the same structure with just the β1,3-linked GlcNAc (Supplementary Fig. 12g), however, no significant activity could be seen against the branched substrate. The -1 site in Amuc_0724 (and the other GH16 enzymes) therefore most likely cannot accommodate a β1,6-GlcNAc decoration.

GH16 family members that have to accommodate galactose at the -1 position include the agarases, porphyranases and pectic galactanases. In the agarase structures currently available that are complexed with substrate or product, a glutamic acid from the β-sheet of the cleft H-bonds with and fills the space around the C4 hydroxyl. A proline from finger 1 sits adjacent to this (Supplementary Fig. 19c)[43]. In the porphyranase structures, there is a glutamic acid in approximately the same position, but an arginine from finger 1 also coordinates with C4 and fills this space (Supplementary Fig. 18c)[27]. There are currently no pectic β1,4-galactanase structures to allow comparison. From these observations about the -1 subsite in all GH16 structures, we can conclude that selection of glucose of galactose at the -1 position is achieved in multiple ways by GH16 family members.

Specificity for a β1,3-linkage between the -1 and -2 sugars.

The linkage requirement between monosaccharides in the -1 and -2 subsites is a 1,3 for some GH16 family members (β-glucanases, enzymes targeting marine polysaccharides, and now O-glycanases described here), but this is not the case for xyloglucan hydrolases, xyloglucan endo transferases, pectic galactosidases and chitin β1,6-glucosyltransferases. This specificity is commonly selected for in GH16 β-glucanases through the geometry of the two hydrophobic platforms that the sugars at these positions sit on, which are at 90 ° relative to each other[44]. In the structures available for β-glucan-active enzymes, where a glucose would be in the -2 position, an aromatic residue from finger 3 or the local β-strand acts as a platform for the α-face of this sugar to stack parallel against. The O-glycanase GH16 structures presented in this study also have this aromatic platform, so are most comparable to glucanases in this respect (Fig. 6b). This structural observation corresponds with the biochemical characterisation of the O-glycan active GH16 family members described above as requiring a

β1,3-linked GlcNAc in the -2 subsite for activity. Interactions with the β-face of this sugar is usually through residues coming from finger 1 and from the β-sheet in the cleft, but these are variable even within enzymes with the same activities. The enzymes degrading marine polysaccharides have to deal with an α-linkage at the -2 position, an anhydrogalactose in the case of agarases and carrageenanases, and sulfation at C6 for porphyranases. The structures available for these enzymes reveal the accommodation of these features at the -2 does not involve aromatic stacking but coordination through polar interactions and non-parallel π-stacking with aromatic residues (Supplementary Fig. 18c).

The N-acetyl on the C2 of the GlcNAc (a sugar not found in other GH16 enzyme substrates i.e. unique to mucin) at -2 in the O-glycanase complexes points out into solution away from the cleft, so accommodating this group in a specific pocket is not a requirement for the enzymes reported here. The cleft of the GH16 does have to be fairly open to accommodate the N-acetyl and the group would potentially cause a steric clash in some GH16 enzymes with more closed clefts, so this is likely a contributing specificity determinant for the polyLacNAc O-glycan activity within GH16 enzymes.

In contrast to what has just been discussed, some GH16 enzymes do not require a selection for a 1,3 linkage between the -1 and -2 subsites. Examples of these are the GH16 enzymes with specificities towards xyloglucan (hydrolases and endo-transferases) and chitin β1,6 glucosyltransferases. These substrates have repeating β1,4-linkages, so require enzyme clefts with a more open structure around the C4 in comparison to the β-glucanases and O-glycanase structures. This structural feature is generated by the absence of a Finger 1 in structures that are available. Structures of the xyloglucan active enzymes show a significantly different cleft to accommodate these β1,4-linkages (Supplementary Fig. 18a). There are currently no structures available of chitin β1,6-glucosyltransferases for comparison.

The -3 subsite and beyond.

In this region of the O-glycanase enzyme structures it is clear there is potentially more room for terminal blood group decorations or decorations on the polyLacNAc chains to be accommodated. The biochemical data produced for the O-glycanases confirm the blood group structures are accommodated for some of the enzymes in -3 subsite (Supplementary Figs. 10 and 11f-h). The area around the -3 subsite galactose is fairly open in the BACCAC_02680^{E143Q} and BF4060 structures and the same is true when the trisaccharide product is overlaid into BACCAC_03717 structure. The extended Finger 2 in Amuc_0724, however, could theoretically interact with a longer substrate. An extensive loop reaching down to the binding cleft has been seen before in other GH16 structures, such as the κ-Carrageenases from *Zobellia galactanivorans* and *Pseudoalteromonas carrageenovora* (Supplementary Fig. 18d),

which possess a similar Finger 2 that interacts with the -4 sugar in the case of the *P. carrageenovora* structure[45].

In the O-glycanase structures presented here, there is space at the -3 for a β1,4-linked galactose, but it is easy to see that if the sugar in the -3 did not continue the chain in a linear fashion then the Gal may not be accommodated (Fig. 6b). We were able to see this when the nine O-glycan active GH16 enzymes were tested against Galβ1,4GalNAcβ1,3Galβ1,4Glc and saw no activity, except for Amuc_0875 that displayed trace activity (Supplementary Fig. 11). The axial position of the hydroxyl at C4 in a GalNAc would mean the galactose in the -3 would now be kinked, thereby showing specificity for a GlcNAc at the -2 position of the O-glycanases. GH16 enzymes that cleave agarose, carrageenan and porphyran all have to accommodate Gal in their -2 sites, but these polysaccharides are fairly linear however as they are composed of alternating α1,3 and β1,4 linkages (Fig. 6e).

The positive subsites

In the GH16 O-glycanase structures described here, only the negative subsites are occupied with sugar. The positive subsites of retaining GH structures are rarely occupied with product due to the low binding affinity of the positive subsites to facilitate rapid leaving of the product after the initial glycosylation step[46, 47]. However, structures of a GH16 laminarinase from *Phanerochaete chrysosporium* with different glucan products bound to the positive subsites of the enzyme allow us to explore how sugars in the potential positive subsites of the O-glycan-active GH16 enzymes could be accommodated. Orientation of the glucose in the +1 subsite of three structures of the laminarinase (PDB codes 2W52, 2WLQ and 2WNE) has the β-face of the sugar stacking against a tryptophan from finger 6. The O-glycan active enzymes have an aromatic residue that overlays well with this tryptophan and analysis of the potential positive subsites of GH16 structures show that the majority of them have an aromatic in this area that could comprise the +1 subsite (Fig 6b, W250 in BACCAC_02680 and W252 in BF40470). Aromatic residues are sometimes not present in the +1 subsites of GH16 enzymes, such as those active on marine polysaccharides.

The position of the +1 sugar in the *P. chrysosporium* GH16 suggests the hydrolysis of a β1,3 bond, consistent with laminarin being composed entirely of β1,3 linkages. For the O-glycan active GH16 enzymes, however, this would be a β1,4 bond and rotation of the sugar from the laminarinase to reflect this would place the C6 pointing into the cleft of the O-glycanases. An overlay of the +1 glucose (from 2WLQ) indicates that this would be spatially difficult to accommodate, but it is possible the sugar sits slightly further away from the cleft than seen in glucanases (Fig. 6d).

The importance of the positive subsite residues was analysed by comparing the rates against TriLacNAc versus milk derived oligosaccharides (LNnT and LNT), which have either GlcNAc

or Glc at the +1, respectively, and are different lengths (Supplementary Fig. 10). The activity of BF4060 (and also its close homologue BACCAC_02680) on the milk oligosaccharides was significantly decreased compared to TriLacNAc, but this was not the case for BACCAC_03717, suggesting the former enzyme could have a preference for GlcNAc at +1. The structures of BACCAC_03717 is much more open at this position, whereas BF4060 (and BACCAC_02680) has a much more enclosed region for the +1 sugar to sit in and a serine (S174) coming from Finger 5 could potentially interact with the N-acetyl and pincer this group against Finger 6 (Fig. 6d). The openness of the Amuc_0724 structure in the potential positive subsites and substrate depletion data for this enzyme support this idea. However, it is also possible that the enzymes are sensitive to the number of positive subsites that are filled by substrate, as TriLacNAc is a larger substrate (dp 6) than either of the milk derived tetrasaccharides.

For the Amuc_0724 structure, the tryptophan in the positive subsites between the potential +1 and +2 sites (W279) had dual occupancy so could be modelled to sit in the cleft or flipped out of this position away from the cleft. The flipped out position of the tyrosines from the two molecules in the unit cell may be an artefact of the crystallographic dimer rather than biologically relevant (Supplementary Fig. 20c), but it is worth noting that a dynamic tyrosine has also been seen in the positive subsites of a GH16 β-glucanase from *B. ovatus*[48] (Supplementary Fig. 20d).

Insights into accommodation of sulfate, fucose and blood group decorations along the polyLacNAc chain by the GH16 O-glycanases.

Mucin O-glycans can have 3S (capping) and 6S decoration on galactose and 6S also on GlcNAc. Overlay of the product from the 5OCQ κ-carrageenases from *Pseudoalteromonas carrageenovora* with the Amuc_0724 structure shows how the tunnel structure in the -1 subsite of the *Akkermansia* enzyme could accommodate a sulfate group on the Gal at 6S, however this is most likely not possible for the *Bacteroides* O-glycanases (Supplementary Fig. 20e)[45]. Overlay of 3ILF porphyranase from *Zobellia galactanivorans* with the O-glycan active GH16 structures mimics a 6S sulfation of the GlcNAc in the -2 subsite and all the enzymes could potentially accommodate this decoration (Supplementary Fig. 20f)[27]. At the -3 subsite, C6 of the galactose points into solution in all the O-glycanase structures, suggesting a sulfate could be accommodated at this site.

Fucose can be linked through α1,2/3/4 bonds in O-glycans. It appears highly unlikely any of the O-glycanases could accommodate a fucose attached to a Gal at -1. However, C3 of the GlcNAc in the -2 position in all the O-glycan active enzyme structures looks open to fucose decoration. In blood group sugars the Gal at -3 has an α1,2-fucose attached. The α1,2-linked fucose would point into the cleft and the α1,3-linked GalNAc or Gal on group A and B,

respectively, would be at approximately 90° to the rest of the linear substrate. As a general trend, the blood group A and B sugars reduce activity of the O-glycan active GH16 enzymes and the removal of the α1,3-linked GalNAc or Gal attenuates this effect (Supplementary Fig. 10). Notably, there does appear to be space that could accommodate an α1,2-linked fucose in a potential -3' subsite in the crystal structures presented in this study.

Minimum substrate requirements for the GH16 O-glycanases.

For all the O-glycanase structures, there are three well-defined subsites from -2 to +1 with some possible interactions at potential -3 and +2 positions. In contrast, it is not unusual for other GH16 family members to have longer binding clefts, which generates a tighter substrate specificity and requirement for the subsites to be filled for catalytic activity; for example, AgaD, which requires a minimum substrate size of DP8[49]. The relatively small number of defined subsites in the O-glycan active GH16 members described in this study and their minimum substrate length being DP3 likely reflects the variable nature of O-glycan as a substrate, particularly in terms of the sulfate and fucose decorations.

Activities of the two other CAZymes from the B. fragilis GH16 PUL

The *B. fragilis* PUL containing the genes encoding the GH16 O-glycanases, also contains genes encoding two other predicted CAZymes from families GH20 (BF4059) and GH35 (BF4061). Both of these enzymes have predicted type I signal peptides (Fig. 1 and Supplementary Table 1). Purified recombinant versions of the GH20 and GH35 enzymes were tested against a variety of defined oligosaccharides to determine their specificity (Supplementary Figs. 25 and 26).

BF4061^{GH35} was found to be active against LacNAc, Lacto-N-biose and Galβ1,3Glc, partially active against lactose and inactive against Galβ1,4Gal (Supplementary Fig. 25). These data indicate the enzyme is a β1,3/4-galactosidase with a preference for GlcNAc>Glc>Gal in the +1 position and thus a specificity for mucin-type oligosaccharides over milk and plant-derived glycans, which do not contain GlcNAc at this position. BF4061^{GH35} was not active against fucosylated versions of these oligosaccharides, indicating that fucose needs to be removed to allow the galactosidase access (Supplementary Fig. 25b). This specificity has been seen previously for the *B. thetaiotaomicron* β1,4-galactosidase (BT0461^{GH2}) against host glycans, where fucose needed to be removed from the antenna of complex N-glycans from human IgA colostrum before the enzyme could act[26]. Interestingly, BF4061^{GH35} described here can accommodate both β1,3 and β1,4-linkages, whereas BT0461^{GH2} can only accommodate β1,4-linkages, reflecting the linkages present in their respective substrates (mucins for BF4061^{GH35} vs complex N-glycans for BT0461^{GH2}). BF4061^{GH35} can also remove β1,3-galactose when there is a GalNAc in the +1 subsite (Supplementary Fig. 26b) and both capping galactoses

from lacto-N-hexaose and these structures are found in O-glycan core and branched structures, respectively (Supplementary Fig. 25).

The second CAZyme, BF4059^{GH20}, is broad acting β-hexosaminidase as it removes both GlcNAc and GalNAc and accommodates a variety of linkages and monosaccharides in the +1 position (Supplementary Fig 26). β1,4-linked chitooligosaccharides of a degree of polymerisation between 2 and 5 could be degraded to GlcNAc and non-reducing end GlcNAc could be removed from O-glycan-type structures (GlcNAcβ1,3Gal, Lacto-N-triose and Lacto-N-hexaose). BF4059^{GH20} can also degrade GalNAcβ1,3Gal, but not GalNAcβ1,3Galβ1,4Glc. The complex N-glycan derived GlcNAcβ1,2Man could also be degraded, further demonstrating the broad activity of this enzyme.

The predicted localisation of these enzymes and their specificities most likely places them in the periplasm of *B. fragilis* acting on the GH16 products that have been imported into the cell. The action of these enzymes plus fucosidases and sulfatases would enable degradation of the GH16 oligosaccharide products down to monosaccharides.

Supplementary Figures

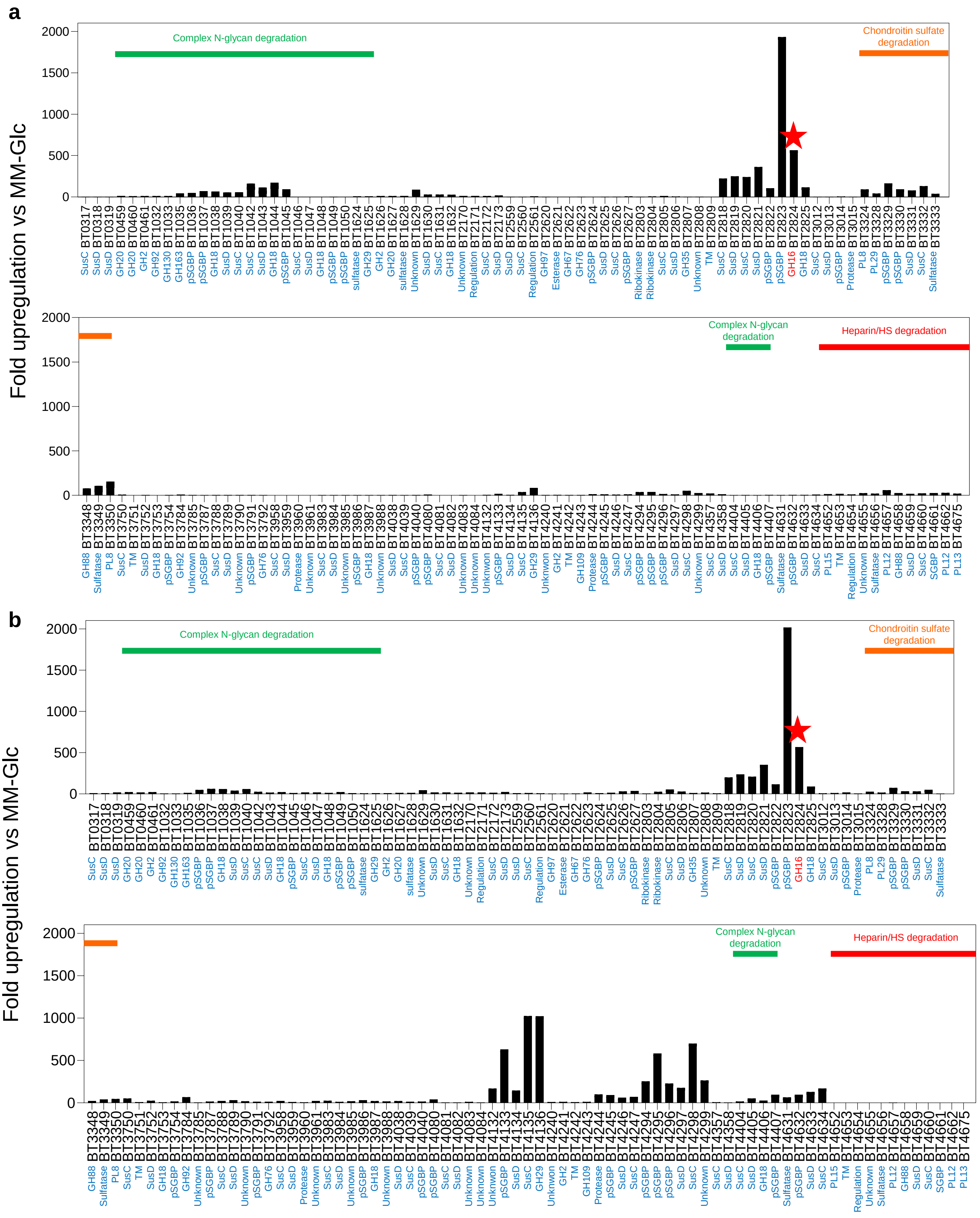

Supplementary Fig. 1 Upregulation of *Bacteroides thetaiotaomicron* genes on mucin O-glycans. Genes upregulated at least 10-fold *in vitro* using purified mucin O-glycans from PGM type III (Sigma) as the carbon source relative to glucose-grown cells (Martens *et al.* 2008) **a**, early in the growth curve. **b**, late in the growth curve. A number of PULs and non-PUL CAZyme clusters were upregulated, including those involved in complex N-glycan and glycosaminoglycan degradation (Briliute *et al.* 2019; Martens *et al.* 2008) . BT2824 GH16 is indicated by a red star.

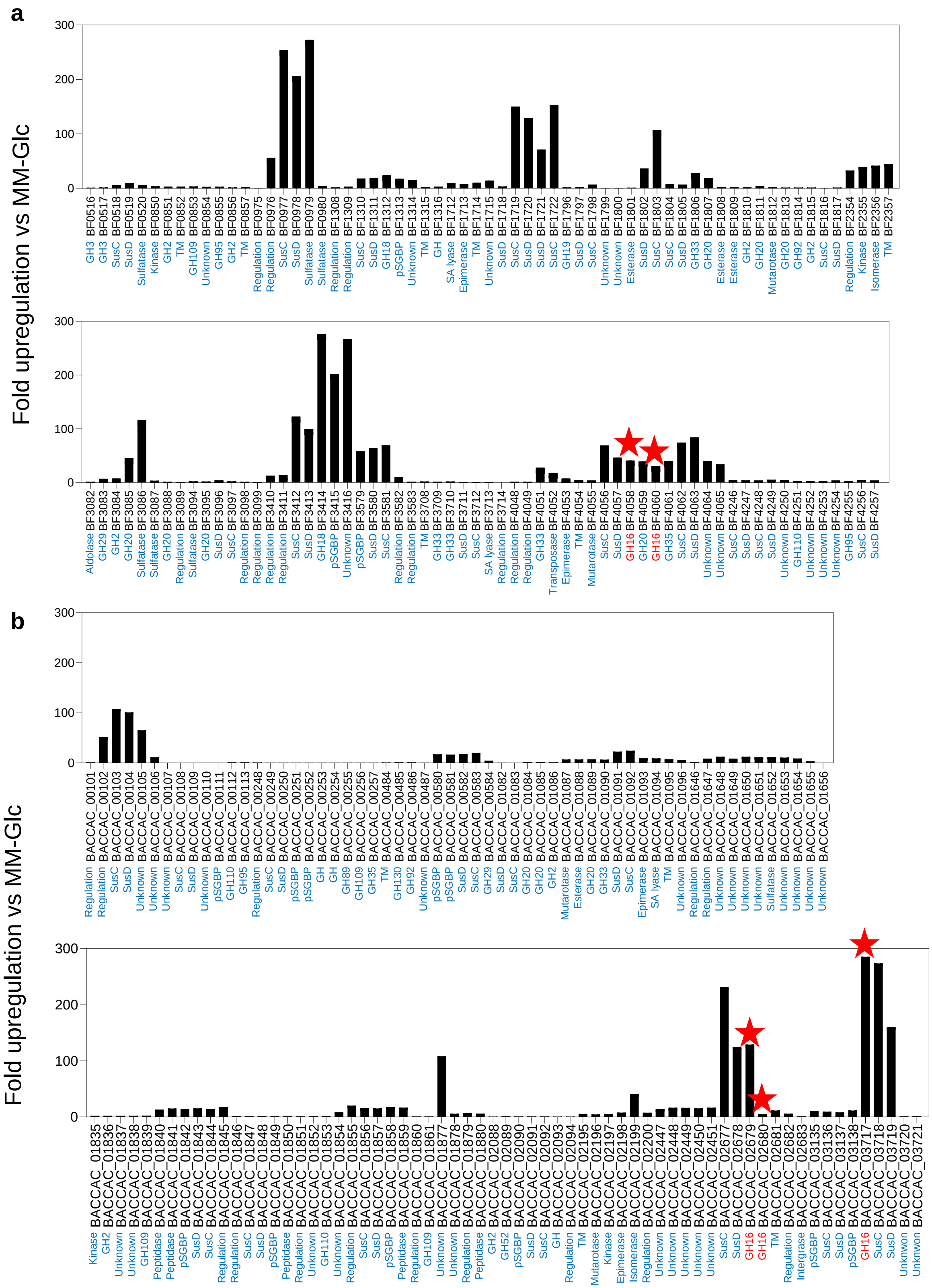

Supplementary Fig. 2 Upregulation of *B. fragilis* and *B. caccae* genes on mucin O-glycans. Genes upregulated at least 10-fold *in vitro* using purified mucin O-glycans from PGM III (Sigma) as the carbon source relative to glucose-grown cells **a**, *B. fragilis* (Pudlo *et al.* 2015). **b**, *B. caccae* (Desai *et al.* 2016). The GH16 family members upregulated are indicated by a red star.

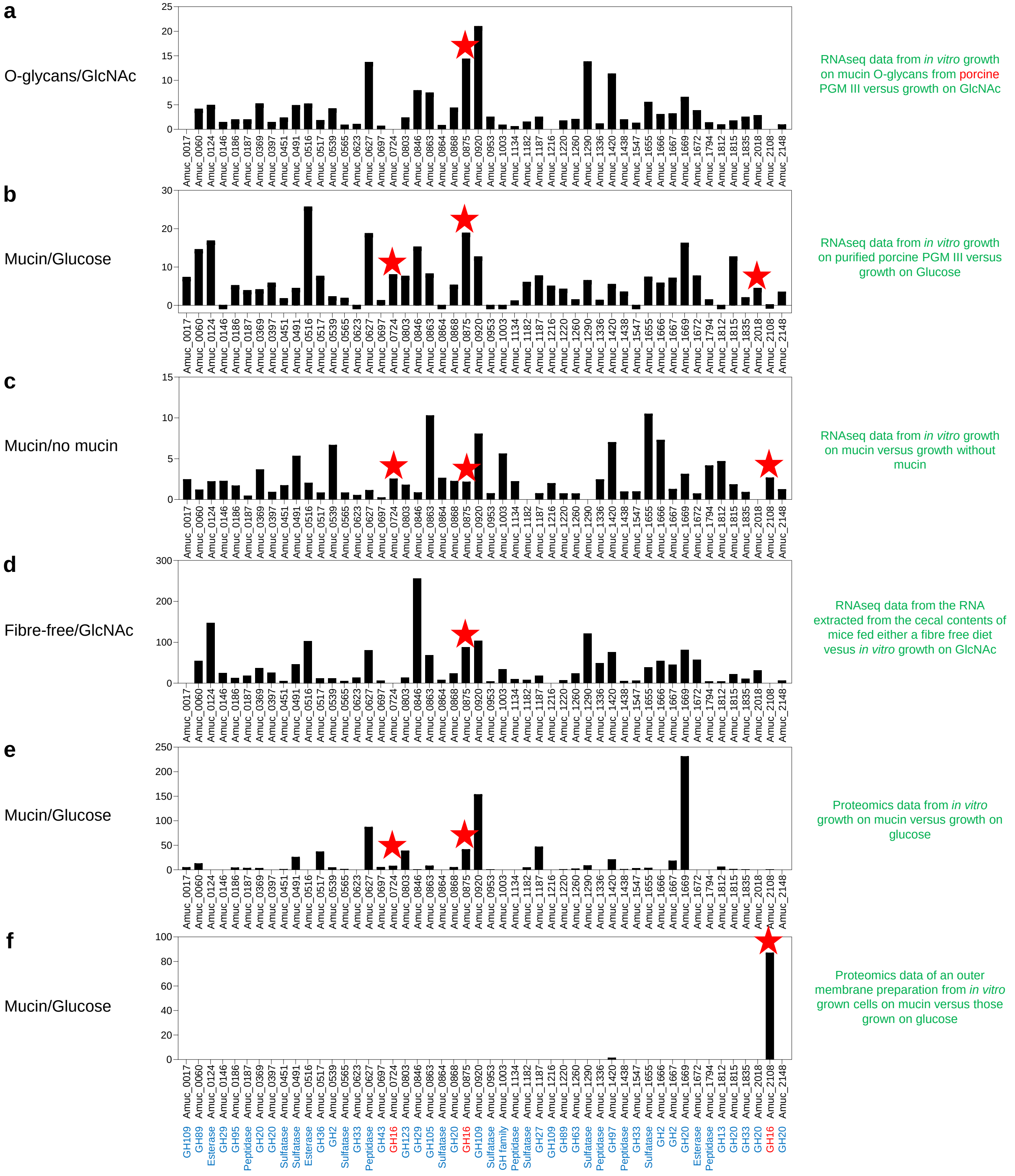

Supplementary Fig. 3. *A. muciniphila* transcriptomic and proteomic data during growth on mucins *in vitro* and *in vivo*. **a**, Genes that are upregulated *in vitro* using purified mucin O-glycans as the carbon source relative to a culture grown on GlcNAc (Desai *et al.* 2016). **b**, Genes that are upregulated *in vitro* using purified porcine gastric mucin Type III (Sigma) as the carbon source relative to a culture grown on GlcNAc (Ottman *et al.* 2017). **c**, Genes that are upregulated *in vitro* using purified mucin O-glycans as the carbon source relative to a culture grown on GlcNAc (Shin *et al.* 2019). **d**, Genes that are upregulated when a mouse is fed a fibre-free diet compared to growth *in vitro* on GlcNAc (Desai et al. 2016). **e**, Relative protein levels during *in vitro* growth on purified PGM III compared to growth on glucose (Ottman *et al.* 2016). **f**, Relative protein levels in the outer membrane fraction during growth on purified PGM III compared to growth on glucose (Ottman *et al.* 2016). It should be noted that the *in vitro* growths a-c were performed in different basal medias. The GH16 family members are indicated by a red star.

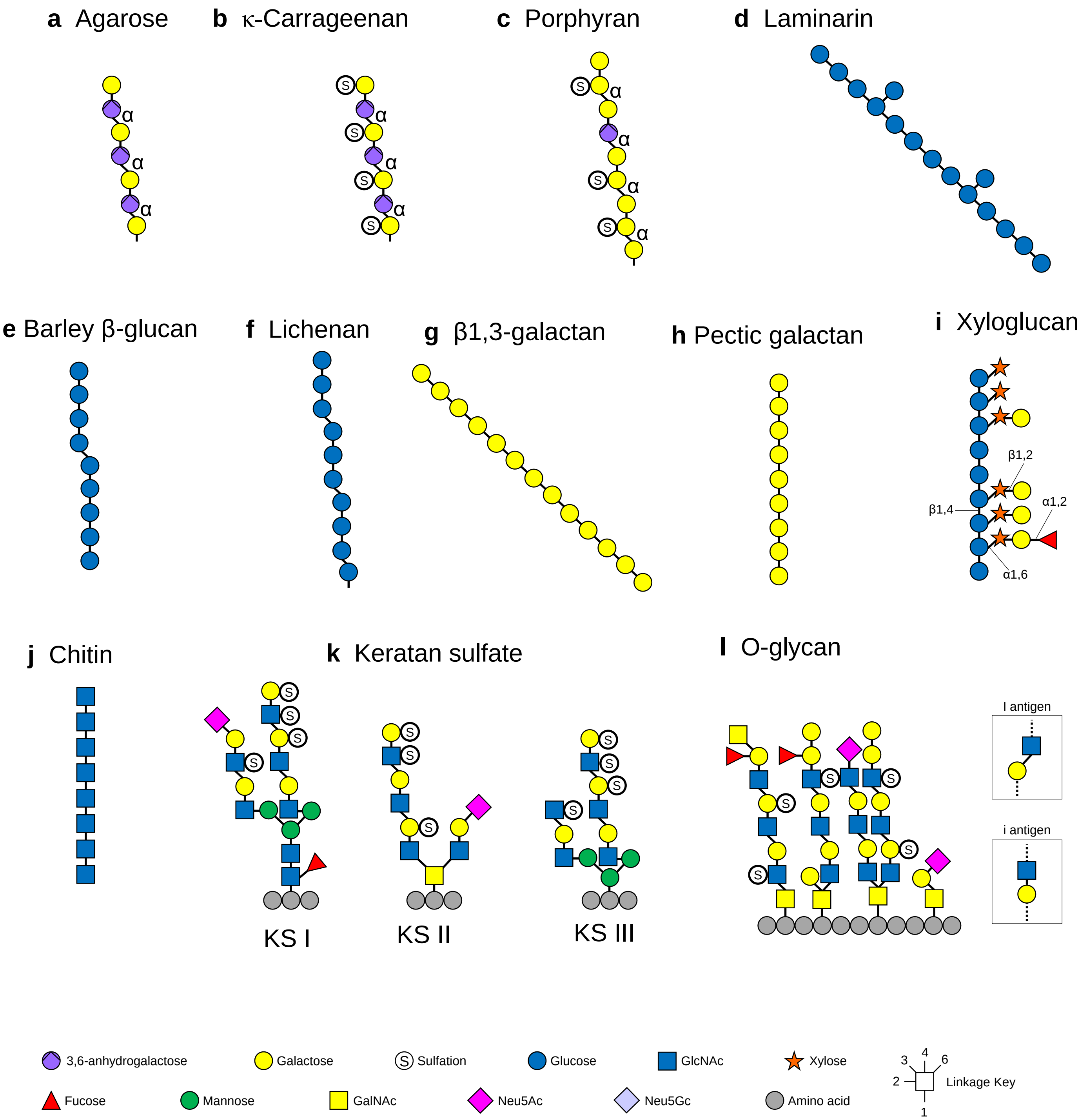

Supplementary Fig. 4 Classes of glycan targeted by GH16 enzymes. Previously characterised GH16 family members target a range of β-linked glucose and galactose polysaccharides from terrestrial and marine plant sources. **a**, Agarose (α1,3-3,6-anhydrogalactose-β1,4-galactose repeating units). **b**, κ-carrageenan (α1,3-3,6-anhydrogalactose-β1,4-galactose6S repeating units). **c**, Porphyran (α1,4-galactose6S-β1,3-galactose repeating units where the galactose6S is sometimes replaced with 3,6-anhydrogalactose). **d**, Laminarin (β1,4-glucan with occasional β1,6-glucose decoration). **e**, Barley β-glucan (β1,4-glucan with occasional β1,3-linkages). **f**, Lichenan (predominantly β1,4-glucan with ~25 % β1,3-linkages). **g**, β1,3-galactan (arabinogalactan backbone; β1,3-linkages). **h**, Pectic galactan (β1,4-linkages). **i**, Xyloglucan (linkages displayed do not follow the key, but are labelled). **j**, Chitin (β1,4-linkages). **k**, Keratan sulfate (polyLacNAc structures that can be O- or N-linked to protein, 6S decoration possible on galactose and GlcNAc and occasional sialylation and fucosylation). **l**, Mucin O-glycans. In keratan sulfate and O-glycan structures the GlcNAc sugars can also be linked through β1,4 and β1,6 linkages (boxes).

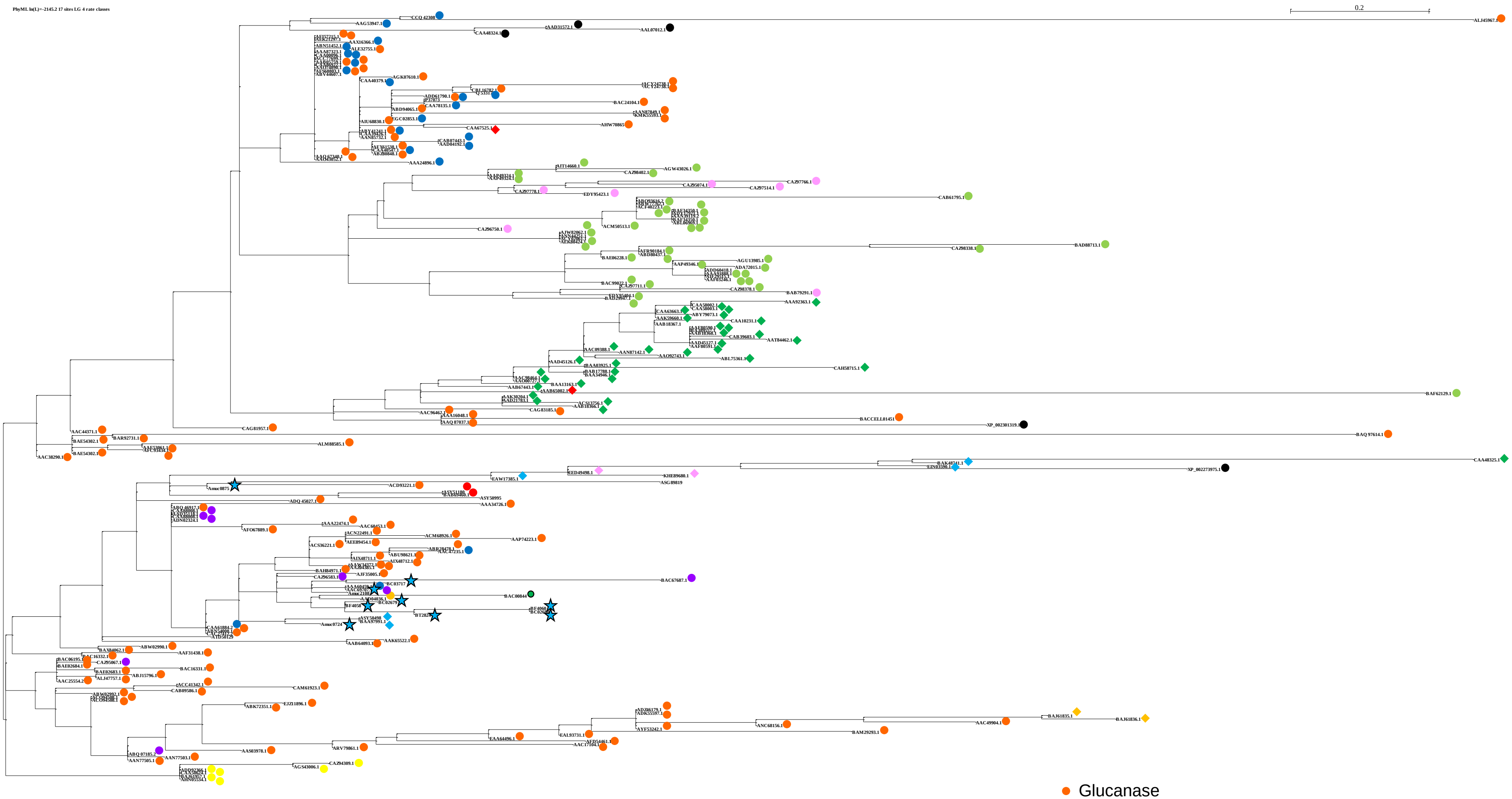

Glucanase
Lichanase
Xyloglucanase
Xyloglucan endotransferase
Agarase
Porphyranase
K-Carrageenase
GlcNAc-a1,4-Gal releasing β-galactosidase
Endo-β1,3-galactanase
Keratan sulfate
Laminarinase
Exo-β1,3-galactanase
Chitin β-glucanosyltransferase
Hyaluronidase
O-glycanases (endo-β1,4-galactosidase)

Supplementary Figure 5│Phylogenetic tree of characterised GH16 family members. The sequences of GH16 family members with reported activities (CAZy database) and the O-glycan active family members characterised in this report (blue stars) were compared as described in Methods. Each CAZy database entry was given a number to simplify the tree. Different specificities can be seen branching off together in many instances. The enzymes are represented by their accession numbers or locus tags. The only exception is the replacement of ‘BACCAC_’ with ‘BC’. The bacterial strains used in this study are listed in Supplementary Table 3.

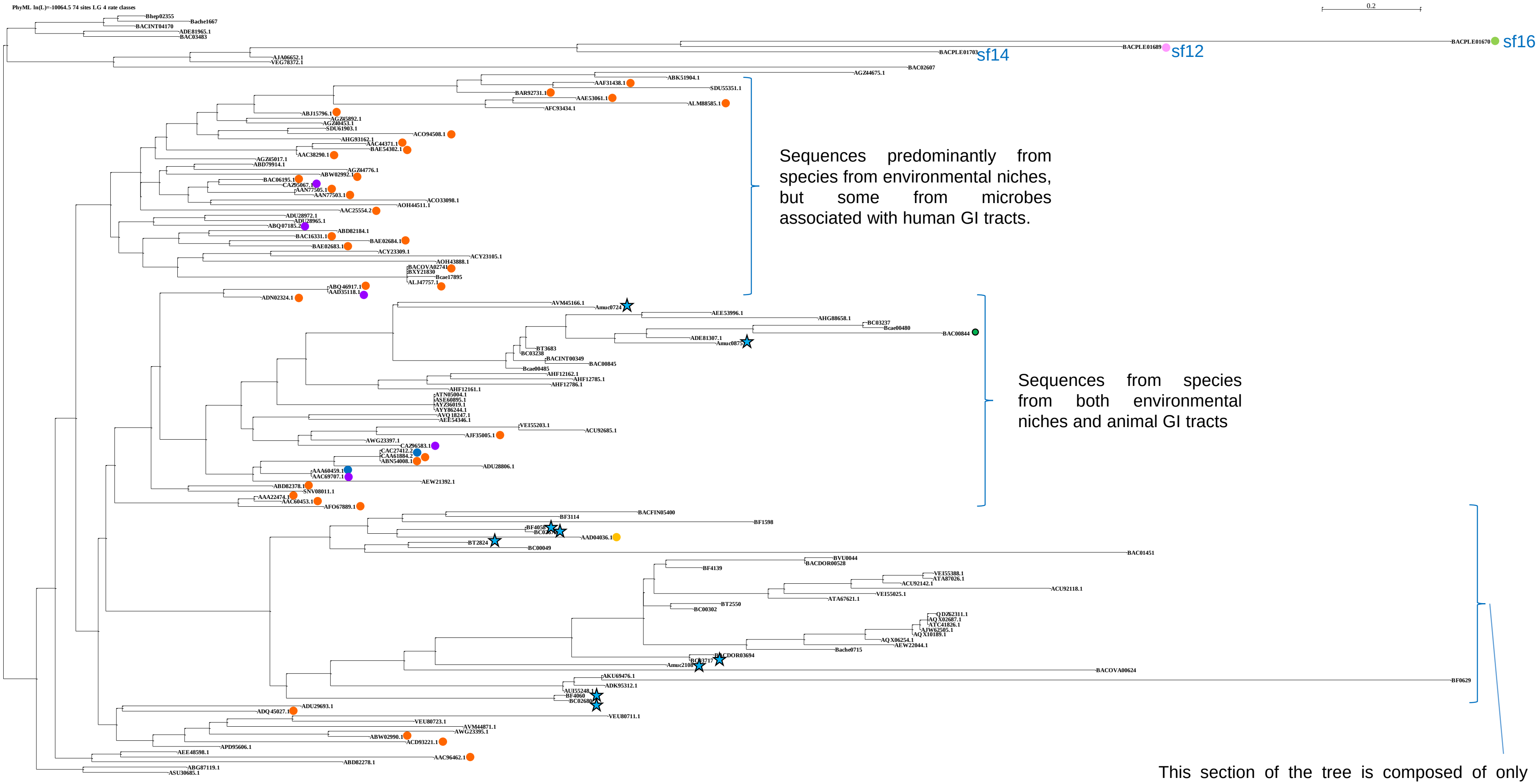

This section of the tree is composed of only sequences originating from microbes found in GI tracts of humans and animals. They include mutualists, pathobionts and pathogens (e.g. *Bacteroides* spp., *Capnocytophagia* spp., *Elizabethkingia* spp., *Prevotella* spp., and *Tannerella forsythia*).

Supplementary Figure 6│Phylogenetic tree of a selection of GH16 subfamily 3 members Characterised GH16 subfamily 3 members were analysed alongside a selection of non-characterised subfamily 3 members from microbes with different types of habitats and abilities to be pathogenic to humans. These details are annotated on the tree. GH16 family sequences from *Bacteroides* spp. were also included from subfamilies (sf) 12, 14 and 16 for comparison (see agarase and porphyranase activities, for example (Heheman *et al.* 2012). The bacterial strains used in this study are listed in Supplementary Table 3.

- Glucanase
- Lichanase
- Agarase
- Porphyranase
- Keratan sulfate endo-β-galactanase
- Laminarinase
- Arabinogalactan endo-β1,3-galactanase
- O-glycanase

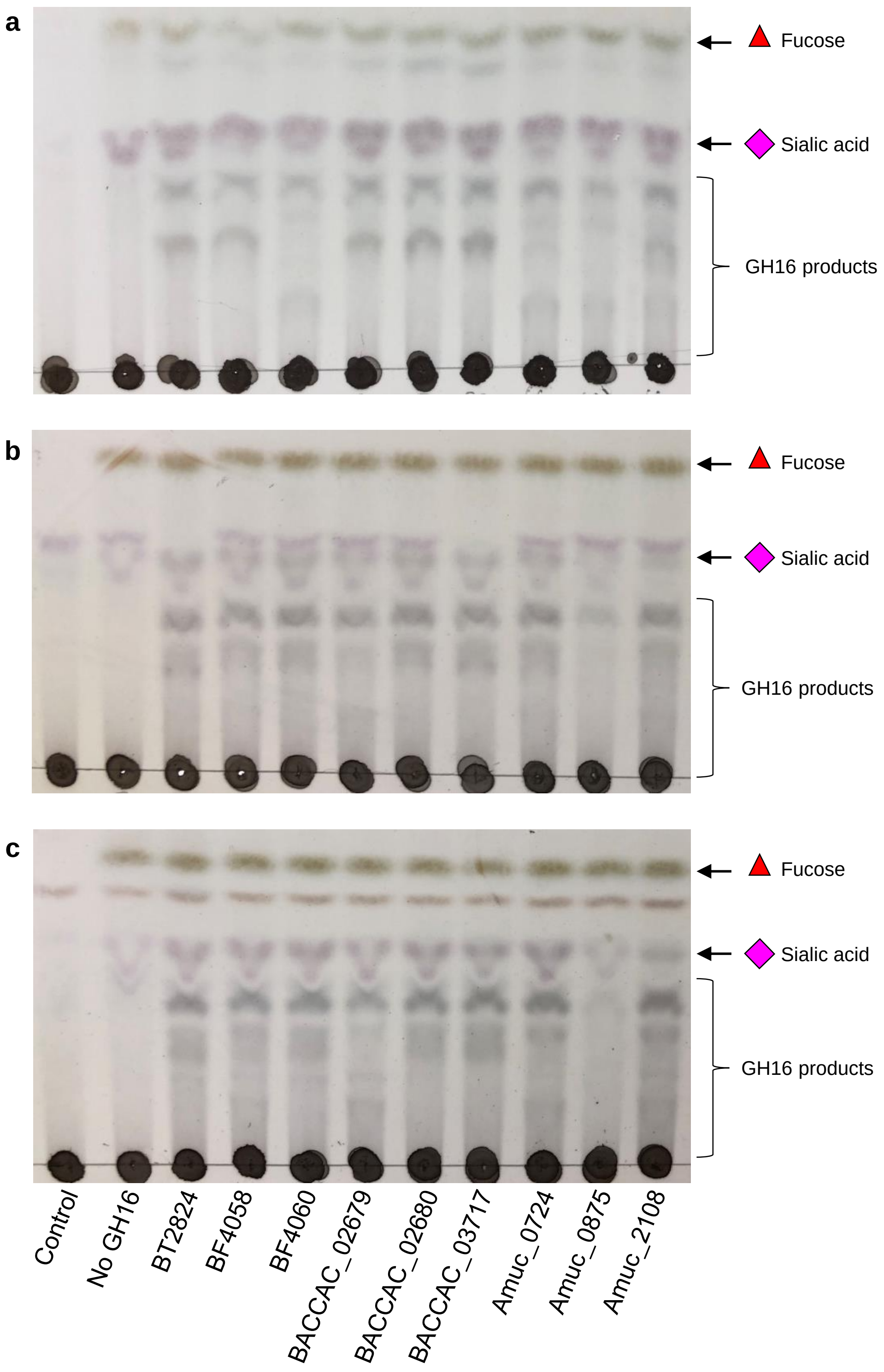

Supplementary Fig. 7 Activity of the GH16 enzymes against porcine small intestinal mucin and commercially available porcine gastric mucin (PGM). a, Porcine small intestinal mucin. **b**, PGM type II. **c**, PGM type III. All assays with a GH16 enzyme also included a sialidase and α1,2-fucosidase, BT0455^{GH33} and a GH95 (from *Bifidobacterium bifidum*; see Supplementary Fig. 24), respectively. Control lane is substrate only and No GH16 lane is mucin + sialidase and fucosidase only. The results are representative of two independent replicates. Source data are provided in the source data file.

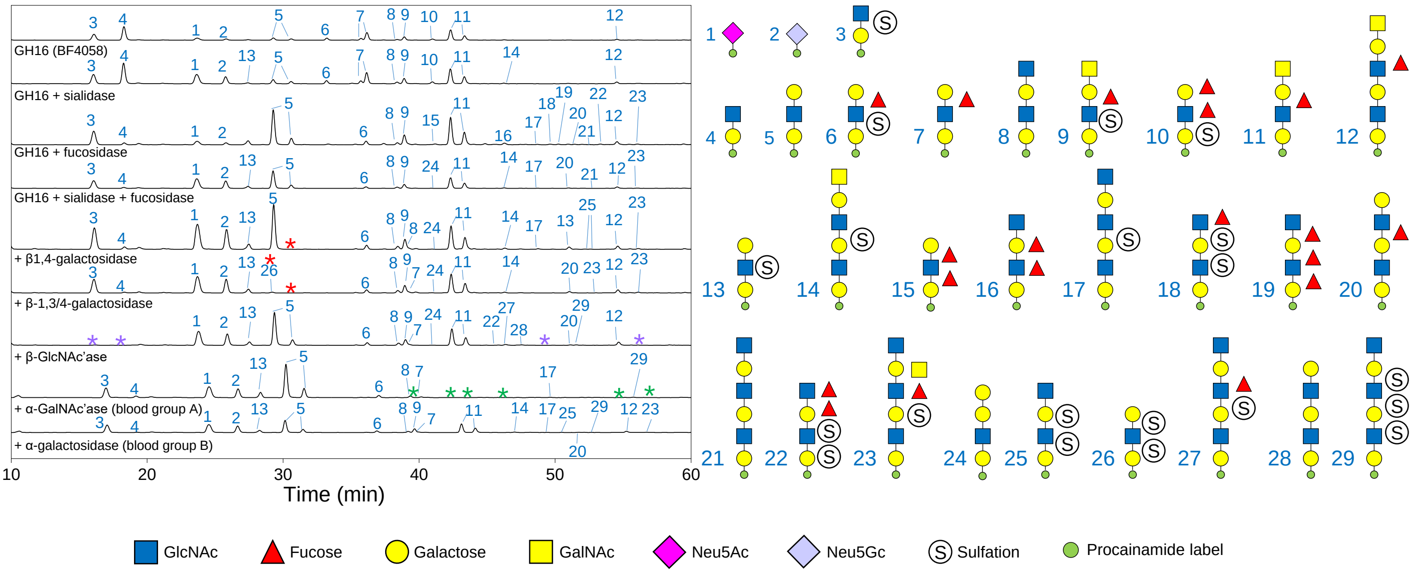

Supplementary Fig. 8 Exo-glycosidase treatment of GH16 O-glycanase products. A series of exo-acting enzymes were incubated in different combinations with BF4058 to provide further insight into the oligosaccharide products released by the GH16. Labels indicating which enzymes were used in the assays are shown below each chromatogram. Red, purple, and green asterisks highlight the peaks of interest for the β-galactosidases, the β-GlcNAc'ase, and the α-GalNAc'ase, respectively. *Glycan 6* shifts in position and these two different resolving times indicate different isomers - for instance this could simply mean a different linkage between two sugars or could be a completely different re-ordering of the monosaccharides within a glycan. See Supplementary Information for full description.

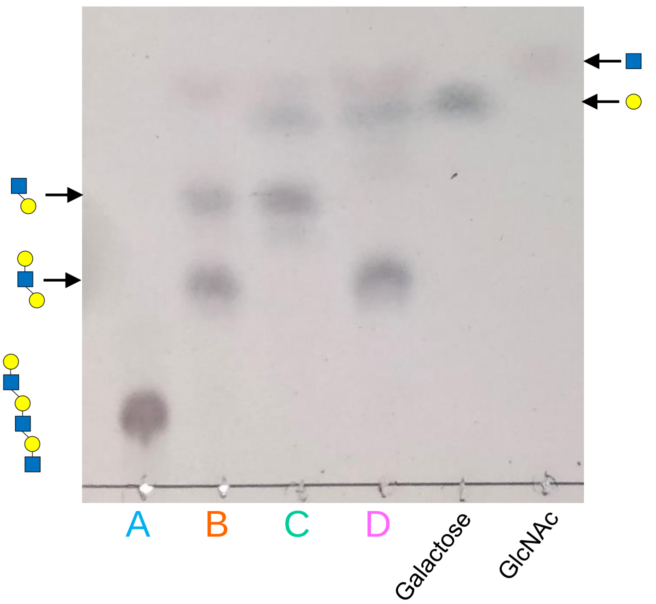

A. Control
B. Amuc_0724
C. Amuc_0724 post-treated with β1,4-galactosidase (BT0461^{GH2})
D. Amuc_0724 post-treated with broad-acting GlcNAc’ase (BT0459^{GH20})

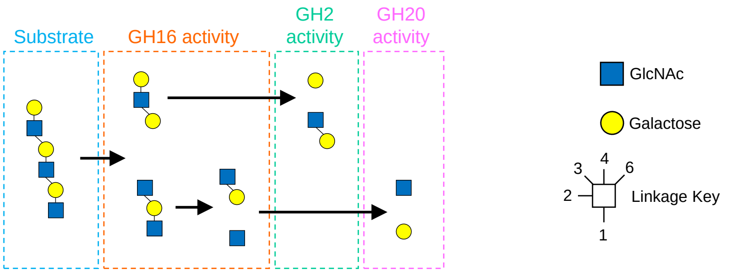

Supplementary Fig. 9 Activity of Amuc_0724 GH16 against TriLacNAc. The products of TriLacNAc digestion by Amuc_0724 (lane B) were incubated with either a GH2 β1,4-galactosidase (lane C) or a GH20 β-GlcNAc’ase (lane D) to confirm their identity. The results are representative of two independent replicates. Source data are provided in the source data file.

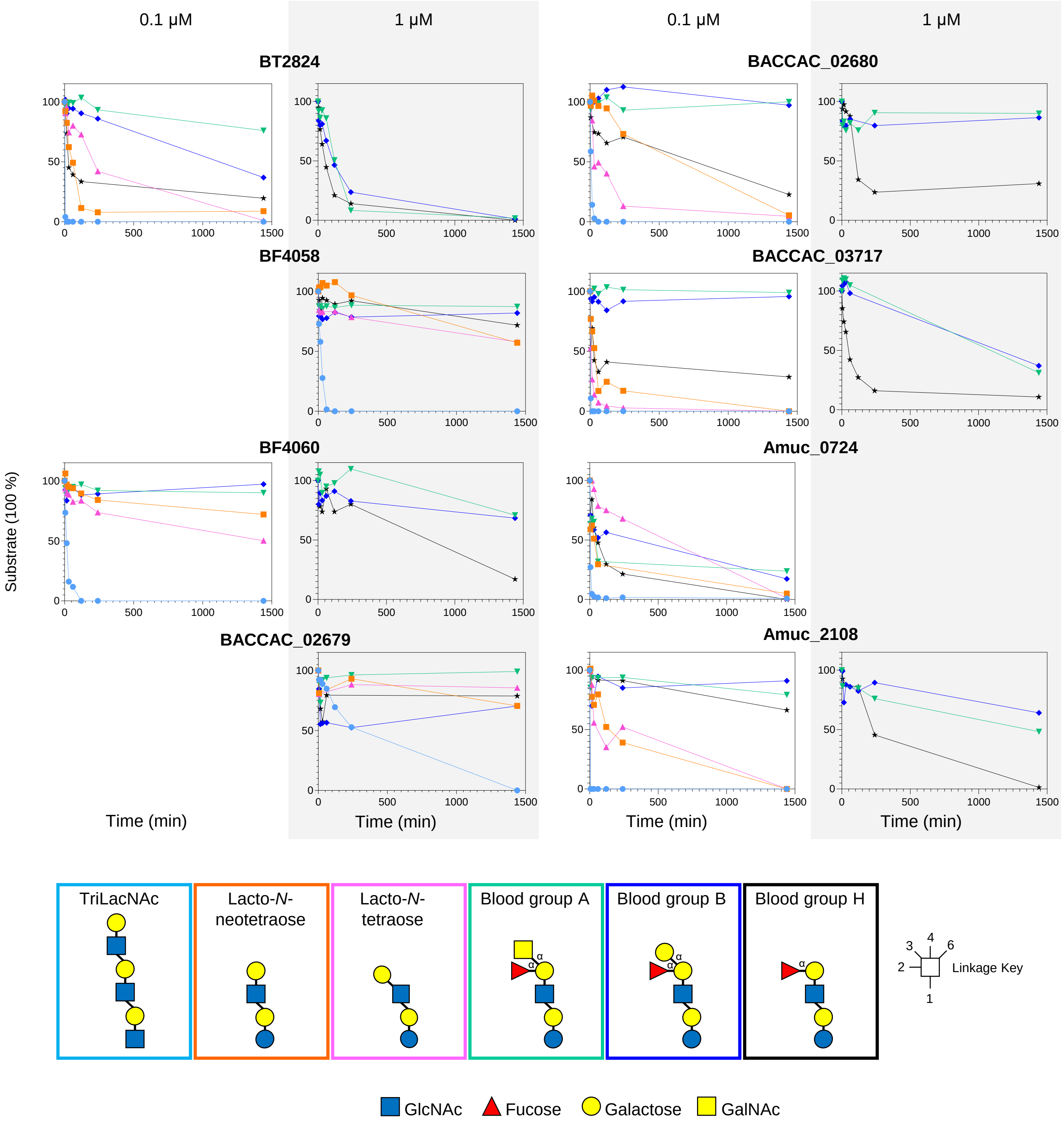

Supplementary Fig. 10 Substrate depletion assays with defined oligosaccharides to probe the importance of the different sub-sites of the GH16 enzymes Assays were carried out with 1 mM substrate concentration, 20 mM MOPS, pH 7.0 at 37 °C and samples taken at different time points to monitor substrate depletion by HPAEC-PAD. Enzyme concentration was 0.1 µM (white columns) or 1 µM (grey columns). The defined oligosaccharides used were TriLacNAc (light blue circles), Lacto-*N*-neotetraose (orange squares), Lacto-*N*-teraose (pink triangles), blood group A (green inverted triangles), blood group B (dark blue diamonds) and blood group H (black stars). These assays were carried out once, but pilot experiments were run to decide on the parameters and these data was consistent with the final data.

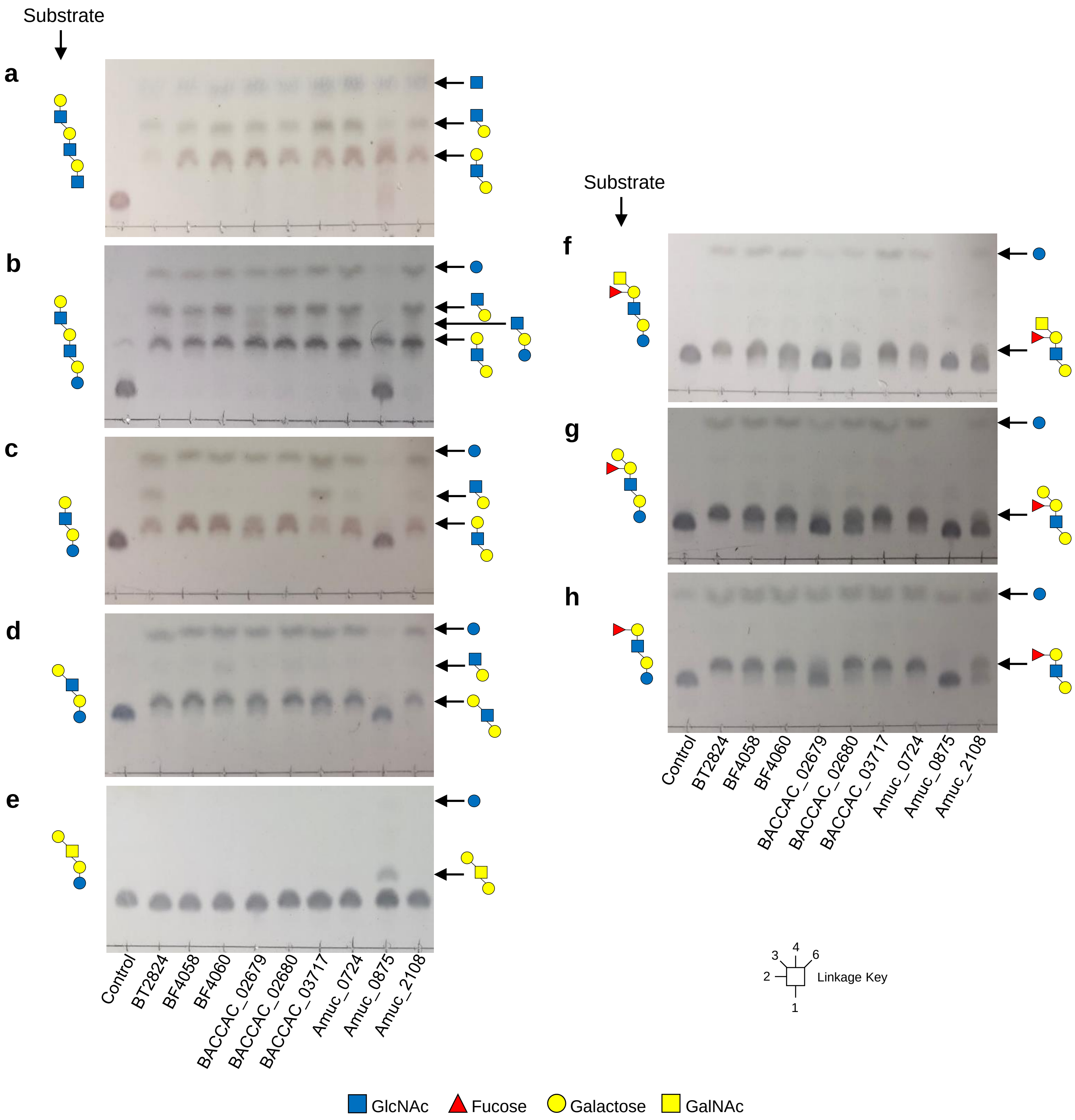

Supplementary Fig. 11 Activity of the GH16 enzymes against oligosaccharides. Products of GH16 activity were visualised by TLC. **A,** TriLacNAc. **b**, paraLacto-N-neohexaose. **c**, Lacto-N-neotetraose. **d**, Lacto-N-tetraose. **e**, Galβ1,3GalNAc β1,3Galβ1,4Glc. **f**, Blood group A hexasaccharide II. **g**, Blood group B hexasaccharide II. **h**, Blood group H pentasaccharide generated from A or B. Control = no enzyme added. Assay were 1 mM substrate, 3 µM enzyme, 20 mM MOPS, pH 7.0 at 37 °C overnight. 3 µl of the assay was spotted on to the plate before running. The results are representative of two independent replicates. Source data are provided in the source data file.

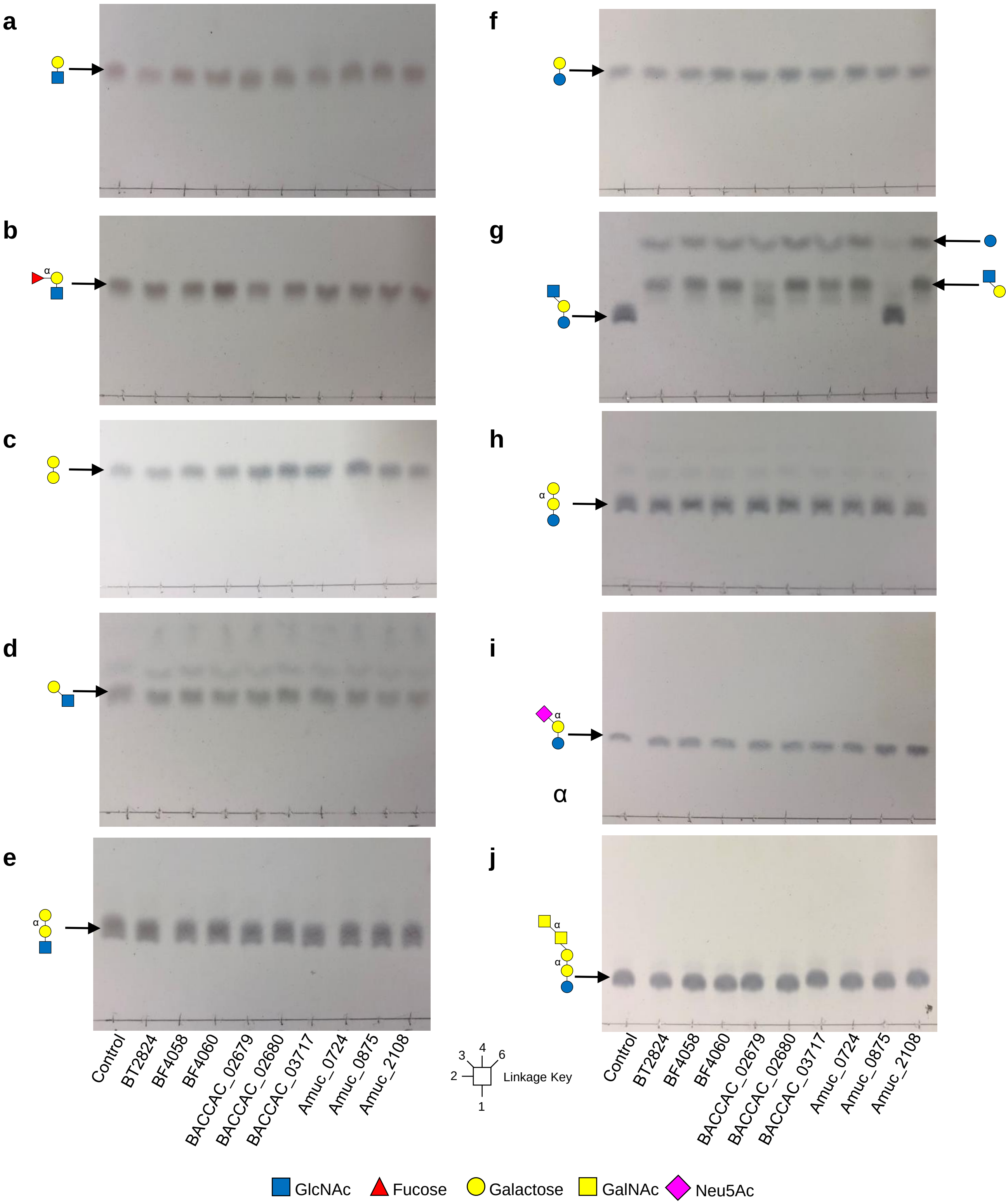

Supplementary Fig. 12 Activity of the GH16 enzymes di- and oligo-saccharides. Products of GH16 activity were visualised by TLC. **a**, LacNAc. **b**, Blood group H trisaccharide II. **c**, Galβ1,4Gal. **d**, Lacto-N-biose. **e**, P1 antigen. **f**, Lactose. **g**, Lacto-N-triose. **h**, Globotriose. **i**, 3-Sialyllactose. **j**, Forssman antigen. Assay were 1 mM substrate, 3 μM enzyme, 20 mM MOPS, pH 7.0 at 37 °C overnight. 3 μl of the assay was spotted on to the plate before running. The results are representative of two independent replicates. Source data are provided in the source data file.

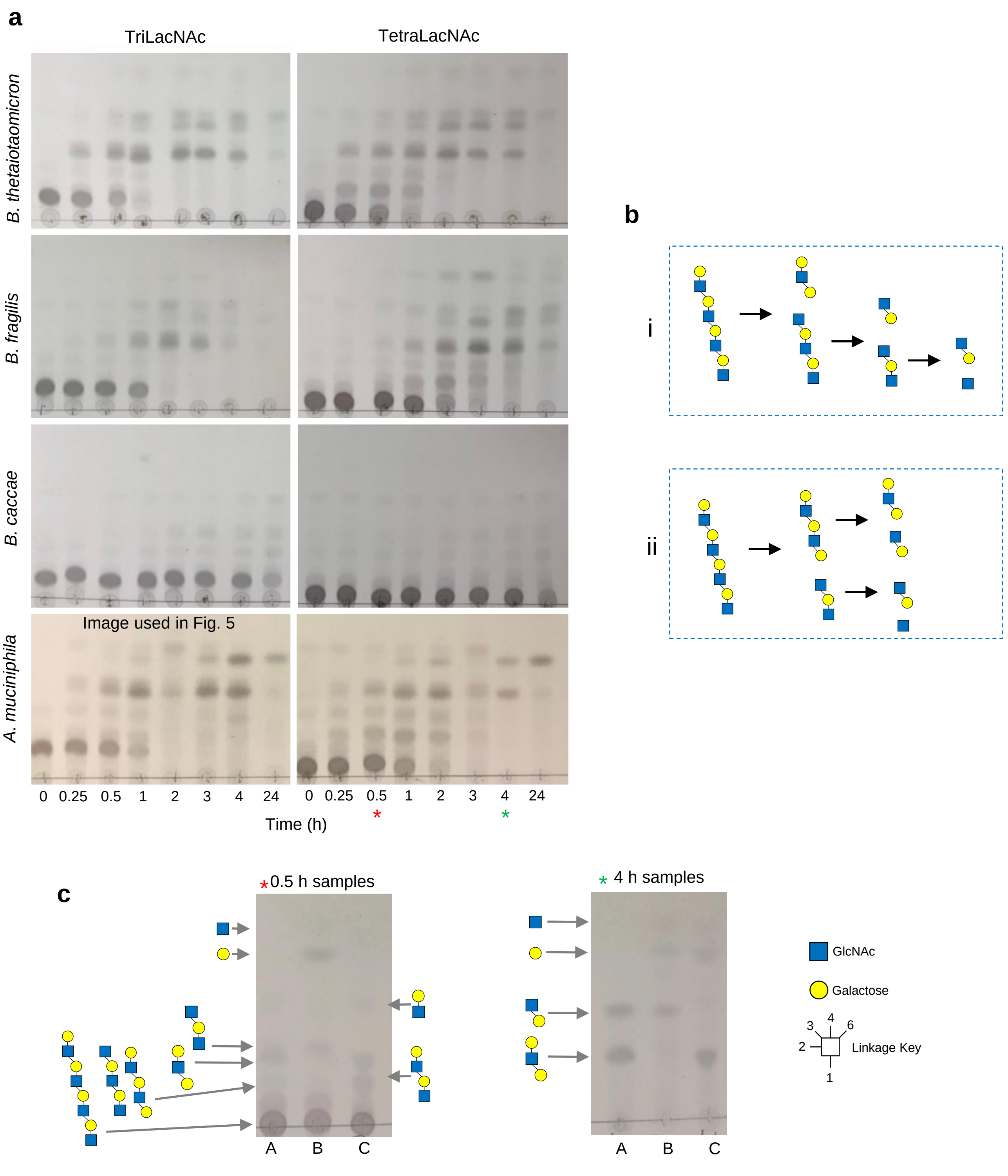

Supplementary Fig. 13 Whole cell assays showing GH16-like endo-activity on the cell surface of *Bacteroides* spp and *A. muciniphila*. a, The three *Bacteroides* spp. and *A. muciniphila* were grown on PGM III to mid exponential phase and the cells were harvested, washed, and surface enzyme activity was assessed by addition of TriLacNAc and TetraLacNAc to the metabolically inactive cells. **b**, Two sequences are possible for the degradation of TetraLacNAc by a GH16 O-glycanase, however, both sequences result in the same product profile. **c**, Samples from two different time points of the whole cell assay of *A. muciniphila* against TetraLacNAc were exposed to two different exo-acting enzymes of known specificity to verify the products. The asterisks indicate the original samples. A: original sample, B: + β1,4-galactosidase BT0461^{GH2}, C: + broad-acting β-GlcNAc'ase BT0459^{GH20}. The data produced from the 0.5 h sample reveals both possible sequences of degradation occur in *A. muciniphila.* These experiments were carried out once, but multiple pilot experiments were run and were consistent with the data shown. The source data are provided in the source data file.

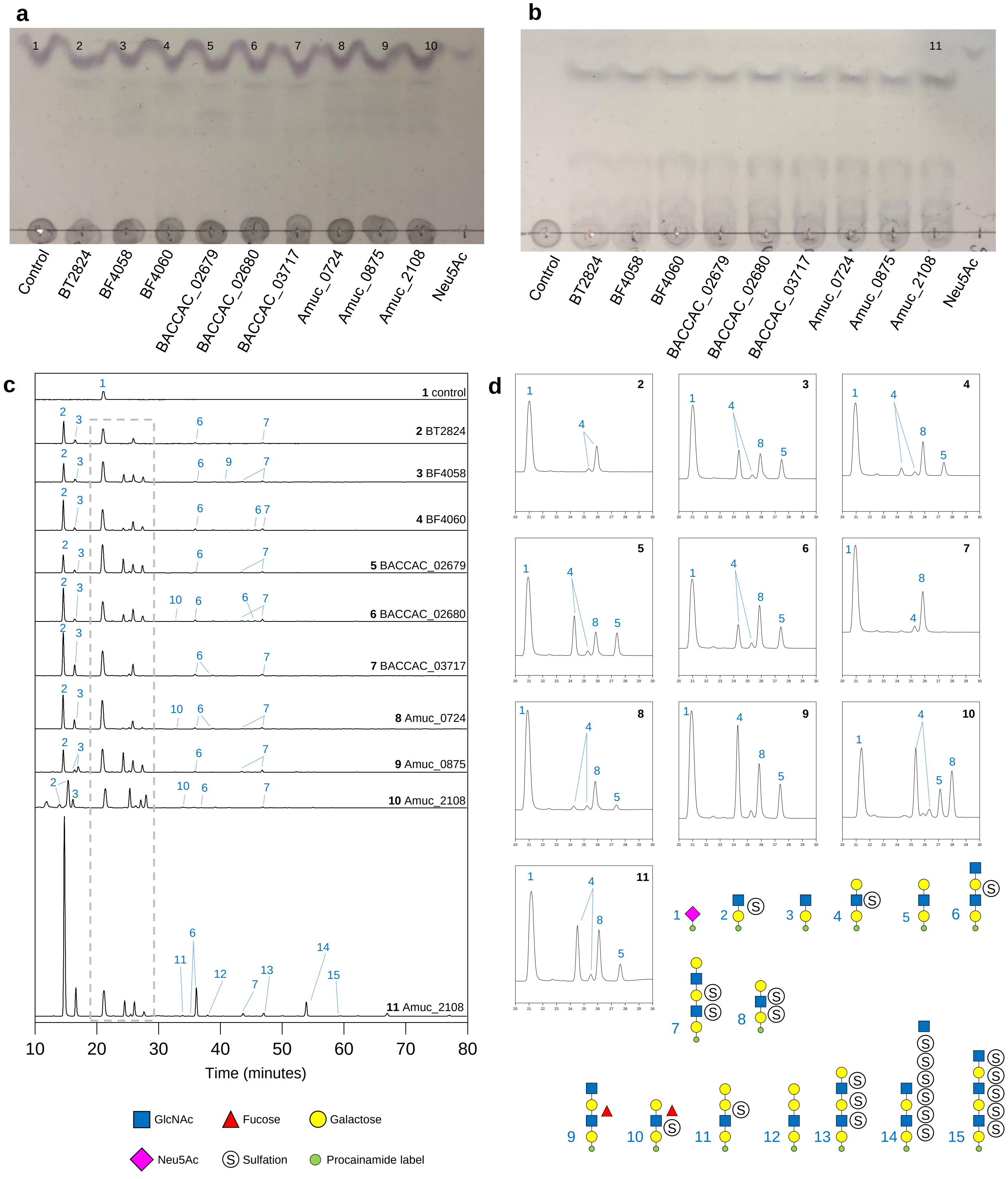

Supplementary Fig. 14 Activity of O-glycan active GH16 family members against keratin sulfate. a, TLC of activity of the different GH16 O-glycanases against N-linked egg keratan. The results are representative of two independent replicates. **b**, TLC of activity against N-linked bovine cornea keratan. **c**, Chromatograms of procainamide-labelled keratan products, where the number correspond to those in a and b. The grey dotted box indicated the areas which are magnified in d. **d**, Magnifications of the chromatograms between 20-30 minutes to allow clearer annotation of the products. All assays also included the broad-acting sialidase BT0455^{GH33}. The composition and structure of the products are shown. Source data are provided in the source data file.

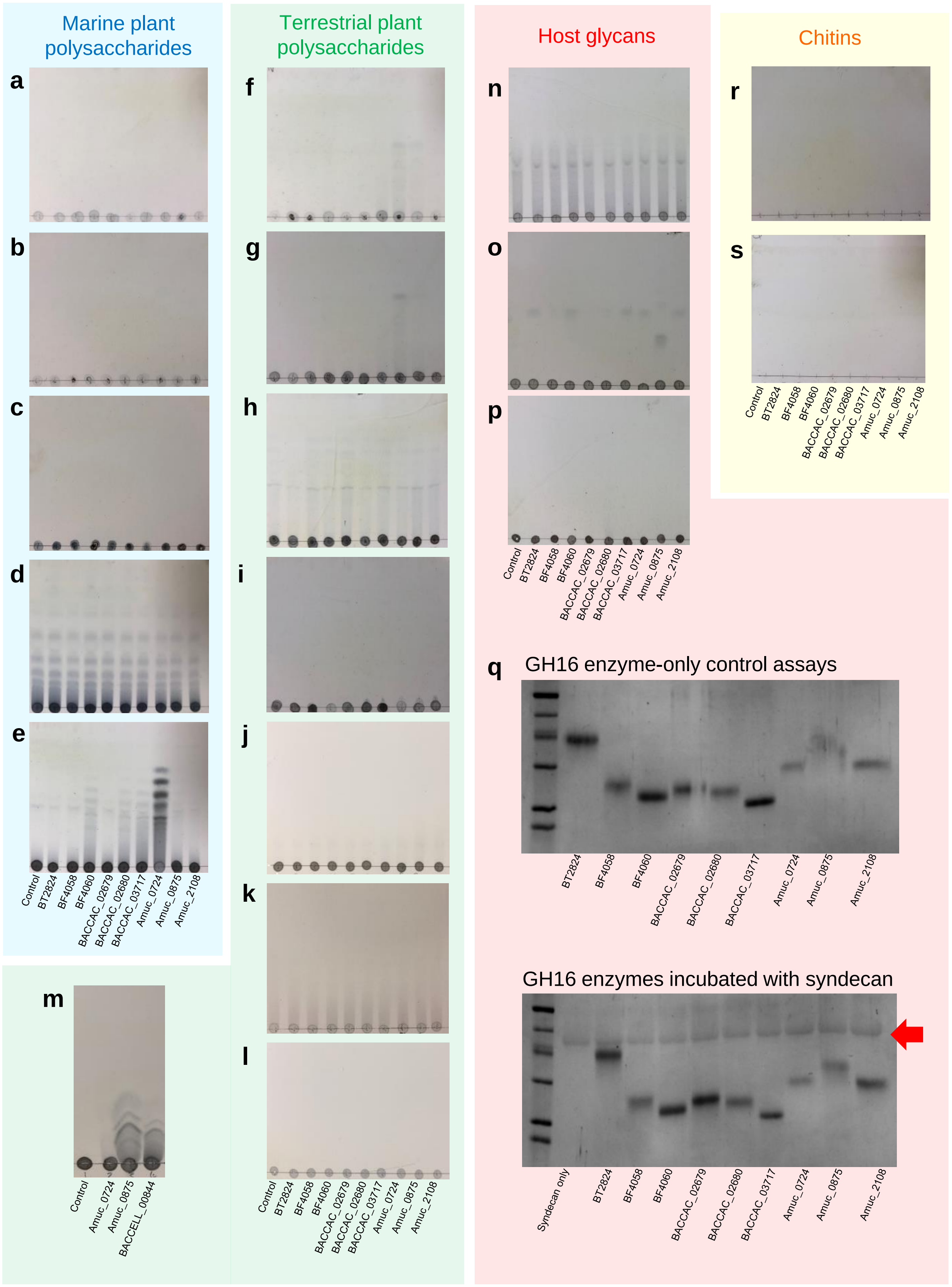

Supplementary Fig. 15 Activity of the GH16 O-glycanases against glycans previously shown to be GH16 substrates. Assays were carried out with **a**, Agar. **b**, Agarose. **c**, κ-carrageenan. **d**, Porphyran. **e**, Laminarin. **f**, Barley β-glucan. **g**, Lichenan. **h**, Pectic β1,4-galactan. **i**, Xyloglucan. **j**, Larch arabinogalactan. **k**, Wheat arabinogalactan. **l**, Gum arabinogalactan. **m**, β1,3-galactan **n**, Chondroitin sulfate. **o**, Heparan sulfate. **p**, Hyaluronic acid. **q**, Human syndecan. To assess if the GH16 enzymes could cleave the linker oligosaccharide between heparan sulfate and protein they were incubated with human syndecan (bottom gel). A control gel (top) shows GH16-only assays. The red arrow indicates where syndecan (50 μg) runs on the gel and shows that it does not decrease in mass with the addition of GH16. **r**, Shrimp chitin. **s**, Squid chitin. Assay were 10 mg ml^{-1} substrate, 3 μM enzyme, 20 mM MOPS, pH 7.0 at 37 °C overnight. 3 μl was spotted on to the TLC. The results are representative of two independent replicates. Source data are provided in the source data file.

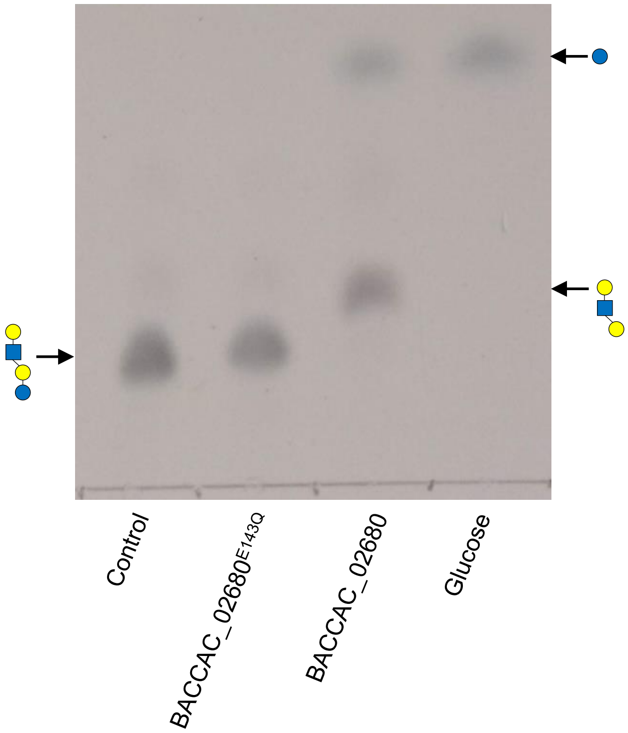

Supplementary Fig. 16 Activity of nucleophile mutant of BACCAC_02680 against lacto-N-*neo*tetraose. The predicted catalytic nucleophile of BACCAC_02680 (E143) was mutated to glutamine and the activity of the mutant determined against lacto-N-*neo*tetraose (10 µM mutant or 0.1 µM wild type enzyme added to 1mM substrate in 20 mM MOPS buffer pH 7.0, at 37 ºC overnight). BACCAC_02680^{E143Q} was used in co-crystallisation trials with TriLacNAc and despite the lack of activity observed here the substrate was cleaved in crystal and only the trisaccharide product was bound in the co-crystal structure. The results are representative of two independent replicates. Source data are provided in the source data file.

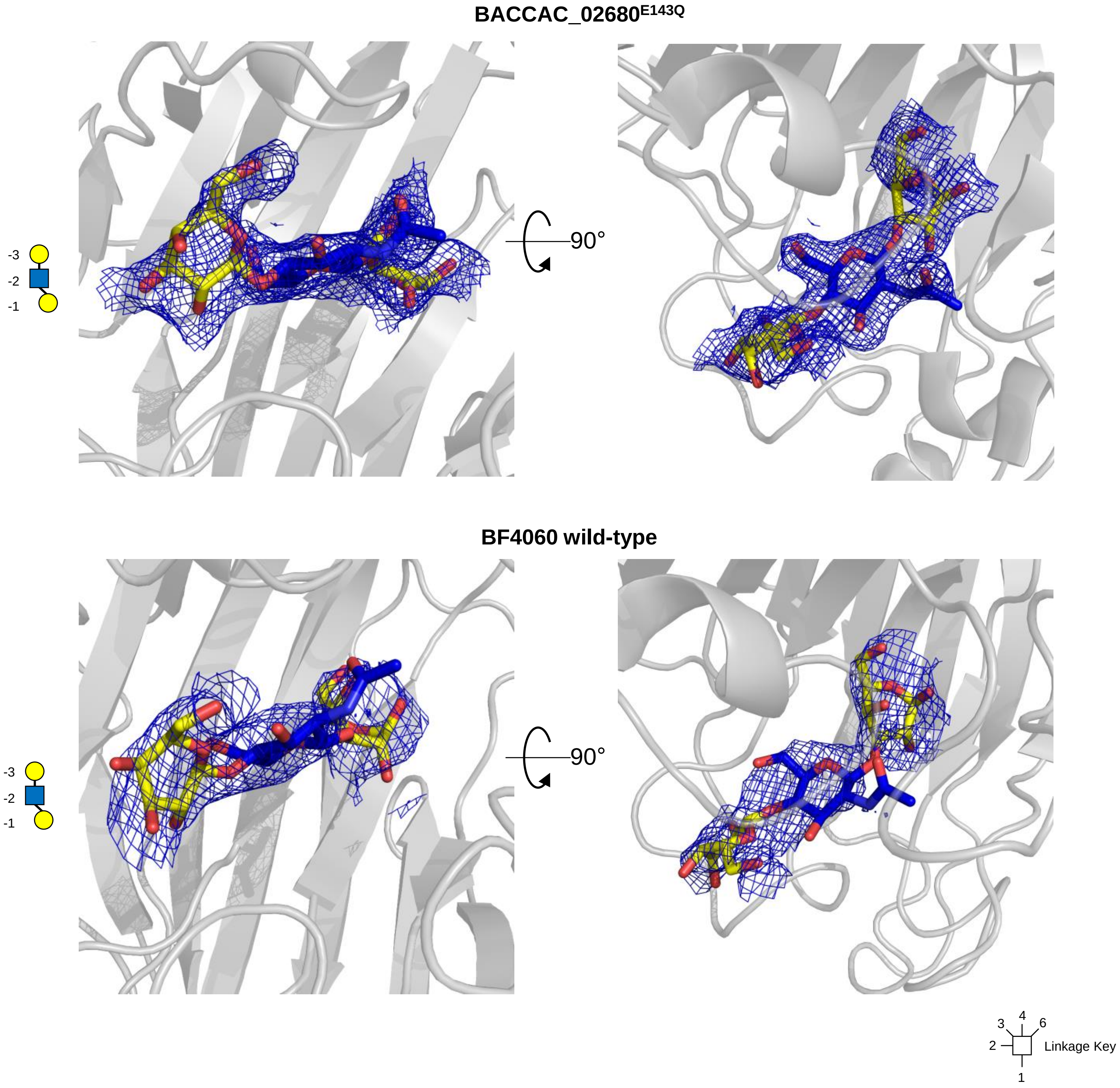

Supplementary Fig. 17 Omit maps for the trisaccharide products in the BACCAC_02680^{E142Q} and BF4060 structures. Omit maps were produced to show the electron densities of the product. See Supplementary Table 7 for sugar conformational validation using Privateer (Methods). Galactose and GlcNAc are shown as yellow and blue sticks in the model, respectively. Bound products are also displayed as symbols next to the structures and the subsites the product occupies are indicated.

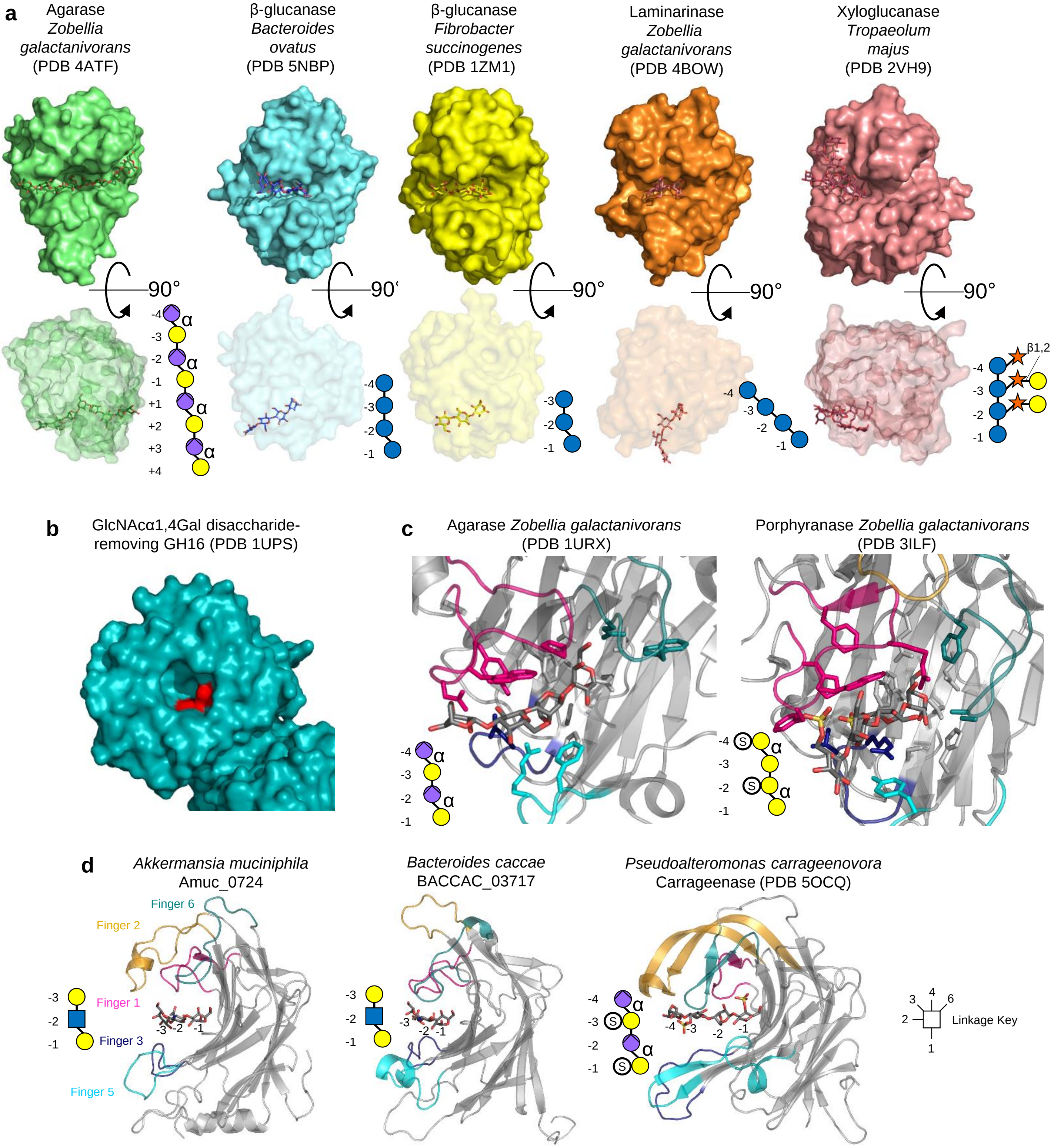

Supplementary Fig. 18 Structural differences between GH16 enzymes. a, Crystal structures of a number of different GH16 family members to illustrate the variation in terms of cleft structure and orientation of substrate in the family (Heheman *et al.* 2012, Tamura *et al.* 2017, Tsai *et al.* 2005, Labourel *et al.* 2014 and Mark *et al.* 2009). **b**, The substrate binding site of *Clostridium perfringens* GH16 family member that removes GlcNAcα1,4Gal disaccharide from stomach mucins. This pocket-like binding site is a unique example amongst GH16 enzyme structures so far. Catalytic residues are shown in red (Tempel *et al.* 2005). **c**, The active sites of two GH16 enzymes with activity towards marine plant polysaccharides. This demonstrates the differences in the negative subsites to GH16 β-glucanases. Both enzymes select for a β1,3 linkage between the -1 and -2 subsite, but the structural features driving this are different in each. **d**, Side views of Amuc_0724, BACCAC_03717 and a Carrageenase (Matard-Mann *et al.* 2017) to demonstrate the different size and positions of finger two (yellow). Finger 2 from the Carrageenase interacts with sugars occupying the -3 and -4 subsites and Finger 2 from Amuc_724 and BACCAC_03717 have the potential to do the same. All bound substrates or products are also represented by symbols next to the structures with the subsites occupied by each sugar indicated. All diagrams follow the linkage key except for xyloglucan, where the linkage between the xylose and galactose is indicated.

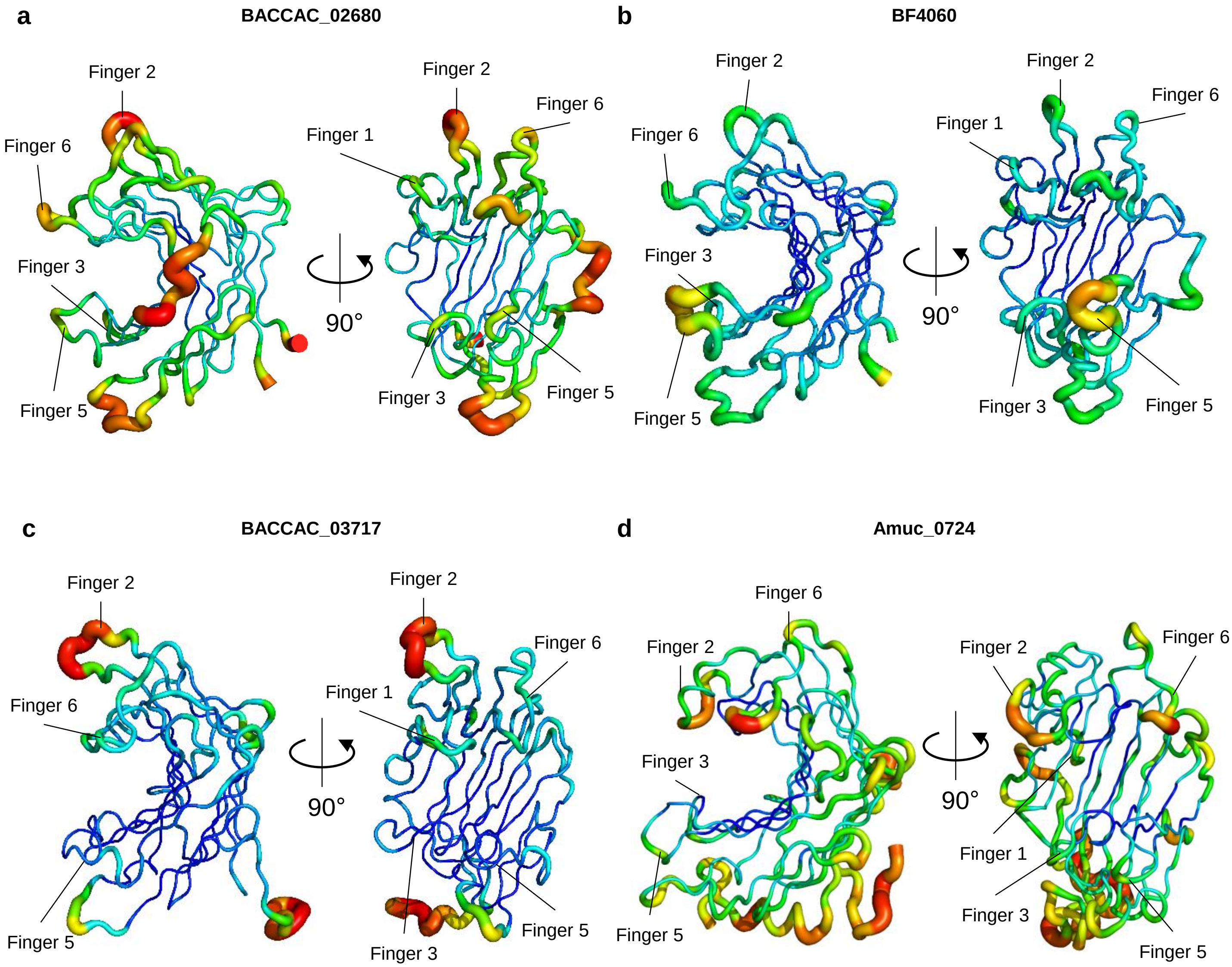

Supplementary Fig. 19 B factor putty projections of the O-glycan active GH16 crystal structures determined in this study. The protein backbones are colour and thickness coded from thin-blue to thick-red depending on the level of flexibility in the structure. For example, the core β-sheet folds of the enzymes tend to be much more static (blue and thin) than the loops extending away from the core (red and thick). **a-d** The structures of BACCAC_02680, BF4060, BACCAC_03717, and Amuc_0724, respectively.

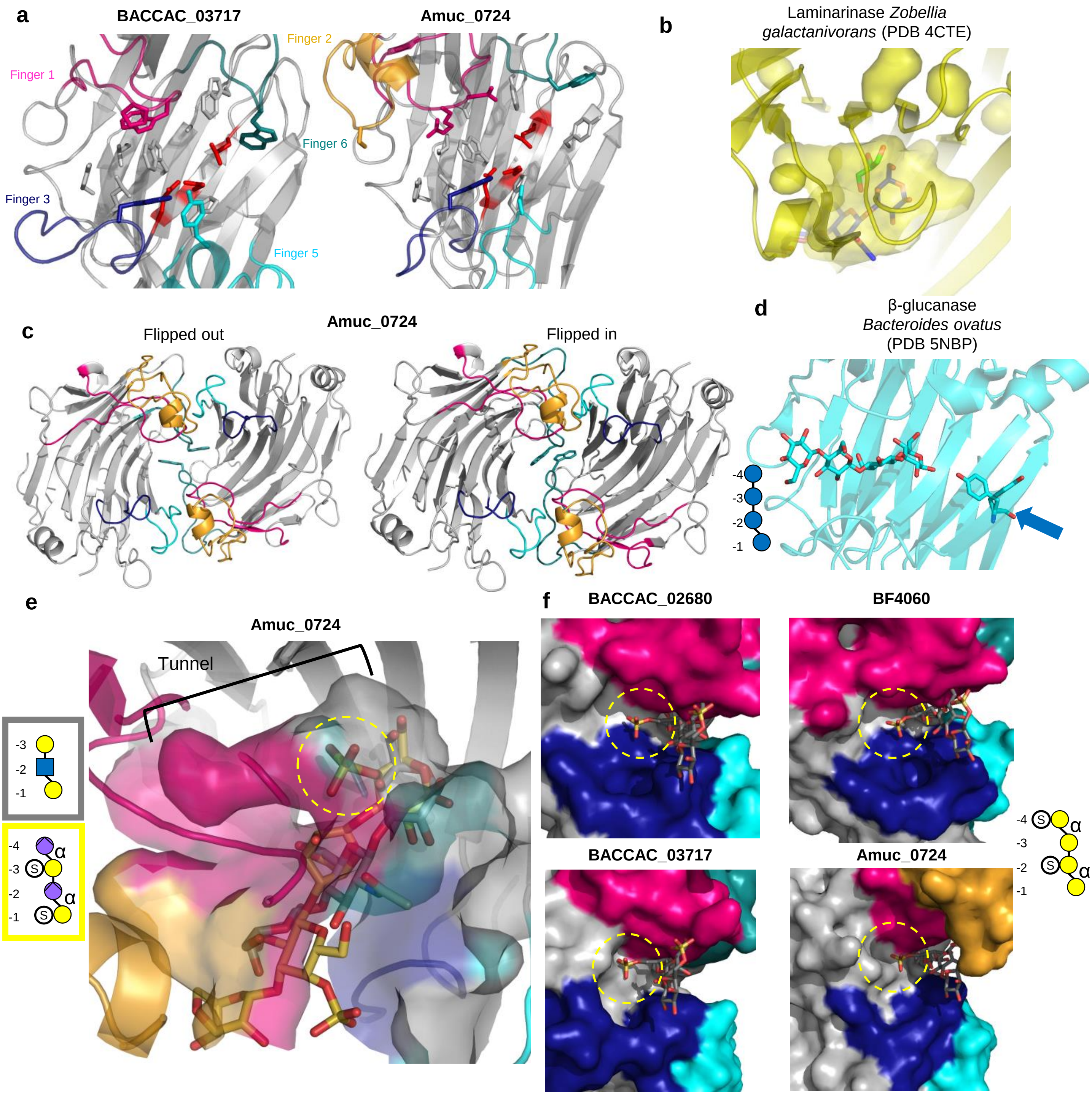

Supplementary Fig. 20 Structural comparisons between GH16 O-glycanases and GH16 enzymes with different specificities. a, Active sites of BACCAC_03717 and Amuc_0724. The fingers are colour coded. **b**, A surface representation of the cavities and pockets around the -1 subsite of a laminarinase (Labourel *et al.* 2015). A glycerol was crystallised within the pocket adjoining the -1 subsite and the product from BACCAC_02680^{GH16E143Q} is overlaid to show the -1 subsite. The presence of the glycerol suggested that sugar decorations may be accommodated at this position. **c**, The two different occupancies possible for W279 in Amuc_0724 in the unit cell to show the interactions between the two molecules. The tyrosines are show as sticks and come from finger 6 (teal). The flipped-out version may represent a crystallographic artefact rather than a biologically relevant observation. **d**, A dynamic tyrosine (blue arrow) is also observed in the positive subsites of a GH16 β-glucanase from *B. ovatus*. **e**, Surface representation of the pockets and cavities around the negative subsites of the Amuc_0724 crystal structure. The different colours represent the different fingers of the enzyme. The trisaccharide product from BC02680^{E143Q} (grey) is overlaid with Amuc_0724 as well as the carrageenan product (yellow) from a κ-carrageenases from *Pseudoalteromonas carrageenovora* (PDB 5OCQ; Matard-Mann *et al.* 2017) to show how a sulfate could be accommodated in the tunnel of the -1 subsite Amuc0724 (yellow dashed circle). Accommodation of this sulfate group in the -1 subsite would not be possible with the O-glycan active GH16 enzymes from *Bacteroides* spp. **f**, The four O-glycan active GH16 enzymes from this study are overlaid with the porphyran product originally crystallised with a porphyranase from *Zobellia galactanivorans* (PDB 3ILF; Heheman *et al.* 2010), demonstrating how a sulfate group could be accommodated in the cleft at the -2 subsite of the O-glycanases (yellow dashed circles). All bound substrates or products are also represented with a sugar diagram and the subsites are indicated.

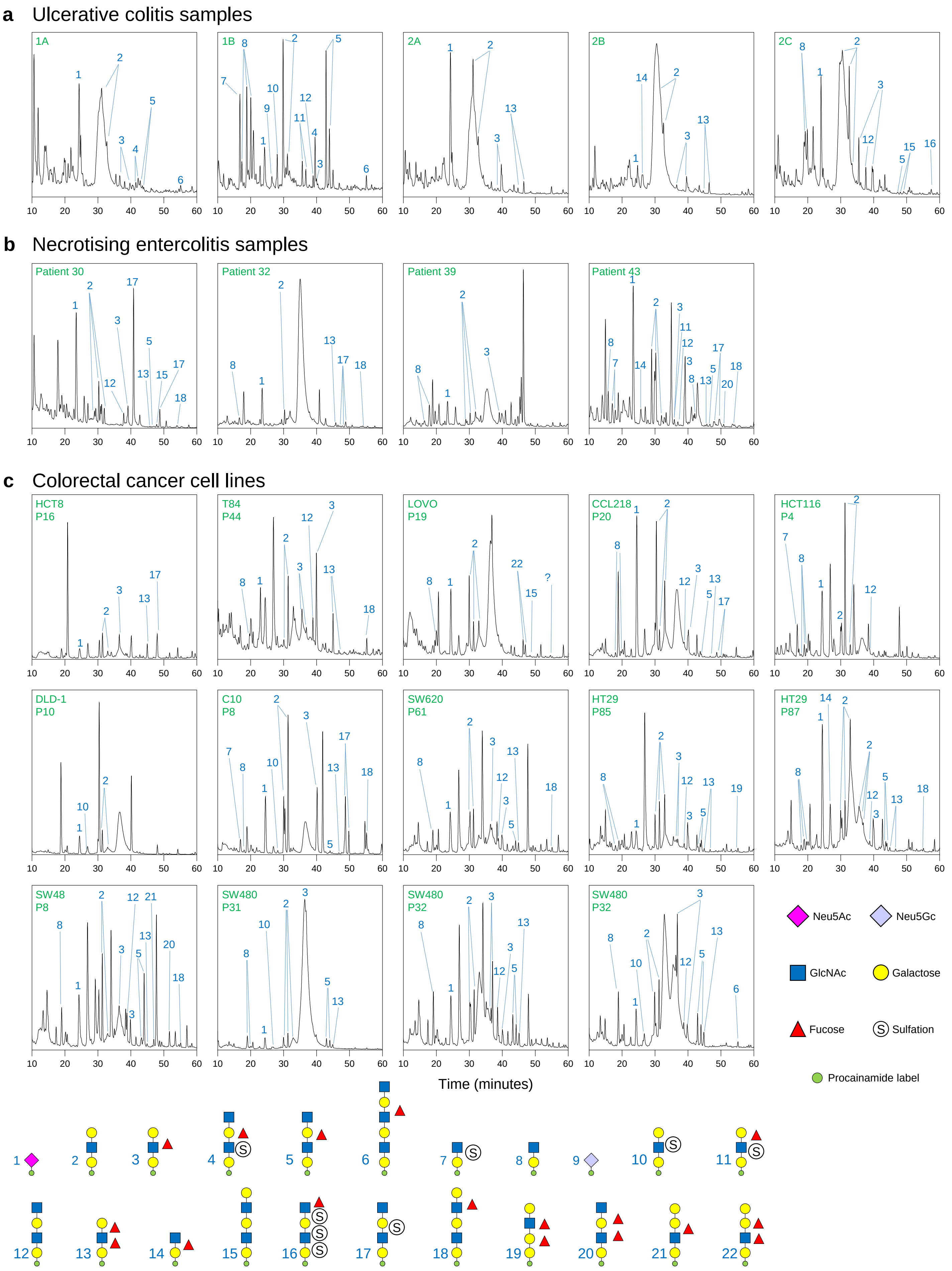

a Ulcerative colitis samples
1A
1B
2A
2B
2C
b Necrotising entercolitis samples
Patient 30
Patient 32
Patient 39
Patient 43
c Colorectal cancer cell lines
HCT8 P16
T84 P44
LOVO P19
CCL218 P20
HCT116 P4
DLD-1 P10
C10 P8
SW620 P61
HT29 P85
HT29 P87
SW48 P8
SW480 P31
SW480 P32
SW480 P32
Neu5Ac
Neu5Gc
GlcNAc
Galactose
Fucose
Sulfation
Procainamide label
Time (minutes)

Supplementary Fig. 21 Products released from human mucins by Amuc_0724 O-glycanase. Products of mucin digestion were labelled with procainamide at the reducing end and analysed by LC-FLD-ESI-MS (Methods). All samples were pre-treated with the broad acting sialidase BT0455^{GH33} **a**, Products of GH16 digestion of mucin samples from two patients with ulcerative colitis. Patient 1 had a laproascopic panproctocolectomy and all removed colon was inflamed. Sample 1A is uninflamed ileum and 1B from inflamed colon. Patient 2 had a laprascopic ileocaecal resection. Sample 2A inflamed ileum (bowel terminal ileum), 2B from uninflamed ileum (near small bowel staple line) and 2C from uninflamed colon (ascending). **b**, Mucin from neonates with necrotising enterocolitis. Each sample is a different patient **c**, Mucin from colorectal cancer cell lines. Small amounts of Neu5Gc are seen in some of the ulcerative colitis samples (e.g. 1A) suggesting either the presence of contaminating dietary animal O-glycans remaining in the mucus layer or that this xenobiotic sugar has been incorporated into human mucins from dietary sources.

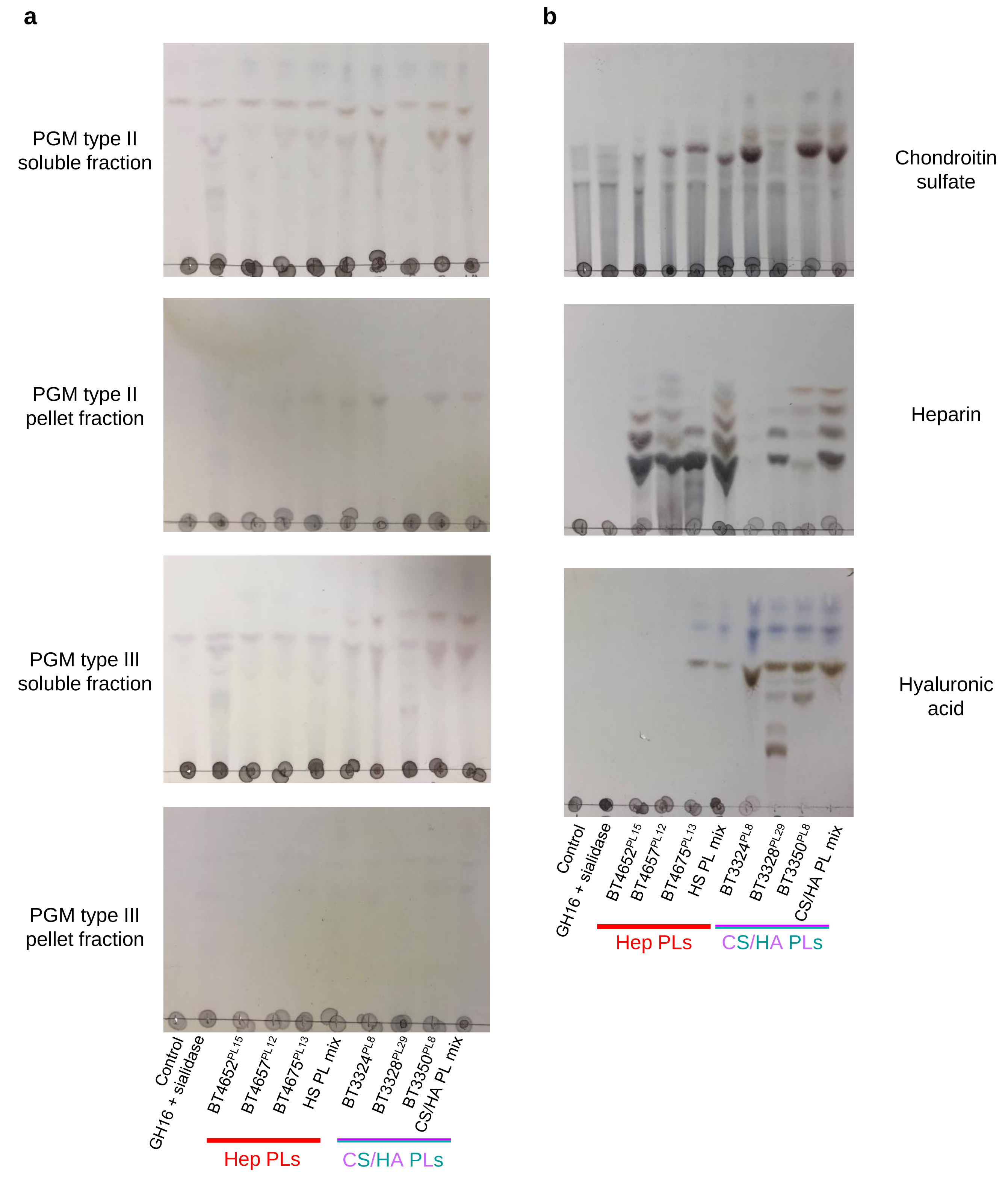

Supplementary Fig. 22 Analysis of the glycosaminoglycan (GAG) content of porcine gastric mucin (PGM) type II and III. a, The polysaccharide lyases (PLs) from *B. thetaiotaomicron* involved in or predicted to be involved in the degradation of Hep, CS and HA were used to test for the presence of these GAGs in PGM types II and III (Cartmell *et al.* 2017; Ndeh *et al.* 2018). PGM was dissolved in H_2O at 50 mg ml^{-1} then centrifuged at 16000 x g for 30 min. The supernatant (soluble) and pellet fractions were assayed separately. Each PL was tested individually and then as a mix. The red line and purple/teal line represents the HS and CS/HA active lyases, respectively. **b,** The PLs were tested against pure GAGs as controls. Assay were 3 μM enzyme, 20 mM MOPS, pH 7.0 at 37 °C overnight. 3 μl of the assay was spotted on to the plate before running. The results are representative of two independent replicates. Source data are provided in the source data file.

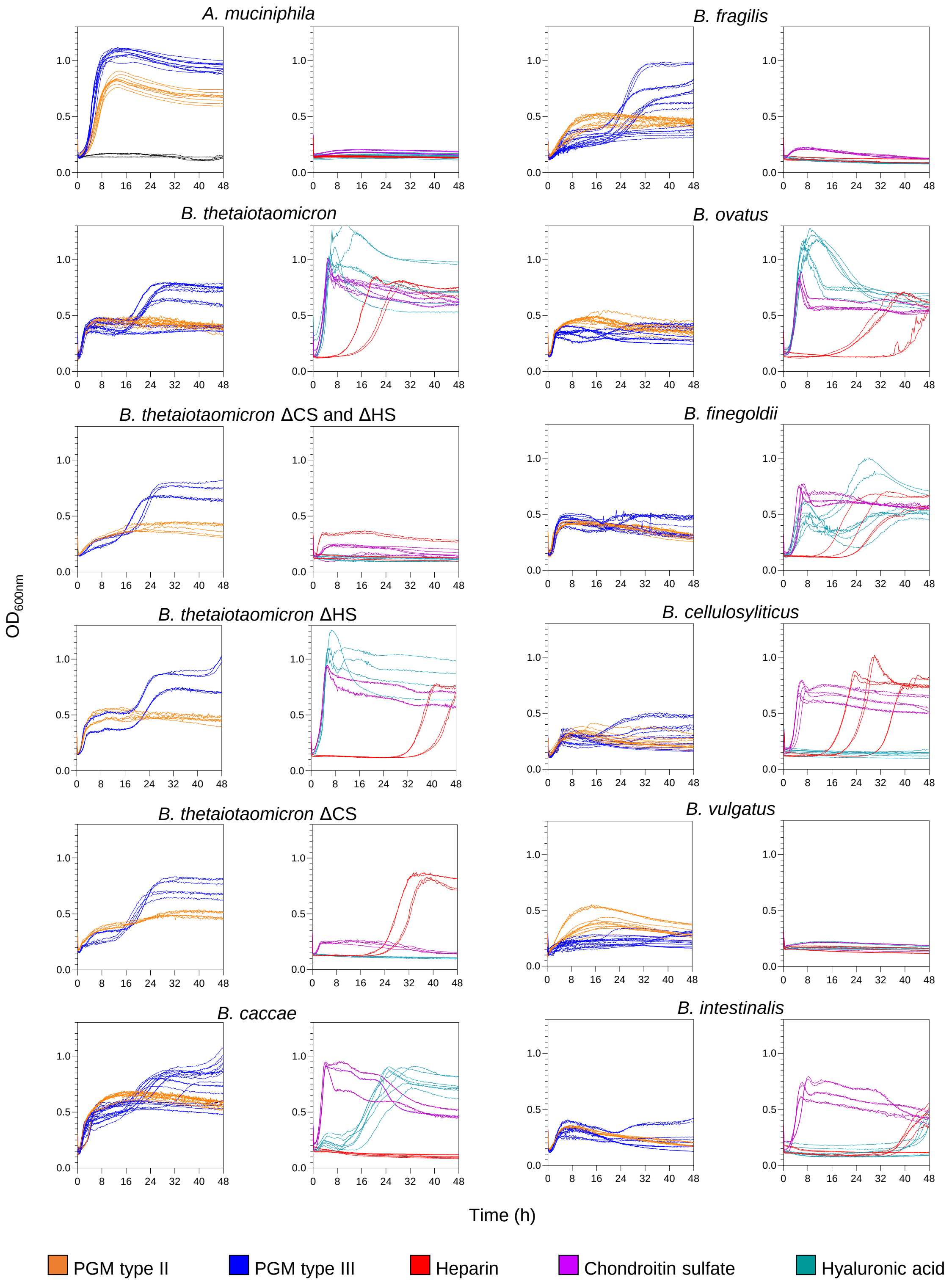

Supplementary Fig. 23 Growth of *Bacteroides* spp and *A. muciniphila* on host glycans. *A.* Bacteria were grown anaerobically in minimal media containing a range of host derived glycans as the sole carbon source. PGM type II, PGM type III, Heparin, chondroitin sulfate and hyaluronic acid were at 35, 40, 20. 20 and 10 mg ml^{-1}, respectively. Growth (OD^{600}) was monitored continuously using a plate reader. ΔCS and ΔHS are mutant strains of *B. theta* lacking either the chondroitin sulfate/hyaluronic acid (CS) PUL, or heparin/heparan sulfate (HS) PUL. The strains used are listed in Supplementary Table 3.

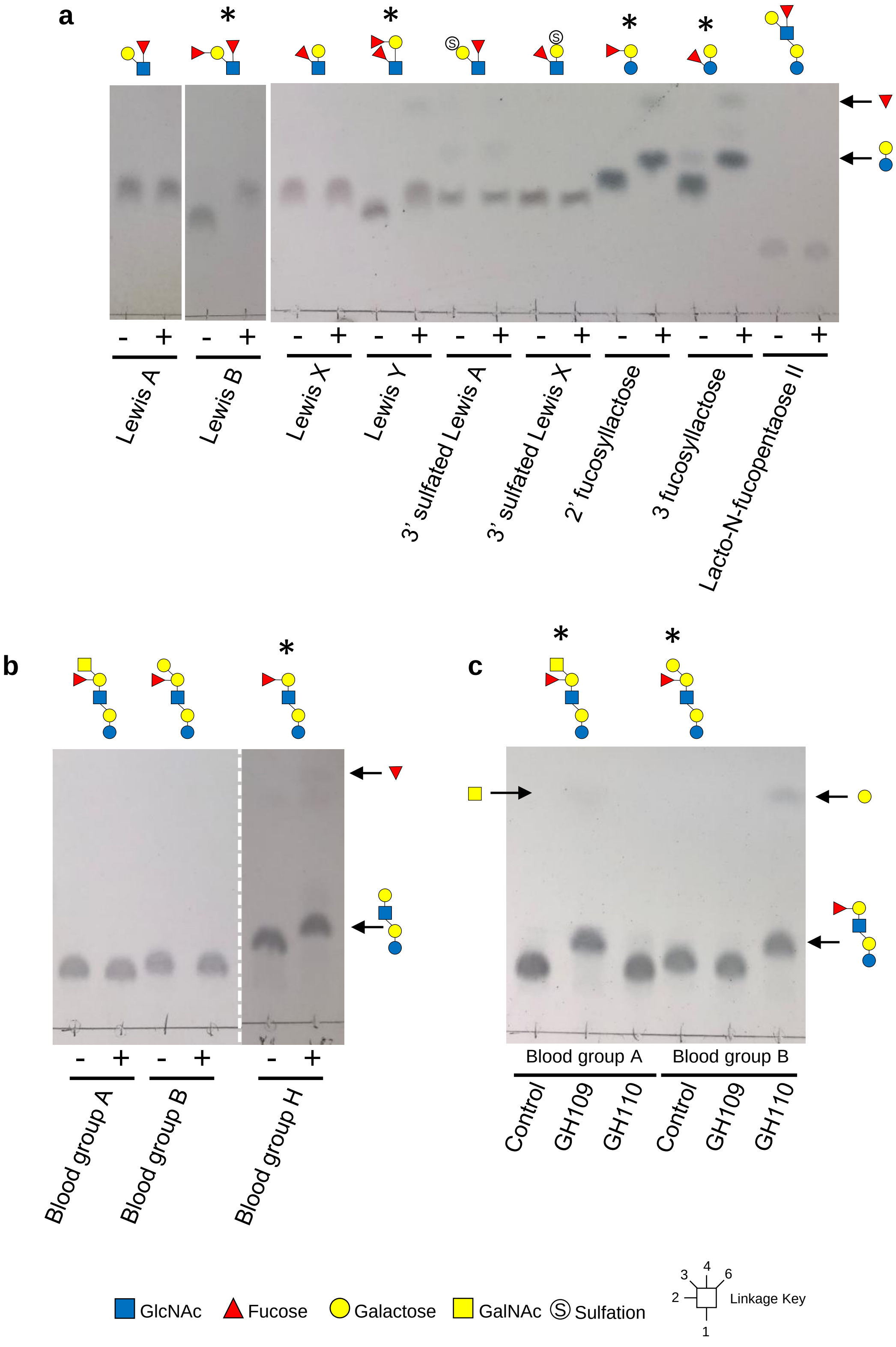

Supplementary Figure 24│Activity of exo-acting enzymes used to characterise GH16 products *a*,*b* Activity of GH95 α-fucosidase from *Bifidobacterium bifidum* against a range of fucosylated oligosaccharides. With blood group sugars (b), activity can only be seen after removal of the α-linked GalNAc or Gal (i.e. against blood group H only). **c**, Activity of a GH109 α-GalNAc'ase and GH110 α-galactosidase (Methods) against blood group A and B oligosaccharides. Assays were 1 mM substrate and 3 μM enzyme in 20 mM MOPS, pH 7.0 at 37 °C overnight. 3 μl of the assay was spotted at the origin. Oligosaccharides that are substrates for the different enzymes are indicated by an asterisk. The results are representative of two independent replicates. Source data are provided in the source data file.

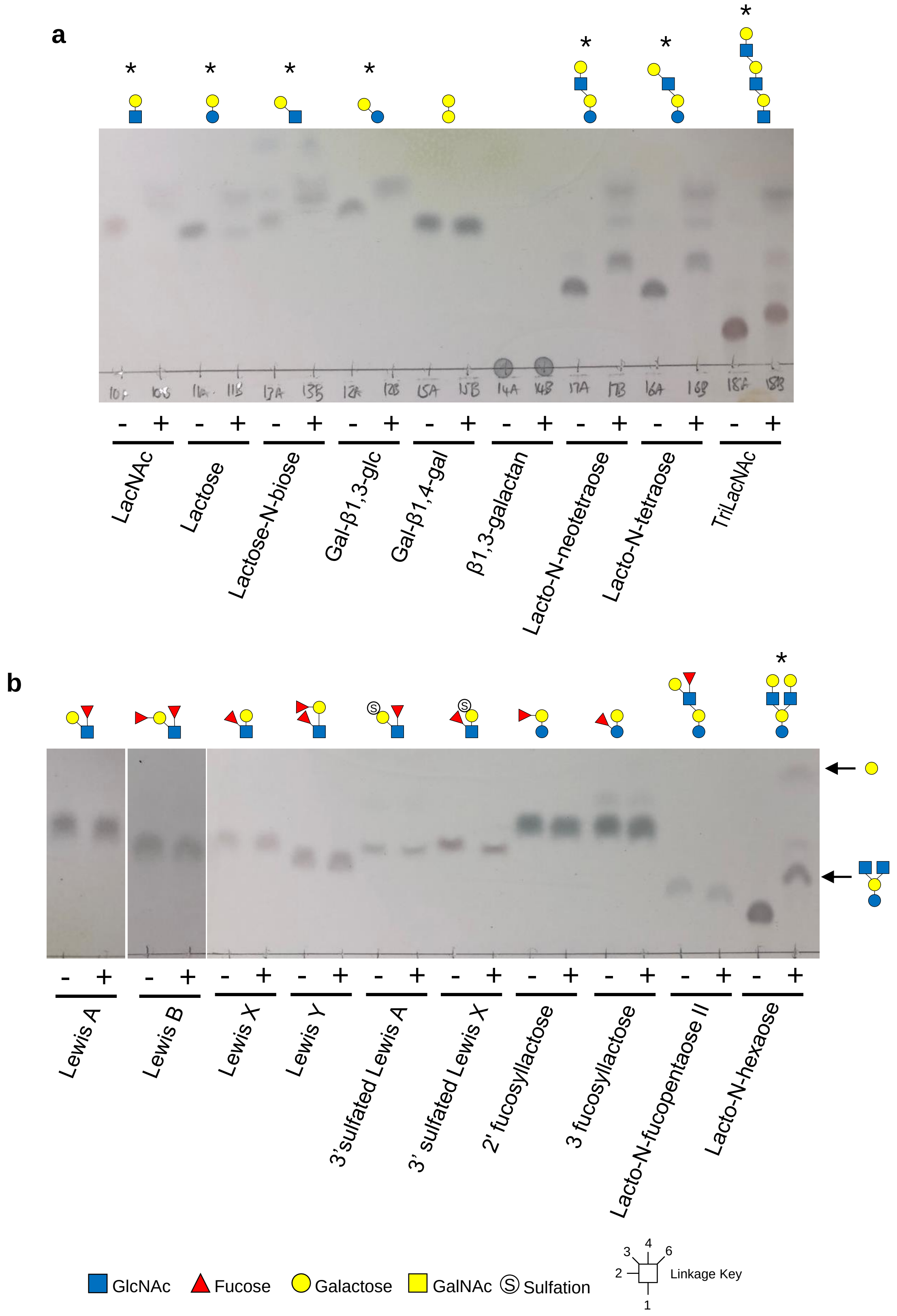

Supplementary Figure 25│Activity of BF4061^{GH35} against β-galactose containing di- and oligosaccharides The polysaccharide utilisation loci in *B. fragilis* encoding the O-glycan active GH16 enzymes also encodes an uncharacterised GH35 enzyme, BF4061, that was predicted to be a β-galactosidase (see Fig. 1). ***a,b*** BF4061^{GH35} was incubated with a variety of saccharides to determine specificity. Assays contained 1 mM substrate, 3 μM enzyme in 20 mM MOPS, pH 7.0 at 37 °C overnight. 3 μl of the assay was spotted onto the origin. Saccharides that are substrates for the GH35 are indicated by an asterisk. The results are representative of two independent replicates. Source data are provided in the source data file.

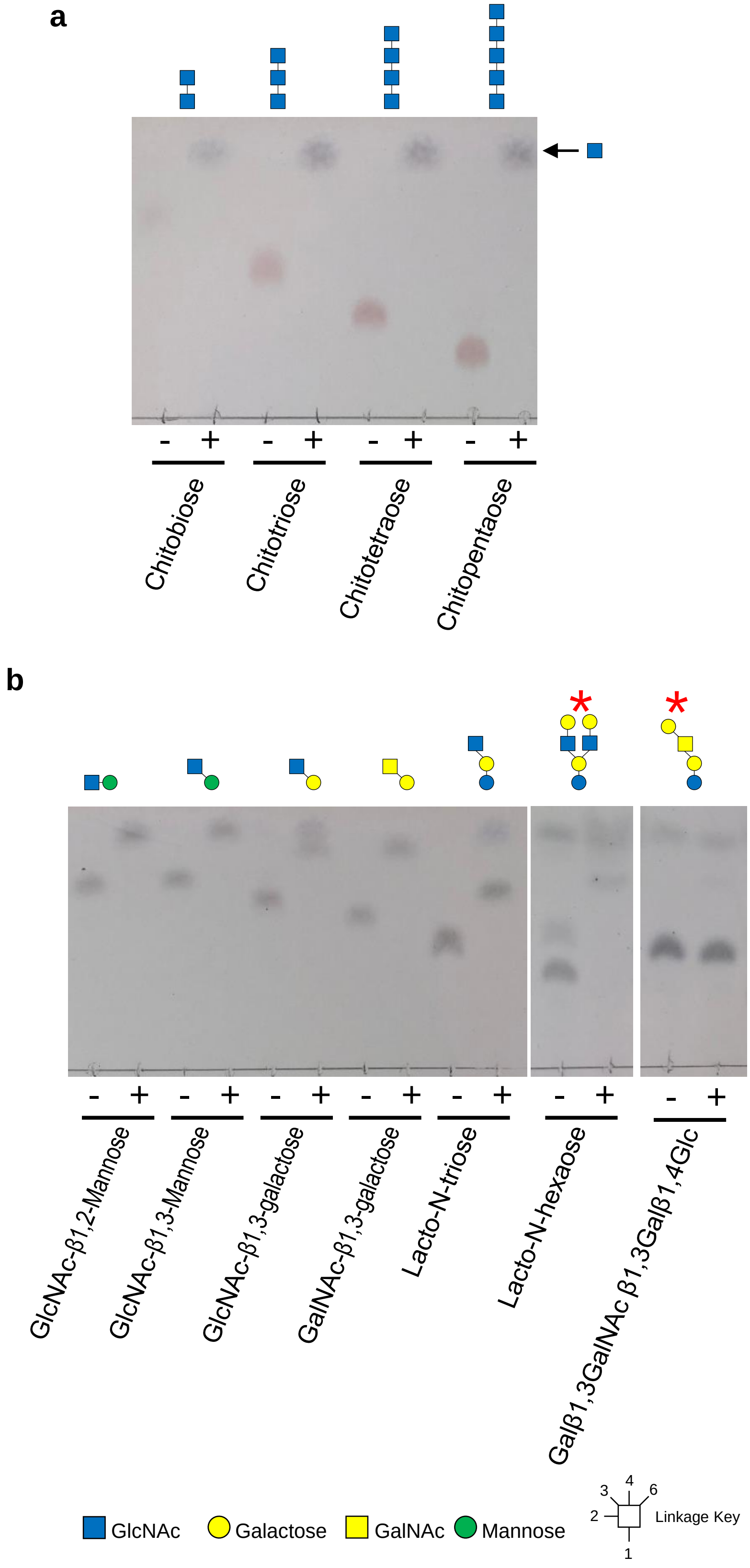

Supplementary Fig. 26 Activity of BF4059^{GH20} against β-HexNAc containing di- and oligo-saccharides The PUL in *B. fragilis* encoding the O-glycan active GH16 enzymes also encodes an uncharacterised GH20 enzyme, BF4059, that was predicted to be a β-hexosaminidase (see Fig. 1). **a,** Activity of BF4059^{GH20} against chitooligosaccharides and **b,** against O- and N-glycan-derived saccharides. The red asterisk indicates Lacto-N-hexaose and Galβ1,3GalNAcβ1,3Galβ1,4Glc were pre-treated with BF4061^{GH35} to remove the non-reducing end galactose before assaying against BF4059. Assays were 1 mM substrate, 3 μM enzyme, 20 mM MOPS, pH 7.0 at 37 °C overnight. 3 μl of the assay was spotted on to the origin. The GH20 was active against all glycans tested except for GalNAcβ1,3Galβ1,4Glc. The results are representative of two independent replicates. Source data are provided in the source data file.

Supplementary Tables

Supplementary Table 1. Signal peptide predictions for the CAZymes characterised in this study.

Species	Locus Tag	CAZy family	Predicted signal peptide[a]	Experimentally determined location
B. thetaiotaomicron	BT2824	GH16	SPII	
B. fragilis	BF4058	GH16	SPII	
B. fragilis	BF4059	GH20	SPII	
B. fragilis	BF4060	GH16	SPI	
B. fragilis	BF4061	GH35	SPI	
B. caccae	BACCAC_02679	GH16	SPII	
B. caccae	BACCAC_02680	GH16	SPI	
B. caccae	BACCAC_03717	GH16	SPII	
A. muciniphila	Amuc_0724	GH16	SPI/II[b]	
A. muciniphila	Amuc_0875	GH16	SPI	
A. muciniphila	Amuc_2108	GH16	SPI[c]	Outer membrane[21]

[a]Predictions carried out using SigP5.0.
[b]SigP5.0 predicts SPI most likely (0.67) for Amuc_0724, but could also be SPII (0.33). This appears likely due to the presence of two cysteine residues near the end of the signal sequence.
[c]Amuc_2108 has a very hydrophobic region at the C-terminus that could be a membrane anchor.

Supplementary Table 2. Percentage identity between all GH16 family members included in this study

	BT2824	BF4058	BF4060	BACCAC_02679	BACCAC_02680	BACCAC_03717	Amuc_0724	Amuc_0875	Amuc_2108
BT2824	100 %	38 %	37 %	38 %	38 %	35 %	27 %	25 %	30 %
BF4058		100 %	28 %	87 %	27 %	31 %	29 %	26 %	29 %
BF4060			100 %	29 %	79 %	31 %	26 %	24 %	29 %
BACCAC_02679				100 %	27 %	31 %	29 %	27 %	29 %
BACCAC_02680					100 %	31 %	24 %	22 %	28 %
BACCAC_03717						100 %	30 %	28 %	36 %
Amuc_0724							100 %	34 %	29 %
Amuc_0875								100 %	23 %
Amuc_2108									100 %

Supplementary Table 3. List of strains used in this study

Species	Strain
Akkermansia muciniphila	ATCC
Bacteroides caccae	ATCC43185
Bacteroides cellulosilyticus	DSM 14838
Bacteroides fragilis	NCTC 9343
Bacteroides finegoldii	DSM 17565
Bacteroides intestinalis	341, DSM 17393
Bacteroides thetaiotaomicron	VPI-5482
Bacteroides ovatus	ATCC 8482
Bacteroides vulgatus	ATCC 8483

Supplementary Table 4. Activity of GH16 O-glycanases against defined oligosaccharides

		BT2824	BF4058	BF4060	BACCAC_02679	BACCAC_02680	BACCAC_03717	Amuc_0724	Amuc_0875	Amuc_2108
Linear substrates	**TriLacNAc** spanning -3 to +3 subsites (Supplementary Fig. 11a)	Yes	Yes	Yes	Yes	Yes	Yes	Yes	Partial	Yes
	**TriLacNAc product** GlcNAcβ1,3Galβ1,4GlcNAc spanning the -2 to +1 subsites (Supplementary Fig. 11a)	Yes	Yes	Yes	Yes	Yes	Yes	Yes	Partial	Yes
	**paraLacto-N-neohexaose** spanning the -3 to +3 subsites (Supplementary Fig. 11b)	Yes	Yes	Yes	Yes	Yes	Yes	Yes	Partial	Yes
	**paraLacto-N-neohexaose product** GlcNAcβ1,3Galβ1,4Glc spanning the -2 to +1 subsites (Supplementary Fig. 11b)	Yes	Yes	Yes	Partial	Yes	Yes	Yes	No	Yes
	**Lacto-N-neotetraose** spanning the -3 to +1 subsites (Supplementary Fig. 11c)	Yes Reduced rate relative to TriLacNAc Preference over LNT	Yes Very reduced rate relative to TriLacNAc	Yes Very reduced rate relative to TriLacNAc	Yes Very reduced rate relative to TriLacNAc	Yes Reduced rate relative to TriLacNAc	Yes Unaffected relative to TriLacNAc	Yes Reduced rate relative to TriLacNAc Preference over LNT	No	Yes Reduced rate relative to TriLacNAc
	**Lacto-N-neotetraose product** Galβ1,4GlcNAcβ1,3Gal spanning the -1 to +2 or -2 to +1 subsites (Supplementary Fig. 11c)	Yes	No	No	No	No	Yes	Partial	No	Partial
	**Lacto-N-tetraose** spanning the -3 to +1 subsites (Supplementary Fig. 11d)	Yes Reduced rate relative to TriLacNAc	Yes Very reduced rate relative to TriLacNAc	Yes Very reduced rate relative to TriLacNAc	Yes Very reduced rate relative to TriLacNAc	Yes Reduced rate relative to TriLacNAc Preference over LNnT	Yes Unaffected relative to TriLacNAc	Yes Reduced rate relative to TriLacNAc	No	Yes Reduced rate relative to TriLacNAc
	**Lacto-N-tetraose product** Galβ1,3GlcNAcβ1,3Gal spanning the -1 to +2 or -2 to +1 subsites (Supplementary Fig. 11d)	No	No	Partial	No	Partial	No	Partial	No	No
	**Lacto-N-triose** spanning the -2 to +1 subsites (Supplementary Fig. 12g)	Yes	Yes	Yes	Partial	Yes	Yes	Yes	No	Yes
	**LacNAc** (Supplementary Fig. 12a)	No	No	No	No	No	No	No	No	No
	**Galβ1,4Gal**	No	No	No	No	No	No	No	No	No

	(Supplementary Fig. 12c)									
	**Lacto-N-biose**<br>(Supplementary Fig. 12d)	No	No	No	No	No	No	No	No	No
	**Lactose**<br>(Supplementary Fig. 12f)	No	No	No	No	No	No	No	No	No
α-linked sugar at the -2 subsite	**Blood group H trisaccharide**<br>α1,2 fucose at the -2 subsite<br>(Supplementary Fig. 12b)	No	No	No	No	No	No	No	No	No
	**3-Sialyllactose**<br>α2,3 sialic acid at the -2 subsite<br>(Supplementary Fig. 12i)	No	No	No	No	No	No	No	No	No
	**P1 antigen**<br>α1,4 Galactose at the -2 subsite<br>(Supplementary Fig. 12e)	No	No	No	No	No	No	No	No	No
	**Globotriose**<br>α1,4 Galactose at the -2 subsite<br>(Supplementary Fig. 12f)	No	No	No	No	No	No	No	No	No
	**Forssman antigen**<br>α1,4 Galactose at the -2 subsite<br>(Supplementary Fig. 12j)	No	No	No	No	No	No	No	No	No
α1,4 fucose branch at the -3' subsite	**Lacto-N-fucopentaose II**<br>spanning the -3' to +1 subsites	Yes	Yes	Yes	Partial	Yes	Yes	Partial	No	Yes
Blood groups α-linked sugars at the -4 and -4' subsites	**Blood Group A hexasaccharide II**<br>(Supplementary Fig. 11f)	Yes<br>Reduced rate relative to LNT and LNnT.	Yes<br>Reduced rate relative to LNT and LNnT.	Partial<br>Reduced rate relative to LNT and LNnT.	No	Partial	Yes<br>Reduced rate relative to LNT and LNnT.	Yes<br>Rate unaffected relative to LNT and LNnT	No	Partial<br>Reduced rate relative to LNT and LNnT.
	**Blood Group B hexasaccharide II**<br>(Supplementary Fig. 11g)	Yes<br>Reduced rate relative to LNT and LNnT.<br>**Preference for over A**	Yes<br>Reduced rate relative to LNT and LNnT.	Yes	No	Partial	Yes<br>Reduced rate relative to LNT and LNnT.	Yes<br>Rate unaffected relative to LNT and LNnT	No	Partial<br>Reduced rate relative to LNT and LNnT.
	**Blood Group H pentasaccharide II**<br>(Supplementary Fig. 11h)	Yes<br>Improved rate relative to BGA and B	Yes<br>Same rate to BGA and B	Yes<br>Improved rate relative to BGA and B	Partial<br>Same rate to BGA and B	Yes<br>Improved rate relative to BGA and B	Yes<br>Improved rate relative to BGA and B	Yes<br>Rate unaffected relative to all	No	Partial<br>Rate unaffected relative to BGA and B

This table summarises the enzyme activities of the GH16 family members analysed in this study and characterises what sugars and linkages can be accommodated at the different subsites. This is a more detailed version of the heat map shown in Figure 4. The information is derived from Supplementary Figs. 11-13. The blue text specifically refers to the results obtained from the substrate depletion data where substrate preferences were analysed in greater detail (Supplementary Fig. 11).

Supplementary Table 5. Data collection and refinement statistics for the GH16 enzymes

	Amuc0724	BACCAC_ 02680 wt apo	BACCAC_ 02680^{E143Q} apo	BACCAC_ 02680^{E143Q} ligand	BACCAC_ 03717	BF4060 ligand	BACCAC_ 02680^{E143Q} apo
Date	25/01/19	21/09/18	21/09/18	25/11/18	11/10/18	11/10/18	21/09/18
Source	I24	I04-1	I04-1	I03	I03	I03	I04-1
Wavelength (Å)	0.9786	0.9159	0.9159	0.9793	0.9796	0.9796	0.9159
Space group	$P2_12_12_1$	$P4_1$	$P4_1$	$P4_1$	$C222_1$	$P6_122$	$P4_1$
Cell dimensions							
a, *b*, *c* (Å)	87.5, 96.1, 128.8	82.65, 82.65, 121.49	82.72, 82.72, 121.68	82.92, 82.92, 121.31	46.9, 87.1, 156.2	156.6, 156.6, 197.0	82.72, 82.72, 121.68
α, β, γ (°)	90, 90, 90	90, 90, 90	90, 90, 90	90, 90, 90	90 ,90, 90	90, 90, 120	90, 90, 90
No. of measured reflections	214047 (21796)	378511 (27291)	325288 (19162)	418545 (31263)	136701 (11117)	469646 (98047)	325288 (19162)
No. of independent reflections	33993 (4096)	51105 (3974)	47588 (3811)	55261 (4092)	19202 (1554)	22156 (4469)	47588 (3811)
Resolution (Å)	19.90 – 2.70 (2.83 – 2.70)	82.65 – 2.05 (2.16 – 2.05)	82.72 – 2.10 (2.16 – 2.10)	19.99 – 2.00 (2.05 – 2.00)	43.57– 2.10 (2.16 – 2.10)	197.05 – 3.30 (3.56 – 3.30)	82.72 – 2.10 (2.16 – 2.10)
$CC_{1/2}$	0.991 (0.485)	0.986 (0.958)	0.996 (0.557)	0.999 (0.472)	0.995 (0.743)	0.992 (0.679)	0.996 (0.557)
$I/\sigma I$	7.7 (1.0)	6.3 (1.6)	8.7 (1.6)	14.0 (1.2)	7.6 (1.6)	5.1 (1.7)	8.7 (1.6)
Completeness (%)	99.7 (100.0)	100.0 (100.0)	99.7 (97.2)	99.2 (98.4)	100.0 (100.0)	100.0 (100.0)	99.7 (97.2)
Redundancy	6.4 (5.4)	7.4 (6.9)	6.8 (5.0)	7.6 (7.6)	7.1 (7.2)	21.2 (21.9)	6.8 (5.0)
**Refinement**							
$R_{work/}$ R_{free}	20.51 / 25.90	19.94 / 24.02	18.56 /21.09	19.39 / 22.59	19.72 / 24.20	19.84 / 25.07	18.56 /21.09
No. atoms							
Protein	4450	3935	3939	3999	2035	5811	3939
Ligand/Ions	2	2	2	16	10	111	2
Water	0	134	142	99	126	0	142
B-factors							
Protein	75.2	44.3	39.5	47.4	34.1	66.1	39.5
Ligand/Ions	108.7	51.4	48.7	46.4	52.2	68.2	48.7
Water	N.A.	41.3	36.6	43.3	36.2	N.A.	36.6
R.m.s deviations							
Bond lengths (Å)	0.011	0.085	0.0098	0.011	0.010	0.011	0.0098
Bond angles (º)	2.01	1.52	1.59	1.71	1.73	2.01	1.59
Ramachandran plot (%) Favoured/Outliers	87.1 / 3.6	97.9 / 0.0	97.7 / 0.0	97.5 / 0.0	92.6 / 2.0	89.7/1.5	97.7 / 0.0
PDB	6T2N	6T2O	6T2P	6T2Q	6T2R	6T2S	6T2P

Values in parenthesis are for the highest resolution shell. Rfree was calculated using a set (5%) of randomly selected reflections that were excluded from refinement.

Supplementary Table 6. Crystallisation conditions for the GH16 enzymes

Enzyme (#)	Ligand	Condition	Cryoprotectant
Amuc_0724 (10 mg ml^{-1} l)	apo	1.6 M tri-sodium citrate pH 6.5	Paratone-N
BACCAC_02680 (8.1 mg ml^{-1})	apo	20 % PEG3350, 0.24 M sodium malonate pH7.0	25 % ethylene glycol
BACCAC_02680^{E143Q} (8.1 mg ml^{-1})	apo	24 % PEG3350, 0.45 M sodium malonate pH7.0	25 % ethylene glycol
BACCAC_02680^{E143Q} (8.1 mg ml^{-1})	TriLacNAc	24 % PEG3350, 0.45 M sodium malonate pH7.0	25 % ethylene glycol
BACCAC_03717 (10 mg ml^{-1})	apo	1.0 M Ammonium sulphate	33 % ethylene glycol
BF4060 (9.6 mg ml^{-1} l)	TriLacNAc	20 % PEG6000, 1 M lithium chloride, 0.1 M Citric acid pH 4.0	25 % ethylene glycol

Initial concentration

Supplementary Table 7. Automated conformational validation of pyranose sugar models with Privateer

Name	Sugar	Chain	Total puckering amplitude Q[1] (Å)	Phi (°)	Theta (°)	Anomer	Handeness(D/L)	Conformation	RSCC[2]	Bfactor
BACCAC_02680^{E143Q}	GAL	A	0.622	216.7	72.5	beta	D	$^{1}S_{3}$	0.47	41.6
BACCAC_02680^{E143Q}	NAG	A	0.581	298.9	5.1	beta	D	$^{4}C_{1}$	0.58	48.7
BACCAC_02680^{E143Q}	GAL	A	0.557	22.0	8.0	beta	D	$^{4}C_{1}$	0.41	55.7
BACCAC_02680^{E143Q}	GAL	B	0.690	223.4	76.9	beta	D	$^{1}S_{3}$	0.21	46.7
BACCAC_02680^{E143Q}	NAG	B	0.578	311.0	4.8	beta	D	$^{4}C_{1}$	0.21	59.0
BACCAC_02680^{E143Q}	GAL	B	0.608	78.0	17.0	beta	D	$^{4}C_{1}$	0.09	66.4
BT4060	GAL	E	0.661	190.4	78.9	beta	D	$B_{3,0}$	0.60	65.2
BT4060	NAG	E	0.564	51.8	14.0	beta	D	$^{4}C_{1}$	0.68	68.5
BT4060	GAL	E	0.535	36.4	19.4	beta	D	$^{4}C_{1}$	0.50	78.9
BT4060	GAL	F	0.658	221.7	82.3	beta	D	$^{1}S_{3}$	0.62	57.5
BT4060	NAG	F	0.583	86.1	22.8	beta	D	$^{2}H_{1}$	0.66	59.8
BT4060	GAL	F	0.658	47.1	80.3	beta	D	$B_{1,4}$	0.63	59.3
BT4060	GAL	G	0.535	303.8	9.3	beta	D	$^{4}C_{1}$	0.43	83.1
BT4060	NAG	G	0.556	73.9	9.6	beta	D	$^{4}C_{1}$	0.45	81.6
BT4060	GAL	G	0.644	1.6	77.4	beta	D	$^{3,0}B$	0.41	85.6

[1]Q is the total puckering amplitude in Angstroms.
[2]RSCC, Real Space Correlation.

Supplementary References

1. Johansson ME, Phillipson M, Petersson J, Velcich A, Holm L, Hansson GC. The inner of the two Muc2 mucin-dependent mucus layers in colon is devoid of bacteria. *Proceedings of the National Academy of Sciences of the United States of America* **105**, 15064-15069 (2008).

2. Ambort D*, et al.* Calcium and pH-dependent packing and release of the gel-forming MUC2 mucin. *Proceedings of the National Academy of Sciences of the United States of America* **109**, 5645-5650 (2012).

3. Johansson ME. Fast renewal of the distal colonic mucus layers by the surface goblet cells as measured by in vivo labeling of mucin glycoproteins. *PloS one* **7**, e41009 (2012).

4. Stephen AM, Haddad AC, Phillips SF. Passage of carbohydrate into the colon. Direct measurements in humans. *Gastroenterology* **85**, 589-595 (1983).

5. Johansson ME*, et al.* Normalization of Host Intestinal Mucus Layers Requires Long-Term Microbial Colonization. *Cell host & microbe* **18**, 582-592 (2015).

6. Hansson GC. Mucus and mucins in diseases of the intestinal and respiratory tracts. *J Intern Med*, (2019).

7. Larsson JM, Karlsson H, Sjovall H, Hansson GC. A complex, but uniform O-glycosylation of the human MUC2 mucin from colonic biopsies analyzed by nanoLC/MSn. *Glycobiology* **19**, 756-766 (2009).

8. Andersch-Bjorkman Y, Thomsson KA, Holmen Larsson JM, Ekerhovd E, Hansson GC. Large scale identification of proteins, mucins, and their O-glycosylation in the endocervical mucus during the menstrual cycle. *Molecular & cellular proteomics : MCP* **6**, 708-716 (2007).

9. Thomsson KA, Schulz BL, Packer NH, Karlsson NG. MUC5B glycosylation in human saliva reflects blood group and secretor status. *Glycobiology* **15**, 791-804 (2005).

10. Schulz BL*, et al.* Mucin glycosylation changes in cystic fibrosis lung disease are not manifest in submucosal gland secretions. *The Biochemical journal* **387**, 911-919 (2005).

11. Fujita M*, et al.* Glycoside hydrolase family 89 alpha-N-acetylglucosaminidase from Clostridium perfringens specifically acts on GlcNAc alpha1,4Gal beta1R at the non-reducing terminus of O-glycans in gastric mucin. *The Journal of biological chemistry* **286**, 6479-6489 (2011).

12. Nordman H, Davies JR, Lindell G, de Bolos C, Real F, Carlstedt I. Gastric MUC5AC and MUC6 are large oligomeric mucins that differ in size, glycosylation and tissue distribution. *The Biochemical journal* **364**, 191-200 (2002).

13. Jin C*, et al.* Structural Diversity of Human Gastric Mucin Glycans. *Molecular & cellular proteomics : MCP* **16**, 743-758 (2017).

14. Cartmell A*, et al.* How members of the human gut microbiota overcome the sulfation problem posed by glycosaminoglycans. *Proceedings of the National Academy of Sciences of the United States of America* **114**, 7037-7042 (2017).

15. Ndeh D*, et al.* The human gut microbe Bacteroides thetaiotaomicron encodes the founding member of a novel glycosaminoglycan-degrading polysaccharide lyase family PL29. *The Journal of biological chemistry* **293**, 17906-17916 (2018).

16. Martens EC, Chiang HC, Gordon JI. Mucosal glycan foraging enhances fitness and transmission of a saccharolytic human gut bacterial symbiont. *Cell host & microbe* **4**, 447-457 (2008).

17. Pudlo NA, Urs K, Kumar SS, German JB, Mills DA, Martens EC. Symbiotic Human Gut Bacteria with Variable Metabolic Priorities for Host Mucosal Glycans. *mBio* **6**, e01282-01215 (2015).

18. Desai MS*, et al.* A Dietary Fiber-Deprived Gut Microbiota Degrades the Colonic Mucus Barrier and Enhances Pathogen Susceptibility. *Cell* **167**, 1339-1353.e1321 (2016).

19. Ottman N*, et al.* Genome-Scale Model and Omics Analysis of Metabolic Capacities of Akkermansia muciniphila Reveal a Preferential Mucin-Degrading Lifestyle. *Applied and environmental microbiology* **83**, (2017).

20. Shin J*, et al.* Elucidation of Akkermansia muciniphila Probiotic Traits Driven by Mucin Depletion. *Frontiers in microbiology* **10**, 1137 (2019).

21. Ottman N*, et al.* Characterization of Outer Membrane Proteome of Akkermansia muciniphila Reveals Sets of Novel Proteins Exposed to the Human Intestine. *Frontiers in microbiology* **7**, 1157 (2016).

22. Kitamikado M, Ito M, Li YT. Isolation and characterization of a keratan sulfate-degrading endo-beta-galactosidase from Flavobacterium keratolyticus. *The Journal of biological chemistry* **256**, 3906-3909 (1981).

23. Ito M, Hirabayashi Y, Yamagata T. Substrate specificity of endo-beta-galactosidases from Flavobacterium keratolyticus and Escherichia freundii is different from that of Pseudomonas sp. *Journal of biochemistry* **100**, 773-780 (1986).

24. Cartmell A*, et al.* A surface endogalactanase in Bacteroides thetaiotaomicron confers keystone status for arabinogalactan degradation. *Nature microbiology* **3**, 1314-1326 (2018).

25. Katayama T*, et al.* Molecular cloning and characterization of Bifidobacterium bifidum 1,2-alpha-L-fucosidase (AfcA), a novel inverting glycosidase (glycoside hydrolase family 95). *Journal of bacteriology* **186**, 4885-4893 (2004).

26. Briliute J*, et al.* Complex N-glycan breakdown by gut Bacteroides involves an extensive enzymatic apparatus encoded by multiple co-regulated genetic loci. *Nature microbiology* **4**, 1571-1581 (2019).

27. Hehemann JH, Correc G, Barbeyron T, Helbert W, Czjzek M, Michel G. Transfer of carbohydrate-active enzymes from marine bacteria to Japanese gut microbiota. *Nature* **464**, 908-912 (2010).

28. Hehemann JH, Kelly AG, Pudlo NA, Martens EC, Boraston AB. Bacteria of the human gut microbiome catabolize red seaweed glycans with carbohydrate-active enzyme updates from extrinsic microbes. *Proceedings of the National Academy of Sciences of the United States of America* **109**, 19786-19791 (2012).

29. Abro AH, Rahimi Shahmirzadi MR, Jasim LM, Badreddine S, Al Deesi Z. Sphingobacterium multivorum Bacteremia and Acute Meningitis in an Immunocompetent Adult Patient: A Case Report. *Iranian Red Crescent medical journal* **18**, e38750 (2016).

30. Ashida H*, et al.* A novel endo-beta-galactosidase from Clostridium perfringens that liberates the disaccharide GlcNAcalpha 1-->Gal from glycans specifically expressed in the gastric gland mucous cell-type mucin. *The Journal of biological chemistry* **276**, 28226-28232 (2001).

31. Ashida H, Maskos K, Li SC, Li YT. Characterization of a novel endo-beta-galactosidase specific for releasing the disaccharide GlcNAc alpha 1-->4Gal from glycoconjugates. *Biochemistry* **41**, 2388-2395 (2002).

32. Nakayama J, Yeh JC, Misra AK, Ito S, Katsuyama T, Fukuda M. Expression cloning of a human alpha1, 4-N-acetylglucosaminyltransferase that forms GlcNAcalpha1-->4Galbeta-->R, a glycan specifically expressed in the gastric gland mucous cell-type mucin. *Proceedings of the National Academy of Sciences of the United States of America* **96**, 8991-8996 (1999).

33. Ishihara K*, et al.* Peripheral alpha-linked N-acetylglucosamine on the carbohydrate moiety of mucin derived from mammalian gastric gland mucous cells: epitope recognized by a newly characterized monoclonal antibody. *The Biochemical journal* **318 (Pt 2)**, 409-416 (1996).

34. Tempel W*, et al.* Three-dimensional structure of GlcNAcalpha1-4Gal releasing endo-beta-galactosidase from Clostridium perfringens. *Proteins* **59**, 141-144 (2005).

35. Yamagishi K*, et al.* Purification, characterization, and molecular cloning of a novel keratan sulfate hydrolase, endo-beta-N-acetylglucosaminidase, from Bacillus circulans. *The Journal of biological chemistry* **278**, 25766-25772 (2003).

36. Wang H*, et al.* Construction and functional characterization of truncated versions of recombinant keratanase II from Bacillus circulans. *Glycoconjugate journal* **34**, 643-649 (2017).

37. Fukuda MN, Matsumura G. Endo-beta-galactosidase of Escherichia freundii. Purification and endoglycosidic action on keratan sulfates, oligosaccharides, and blood group active glycoprotein. *The Journal of biological chemistry* **251**, 6218-6225 (1976).

38. Scudder P, Uemura K, Dolby J, Fukuda MN, Feizi T. Isolation and characterization of an endo-beta-galactosidase from Bacteroides fragilis. *The Biochemical journal* **213**, 485-494 (1983).

39. Hirano S, Meyer K. Enzymatic degradation of corneal and cartilaginous keratosulfates. *Biochemical and biophysical research communications* **44**, 1371-1375 (1971).

40. Agirre J, Iglesias-Fernandez J, Rovira C, Davies GJ, Wilson KS, Cowtan KD. Privateer: software for the conformational validation of carbohydrate structures. *Nature structural & molecular biology* **22**, 833-834 (2015).

41. Biarnes X, Ardevol A, Planas A, Rovira C, Laio A, Parrinello M. The conformational free energy landscape of beta-D-glucopyranose. Implications for substrate preactivation in beta-glucoside hydrolases. *Journal of the American Chemical Society* **129**, 10686-10693 (2007).

42. Labourel A*, et al.* Structural and biochemical characterization of the laminarinase ZgLamCGH16 from Zobellia galactanivorans suggests preferred recognition of branched laminarin. *Acta crystallographica Section D, Biological crystallography* **71**, 173-184 (2015).

43. Allouch J, Helbert W, Henrissat B, Czjzek M. Parallel substrate binding sites in a beta-agarase suggest a novel mode of action on double-helical agarose. *Structure (London, England : 1993)* **12**, 623-632 (2004).

44. Vasur J*, et al.* X-ray crystal structures of Phanerochaete chrysosporium Laminarinase 16A in complex with products from lichenin and laminarin hydrolysis. *The FEBS journal* **276**, 3858-3869 (2009).

45. Matard-Mann M*, et al.* Structural insights into marine carbohydrate degradation by family GH16 kappa-carrageenases. *The Journal of biological chemistry* **292**, 19919-19934 (2017).

46. Davies GJ*, et al.* Snapshots along an enzymatic reaction coordinate: analysis of a retaining beta-glycoside hydrolase. *Biochemistry* **37**, 11707-11713 (1998).

47. Davies GJ, Tolley SP, Henrissat B, Hjort C, Schulein M. Structures of oligosaccharide-bound forms of the endoglucanase V from Humicola insolens at 1.9 A resolution. *Biochemistry* **34**, 16210-16220 (1995).

48. Tamura K*, et al.* Molecular Mechanism by which Prominent Human Gut Bacteroidetes Utilize Mixed-Linkage Beta-Glucans, Major Health-Promoting Cereal Polysaccharides. *Cell Rep* **21**, 417-430 (2017).

49. Hehemann JH*, et al.* Biochemical and structural characterization of the complex agarolytic enzyme system from the marine bacterium Zobellia galactanivorans. *The Journal of biological chemistry* **287**, 30571-30584 (2012).
