# Supplementary material for: Prominent members of the human gut microbiota express endo-acting O-glycanases to initiate mucin breakdown
Source: Nat Commun. 2020 Aug 11;11:4017. doi: 10.1038/s41467-020-17847-5 (PMC7419316; doi:10.1038/s41467-020-17847-5)
Supplement: Supplementary file 3 — Reporting Summary [file 41467_2020_17847_MOESM3_ESM.pdf]

nature research

Corresponding author(s): Lucy Crouch
David Bolam

Last updated by author(s): 2020/05/23

Reporting Summary

Nature Research wishes to improve the reproducibility of the work that we publish. This form provides structure for consistency and transparency in reporting. For further information on Nature Research policies, see Authors & Referees and the Editorial Policy Checklist .

Statistics

For all statistical analyses, confirm that the following items are present in the figure legend, table legend, main text, or Methods section.

n/a	Confirmed	
☐	☒	The exact sample size (*n*) for each experimental group/condition, given as a discrete number and unit of measurement
☐	☒	A statement on whether measurements were taken from distinct samples or whether the same sample was measured repeatedly
☒	☐	The statistical test(s) used AND whether they are one- or two-sided<br>*Only common tests should be described solely by name; describe more complex techniques in the Methods section.*
☒	☐	A description of all covariates tested
☒	☐	A description of any assumptions or corrections, such as tests of normality and adjustment for multiple comparisons
☐	☒	A full description of the statistical parameters including central tendency (e.g. means) or other basic estimates (e.g. regression coefficient) AND variation (e.g. standard deviation) or associated estimates of uncertainty (e.g. confidence intervals)
☒	☐	For null hypothesis testing, the test statistic (e.g. *F*, *t*, *r*) with confidence intervals, effect sizes, degrees of freedom and *P* value noted<br>*Give P values as exact values whenever suitable.*
☒	☐	For Bayesian analysis, information on the choice of priors and Markov chain Monte Carlo settings
☒	☐	For hierarchical and complex designs, identification of the appropriate level for tests and full reporting of outcomes
☒	☐	Estimates of effect sizes (e.g. Cohen's *d*, Pearson's *r*), indicating how they were calculated

Our web collection on statistics for biologists contains articles on many of the points above.

Software and code

Policy information about availability of computer code

Data collection	Structural Data Collection: XDS (Mar 2019), DIALS 1.14.5, XIA2 0.5653. HPLC and Mass Spec Data Collaction: Chromeleon Chromatography Data System Software, HyStar v3.2.
Data analysis	Graphpad Prism 8, Bruker Compass Data Analysis 4.4, GlycoWorkBench, ClustalOmega, IMG database, CAZy, SeaView, Pfam, SMART, Pymol 1.8.x, Pointless 1.11.19, Aimless 0.7.4, MolRep 11.6.04, Phaser2.8.2, Phenix 1.18.3855, Coot 0.8.9.1, CCP4 suite 7.0, Privateer MKIII

For manuscripts utilizing custom algorithms or software that are central to the research but not yet described in published literature, software must be made available to editors/reviewers. We strongly encourage code deposition in a community repository (e.g. GitHub). See the Nature Research guidelines for submitting code & software for further information.

Data

Policy information about availability of data

All manuscripts must include a data availability statement. This statement should provide the following information, where applicable:
- Accession codes, unique identifiers, or web links for publicly available datasets
- A list of figures that have associated raw data
- A description of any restrictions on data availability

The data that support the findings in this paper are available upon request from the corresponding authors.

Field-specific reporting

Please select the one below that is the best fit for your research. If you are not sure, read the appropriate sections before making your selection.

☒ Life sciences ☐ Behavioural & social sciences ☐ Ecological, evolutionary & environmental sciences

Life sciences study design

All studies must disclose on these points even when the disclosure is negative.

Sample size	No sample size calculation was performed. Non-kinetic enzyme assays were repeated at least once for each substrate tested with different enzyme preparations. Bacterial growths are averages of triplicate cultures and were repeated at least once.
Data exclusions	No data were excluded.
Replication	Bacterial growth experiments on different glycans were carried out in triplicate and the experiments repeated at least once. The non-kinetic assays on recombinant enzymes were reproduced multiple times with different enzyme preparations on each of the different substrates tested. Where possible, positive and negative controls for enzyme assays were used throughout to be sure of the accuracy of results and experimental conditions. LC-MS/MS experiments were repeated at least once independently and the labelled glycans from a single enzyme assay often analysed twice to ensure the data was reproducible and in some cases optimise detection. In all cases replicates were consistent and the data was analysed independently by two people (Dr Lucy Crouch and Paulina Urbanowicz).
Randomization	N/A
Blinding	N/A

Reporting for specific materials, systems and methods

We require information from authors about some types of materials, experimental systems and methods used in many studies. Here, indicate whether each material, system or method listed is relevant to your study. If you are not sure if a list item applies to your research, read the appropriate section before selecting a response.

Materials & experimental systems

n/a	Involved in the study	
☒	☐	Antibodies
☒	☐	Eukaryotic cell lines
☒	☐	Palaeontology
☒	☐	Animals and other organisms
☐	☒	Human research participants
☒	☐	Clinical data

Methods

n/a	Involved in the study	
☒	☐	ChIP-seq
☒	☐	Flow cytometry
☒	☐	MRI-based neuroimaging

Human research participants

Policy information about studies involving human research participants

Population characteristics	N/A
Recruitment	For both the IBD and NEC samples, the patients were not chosen, but were just the next ones that required surgery after we had all the ethical approvals in place. Only a small number of patients were included as this was only a proof-of-principle study.
Ethics oversight	Newcastle and North Tyneside Research Ethics Committee 1 (REC:17/NE/0361) and SERVIS study (approvals 10/H0908/39 and 15-NE-0334)

Note that full information on the approval of the study protocol must also be provided in the manuscript.
