# Supplementary material for: Prominent members of the human gut microbiota express endo-acting O-glycanases to initiate mucin breakdown
Source: Nat Commun. 2020 Aug 11;11:4017. doi: 10.1038/s41467-020-17847-5 (PMC7419316; doi:10.1038/s41467-020-17847-5)

Source Data File

Full image disclosure of the TLC and gel images from all main and supplementary Figures.
Orange dotted boxes indicate the regions used in the figure

Figure 5

a

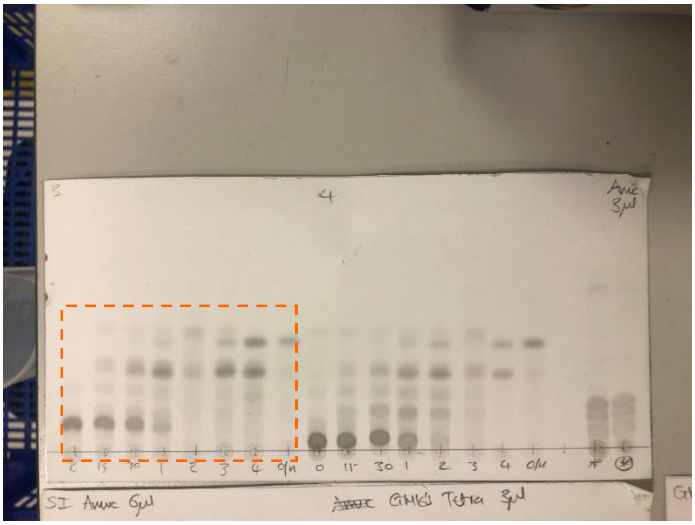

b

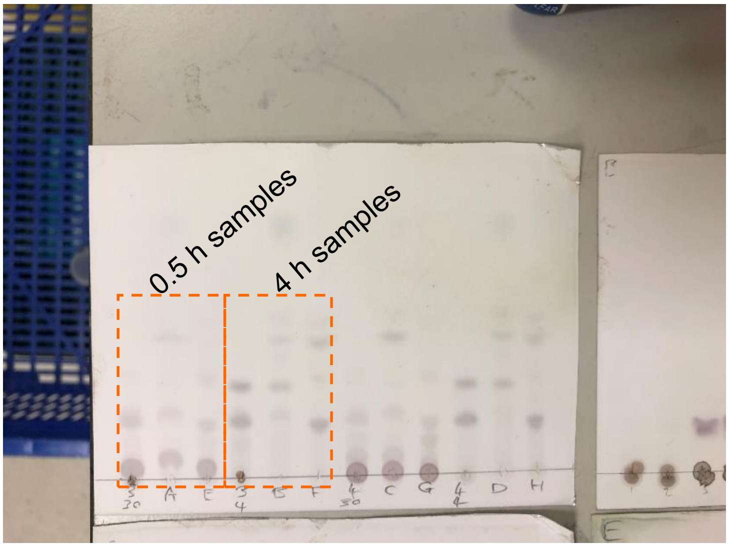

Supplementary Figure 7

a

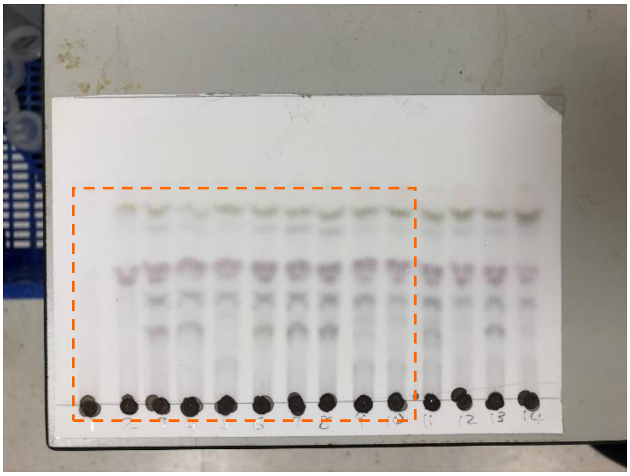

b

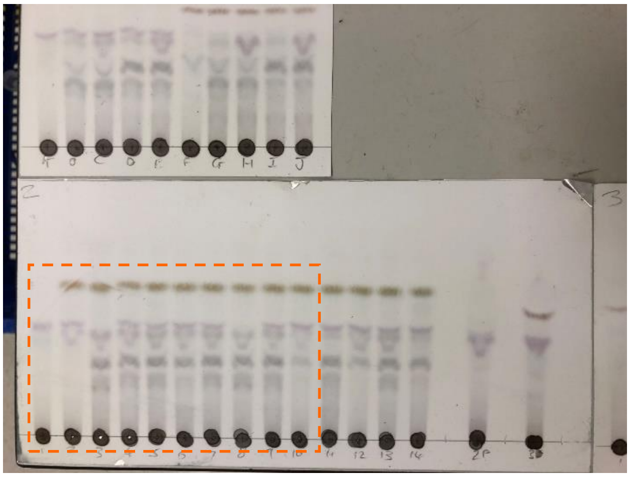

c

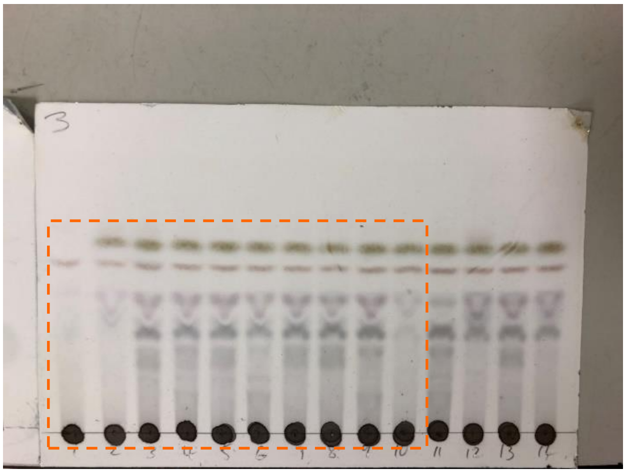

Supplementary Figure 9

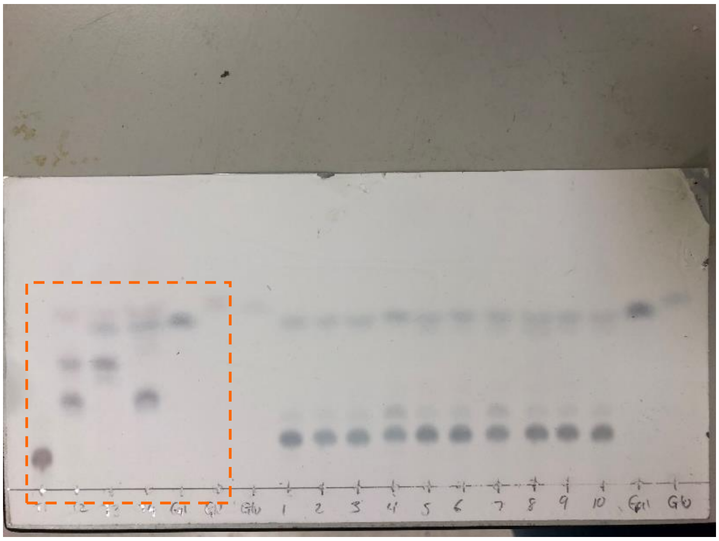

Supplementary Figure 11

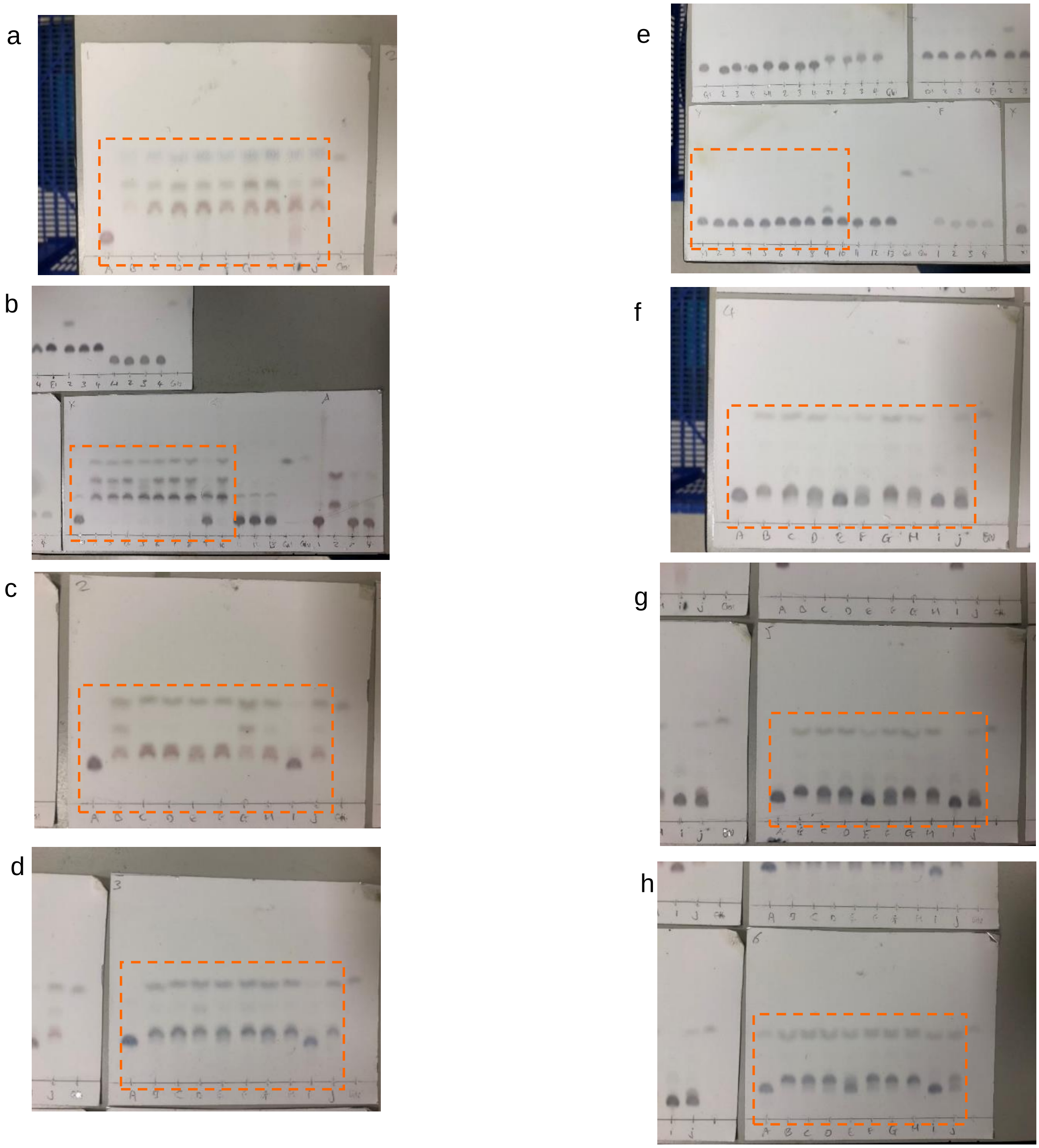

Supplementary Figure 12

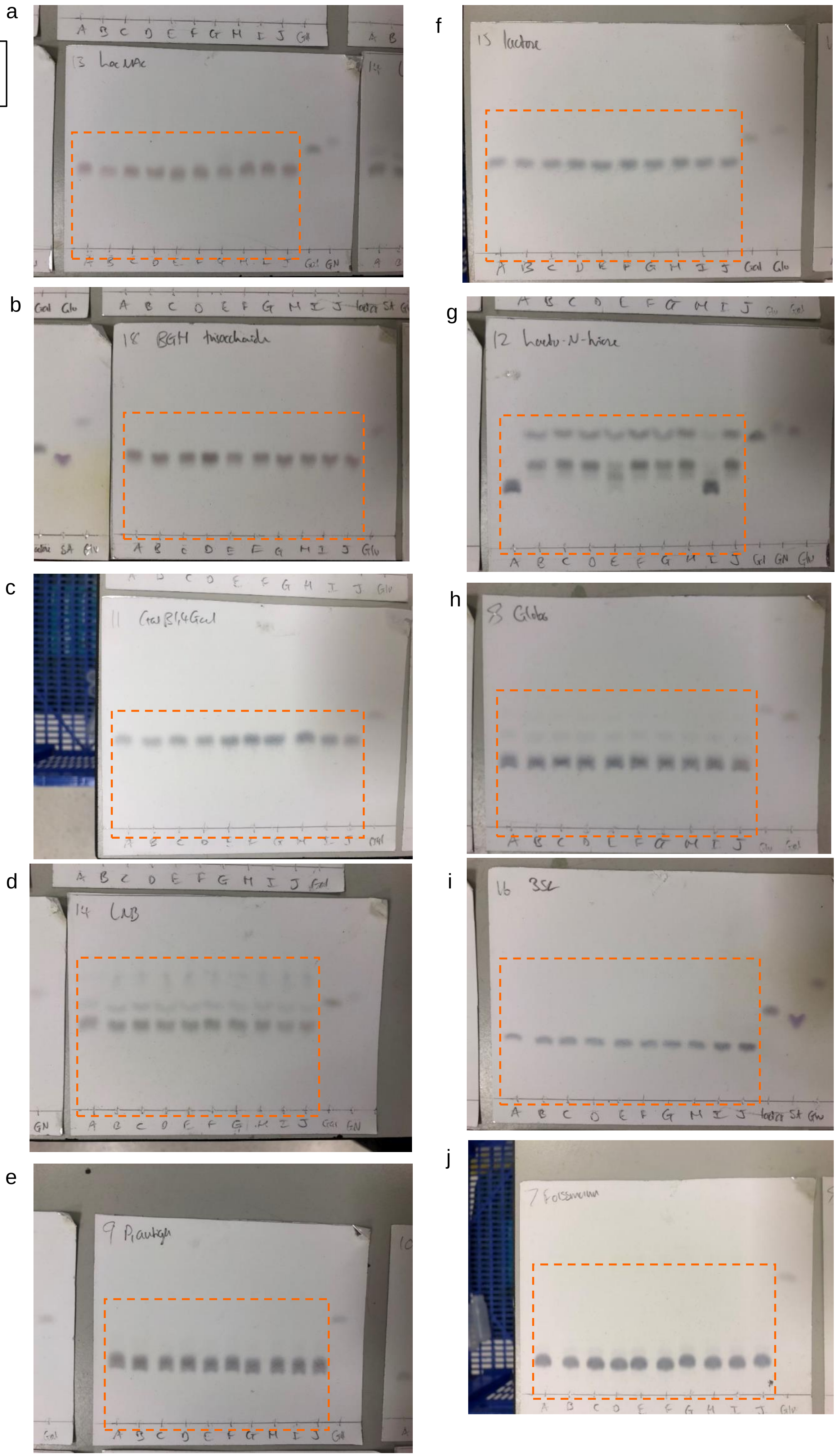

Supplementary Figure 13

a

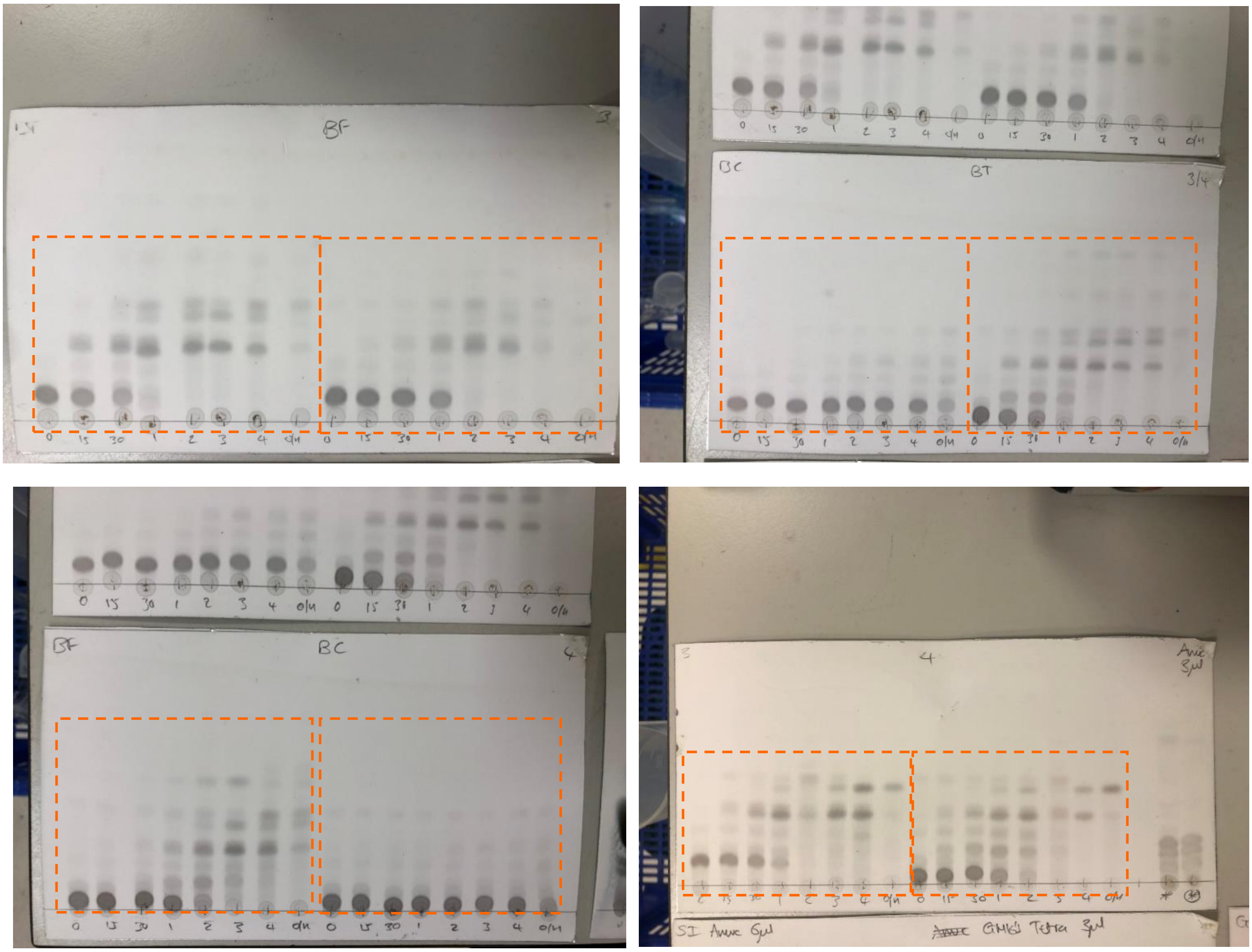

c

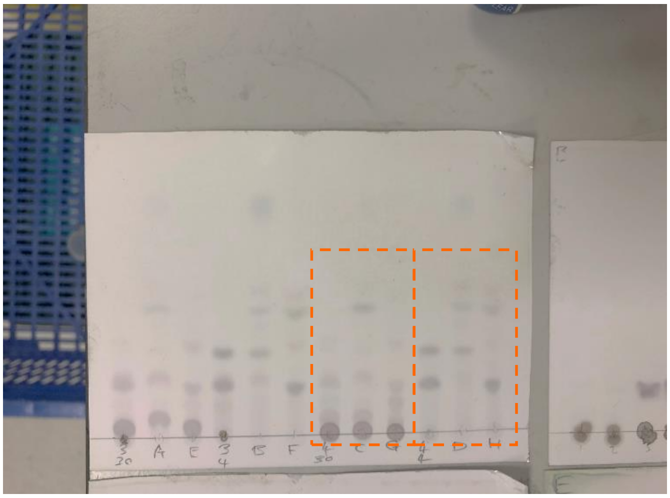

Supplementary Figure 14

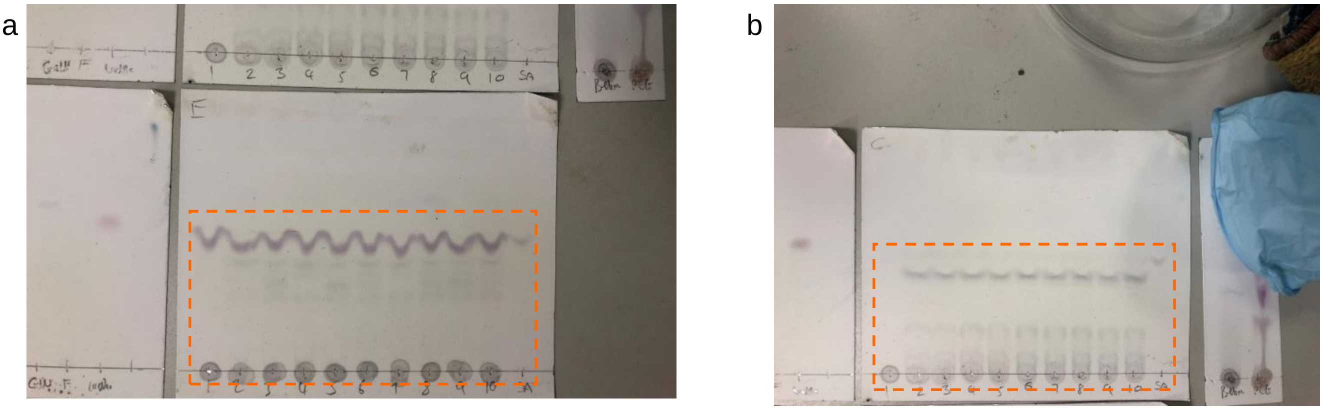

Supplementary Figure 15

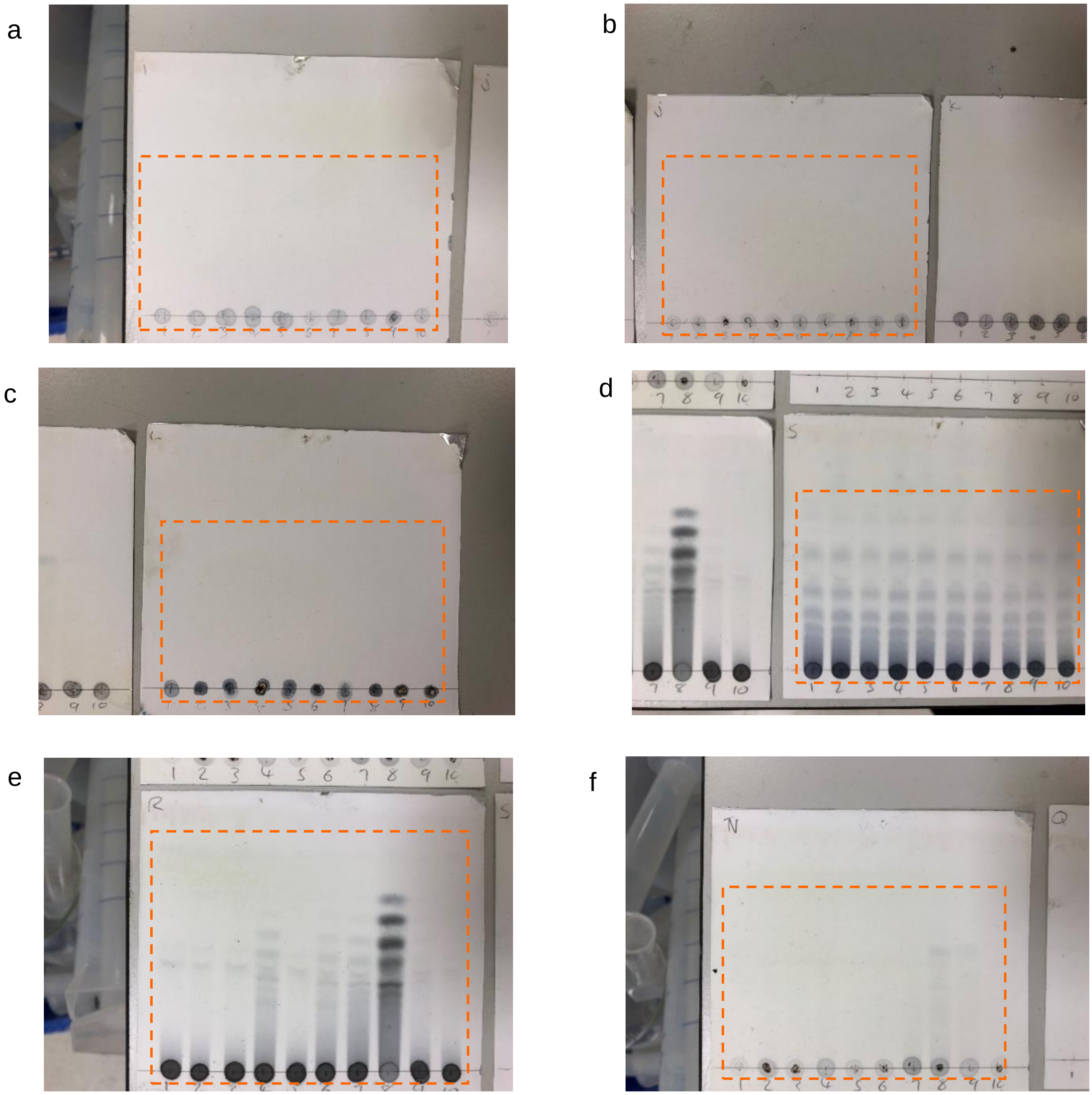

Supplementary Figure 15

g

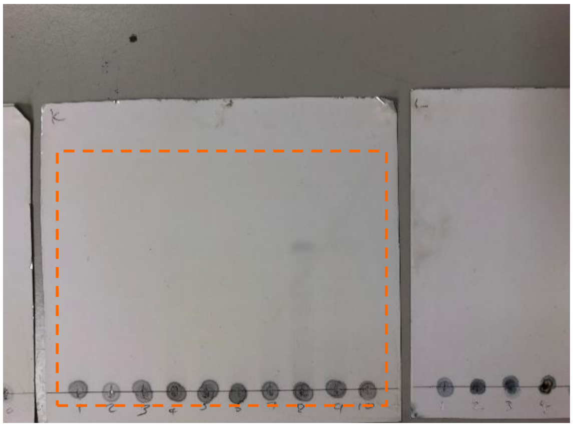

h

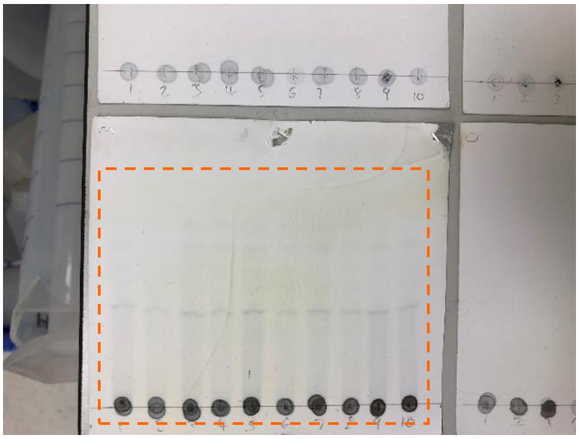

i

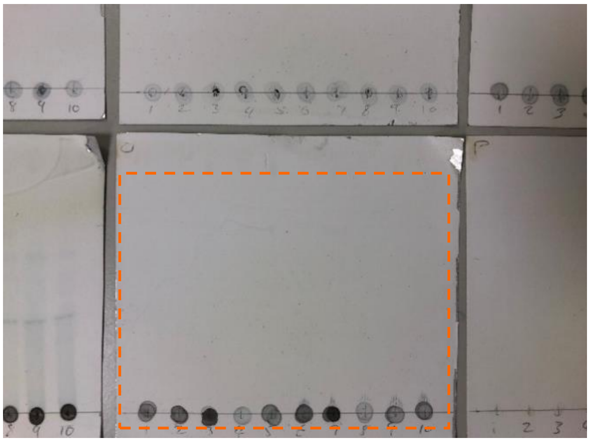

j

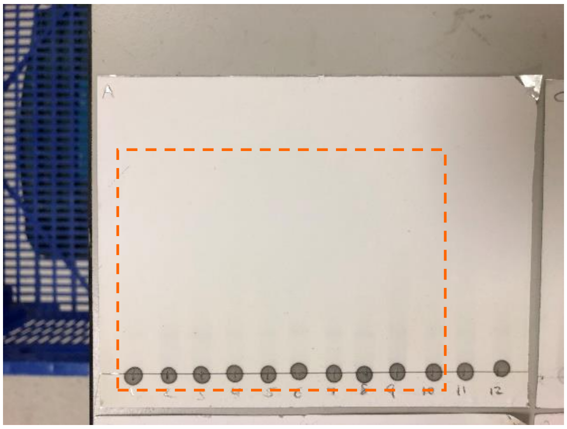

k

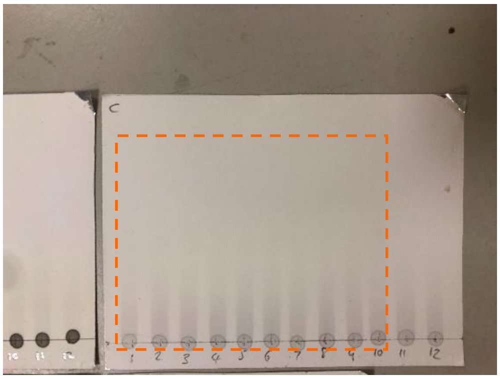

l

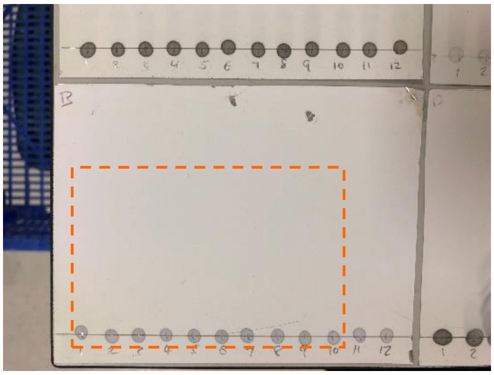

m

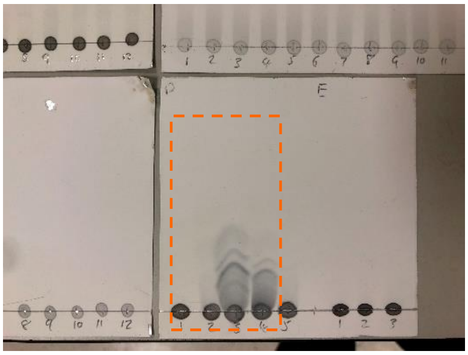

n

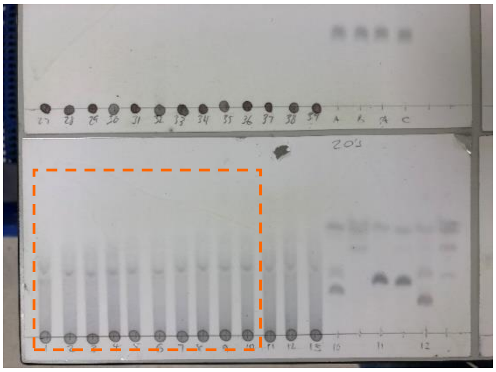

o

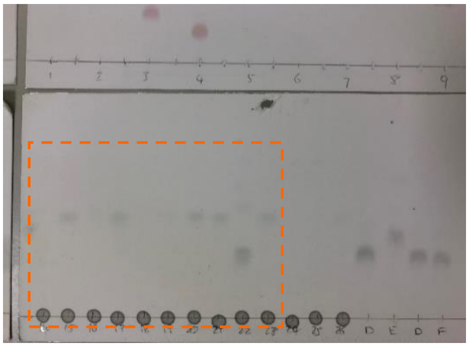

p

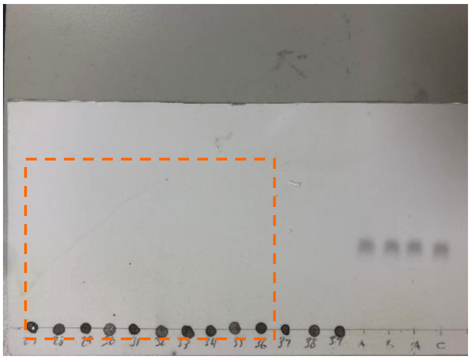

Supplementary Figure 15

q

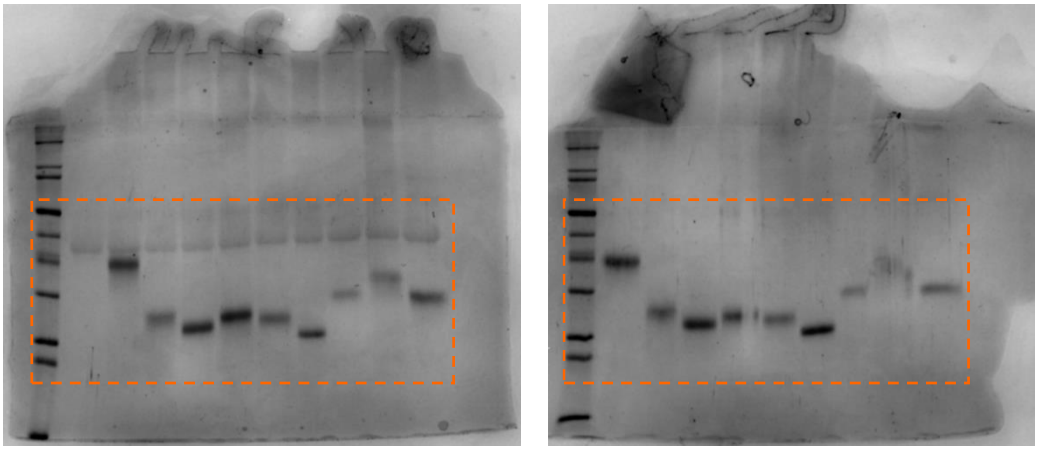

r

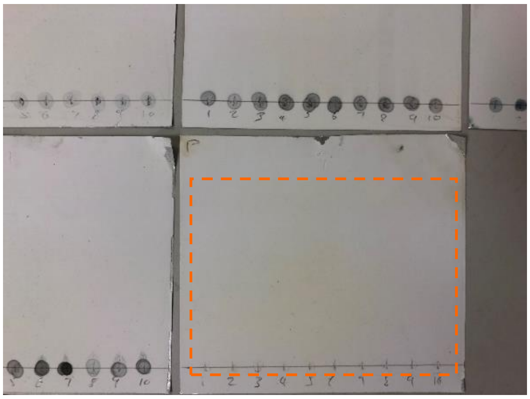

s

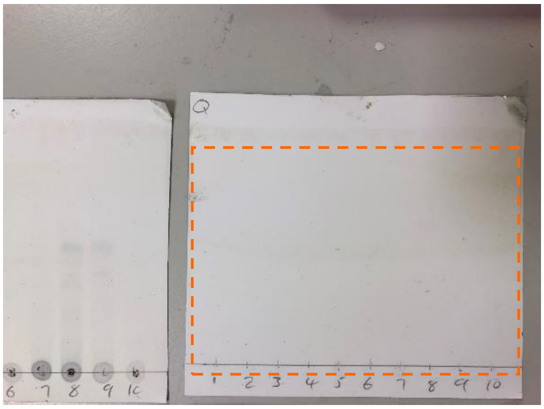

Supplementary Figure 16

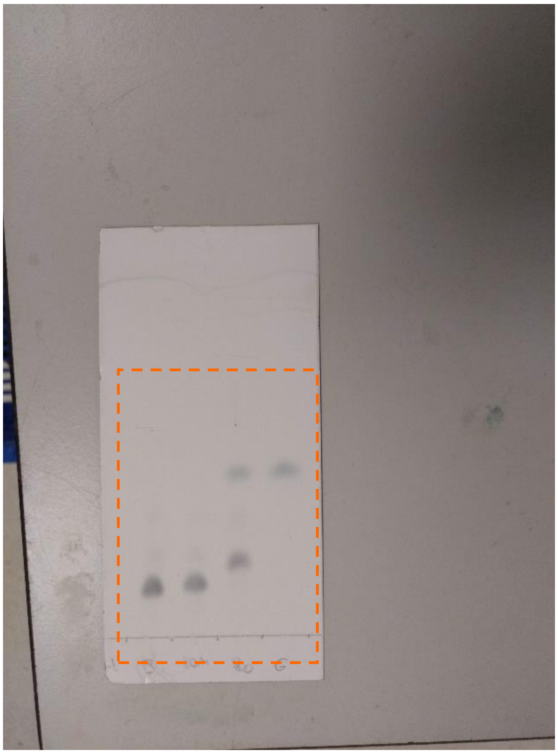

Supplementary Figure 20

a

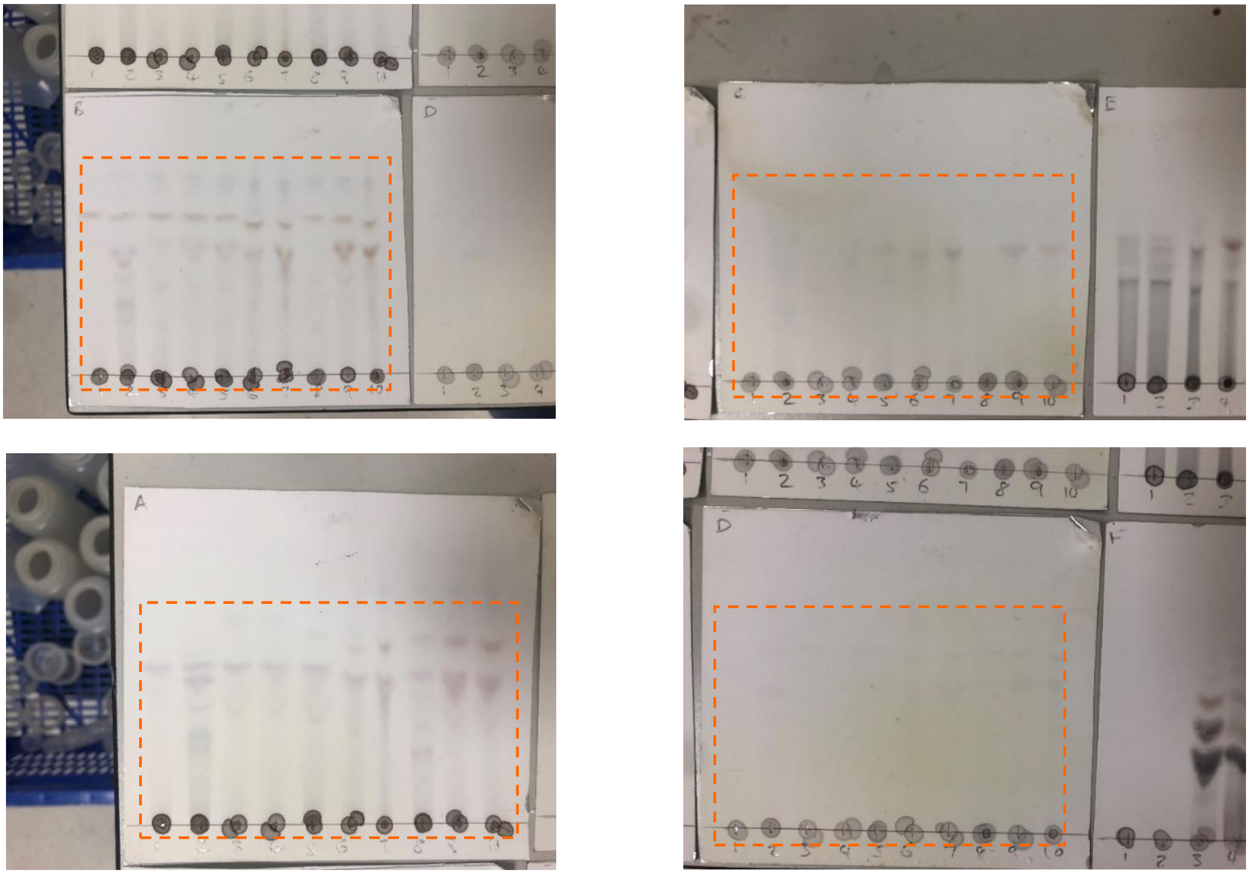

b

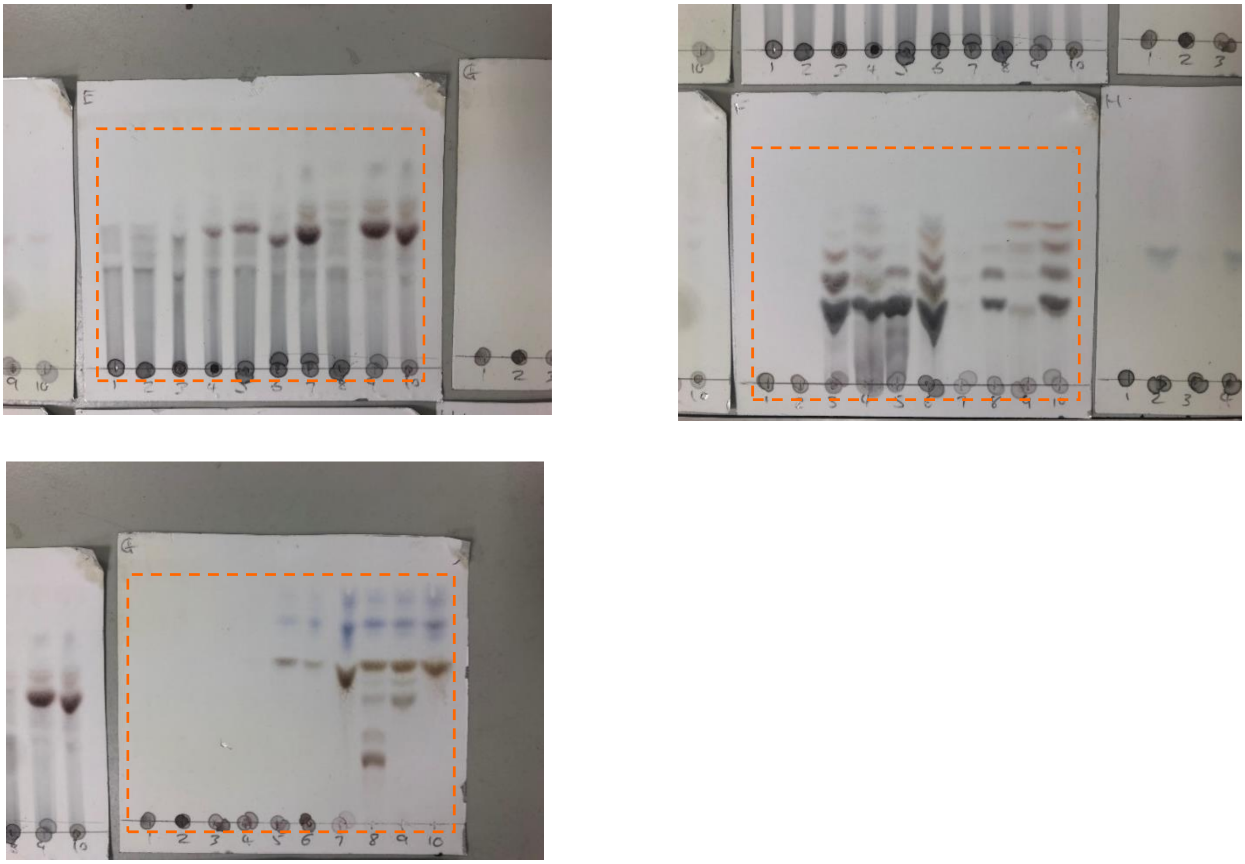

Supplementary Figure 24

a

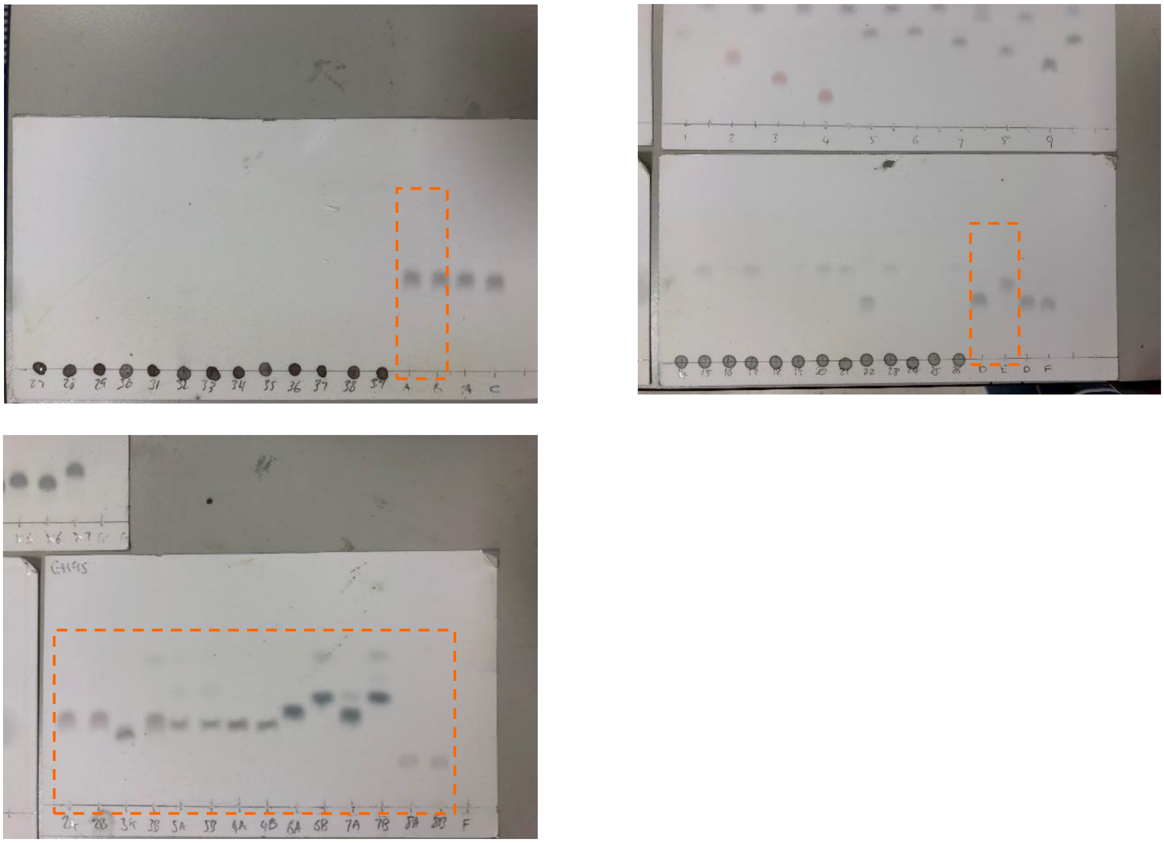

b

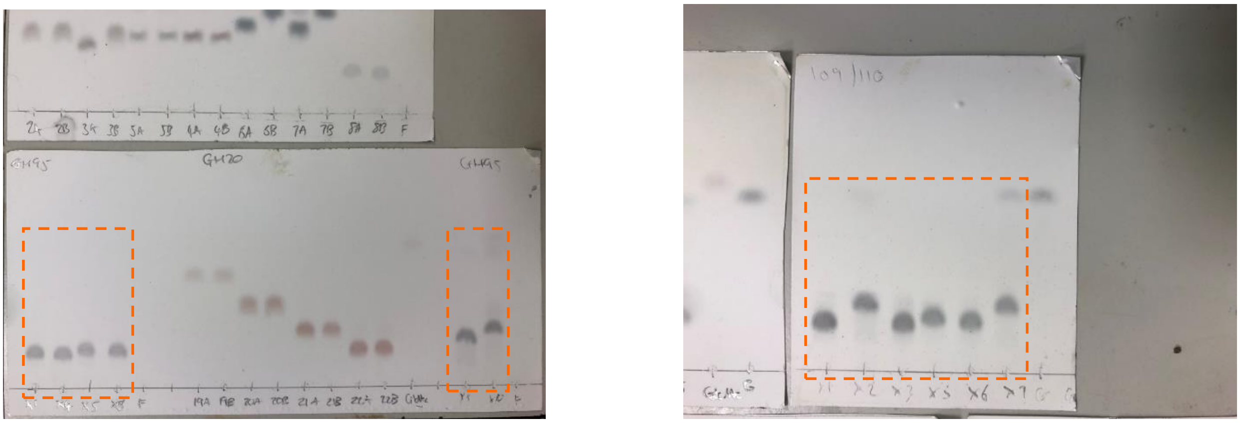

Supplementary Figure 25

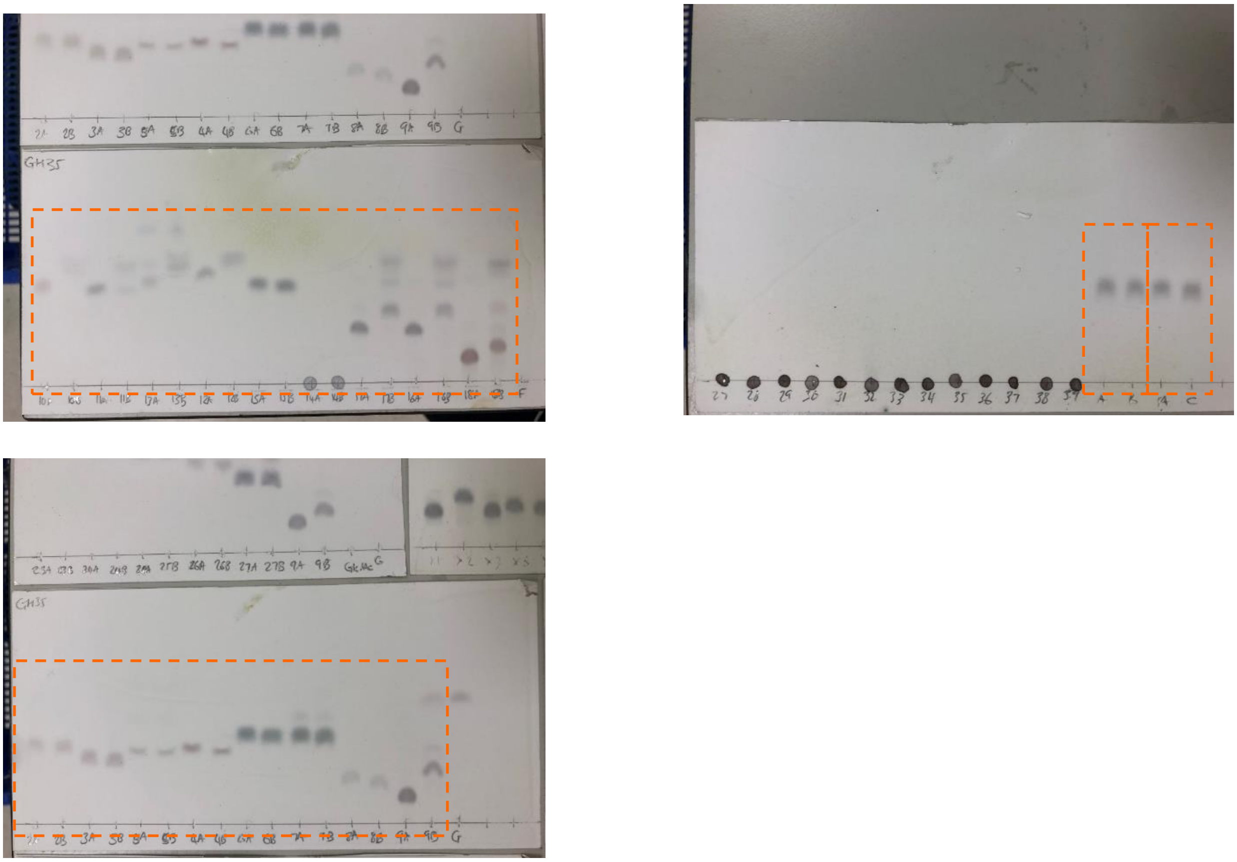

Supplementary Figure 26

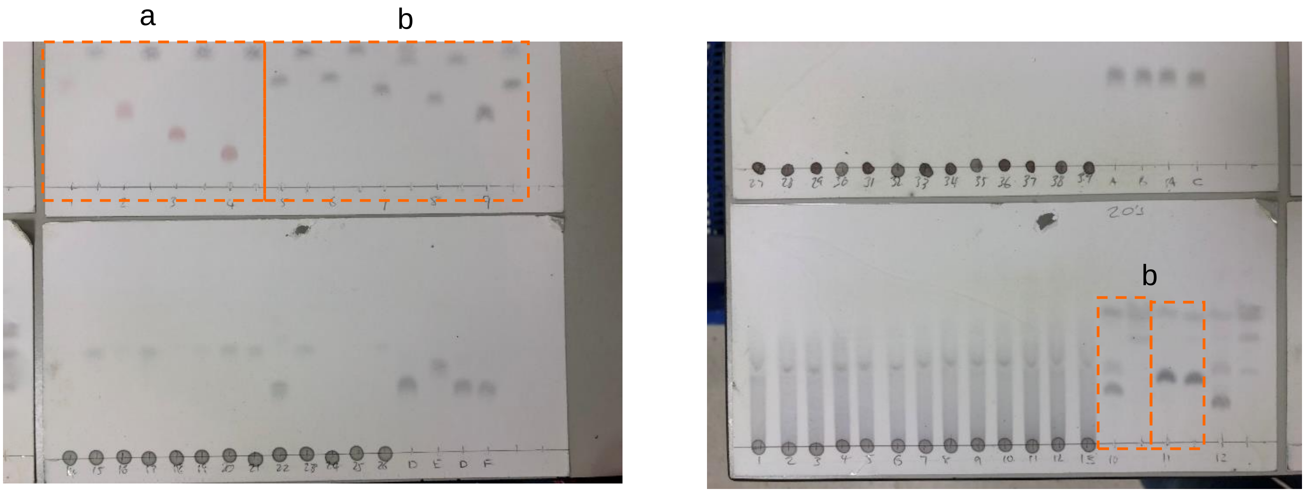

Supplement: Supplementary file 4 — Source Data [file 41467_2020_17847_MOESM4_ESM.zip › Source Data Folder/Source data TLC and gels.pdf]
